# Effect of triage training on the knowledge application and practice improvement among the practicing nurses of the emergency departments of the National Referral Hospitals, 2018; a pre-post study in Asmara, Eritrea

**DOI:** 10.1186/s12873-022-00755-w

**Published:** 2022-12-02

**Authors:** Teklu Tsegai Bahlibi, Eyasu Habte Tesfamariam, Yonatan Mehari Andemeskel, Ghidey Gebreyohannes Weldegiorgis

**Affiliations:** 1 Orotta Medical Surgical National Referral Teaching Hospital, Asmara, Eritrea; 2 Department of Statistics, Biostatistics & Epidemiology Unit, College of Science, Eritrean Institute of Technology Mai-Nefhi, May-Nefhi, Eritrea; 3 Orotta College of Medicine and Health Sciences, Asmara, Eritrea

**Keywords:** Emergency Care, Emergency Nurses, Knowledge, Triage training, Triage practice

## Abstract

**Background:**

Triage starts at the front door of the Emergency Department (ED), and repeatedly performed by the responsible duty nurses with the aim of facilitating a timely and appropriate treatment of patients. A triage system called the Orotta Triage System was implemented in the emergency settings of the selected hospitals in 2006, with the emergency nurses trained to triage using the system. Since the introduction, a majority of nurses have been replaced by new untrained nurses. This study was conducted to assess the impact of an educational intervention on the triage nurses knowledge and performance.

**Methods:**

A single group pre-posttest study design was performed in the adult EDs of the National Referral Eritrean Referral Hospitals, from January to July of 2018. All staff members in the ED were involved. Data collection tools utilized were, a self-administered knowledge assessing questionnaire and a practice observation checklist. Analysis was done in SPSS (version 22) using repeated measures ANOVA. Statistical significance level was set at *P* < 0.05.

**Results:**

The mean knowledge scores at Time 1(prior to the intervention), Time 2 (following the intervention) and Time 3 (three month follow up) were 6.23 (SD = 2.29), 10.55 (SD = 1.79), and 9.39(SD = 2.67) respectively. During the pre-intervention phase, only one (3%) nurse was determined to have adequate knowledge. Two days post training (immediate post-intervention), the percentage possessing adequate knowledge increased to 39% but dropped back to 19% three months later. Mean knowledge difference scores (95% CI) of immediate post and pre-intervention (Diff. = 4.32, 1 95%CI: 3.08–5.56), three months later and pre-intervention (Diff. = 3.16, 95%CI: 1.71–4.62) and immediate post and three months later (Diff. = 1.16, 95%CI: 0.12–2.20) were found to be statistically significant. The median score of appropriate triage practice at pre-intervention (Md = 6, IQR = 3) was not significantly different (*p* = 0.053) from that at post-intervention (Md = 8, IQR = 5).

**Conclusion:**

The level of triage knowledge and appropriate application was low among the emergency nurses prior to training. The training provided an initial improvement in knowledge, but no significant improvement in triage nursing performance. To optimize ED triage performance, appropriate, timely in-service training is required to ensure new staff are educated and experienced staff have their knowledge and skills refreshed.

**Supplementary Information:**

The online version contains supplementary material available at 10.1186/s12873-022-00755-w.

## Background

Triage is an important component of emergency department care. It is designed to help identify and prioritize undifferentiated patients based on severity and risk into categories from emergent to non-urgent. The triage nurse in the emergency department is the first person who encounters emergency patients, thus it is an essential factor that the emergency nurse has the knowledge and experience. Moreover, triage begins upon entry to the emergency department and needs to be reevaluated as the patient waits or moves through the system, to ensure the appropriate speed and level of care is being provided reliably and safely [1–3]. Studies have shown that non-urgent emergency visits may led to unnecessary cost and multiple adverse consequences [4–7]. Triage is an important component of emergency medical practice especially in places where there is a mismatch between medical demands and available resources [2]. This highlights the importance of adequate knowledge, skill and expertise for triage nurses. Lack of such triage knowledge and skills can lead to inconsistencies in the provision of appropriate and timely care [8–11].

Triage nurses are expected to be competent and operating autonomously in their triage role. Triage training is considered essential, underpinned by the assumption that improved triage knowledge will improve triage decision making [1, 11–15]. Nursing knowledge and practice has reportedly been found to have improved post triage training. [8, 9, 12, 14, 15]. Given its importance, many studies have emphasized that providing regular training programs allows triage nurses to operate more effectively, thereby enhancing patient safety and the quality of emergency care [1, 8–10, 12, 14, 16–21]. Moreover, there is an expectation that emergency department leadership ensures that nurses receive regular and appropriate training required to successfully function in the role of triage nurse according to their professional standards [22].

Most hospitals in low income countries lack a formal triage system [23]. In Eritrea, great steps have been made in the past ten years towards establishing a Hospital Triage System. A triage scale called the Orotta Triage System (OTS) was developed in 2006. The triage system was originally derived from the Manchester Triage Scale (MTS). The MTS is a five level triage scale, however, it was determined that we only required a four-level scale. Since 2006, triage training to the national hospital nurses has only been offered once. Since then, however, there has been significant nurse turnover, with no process or program in place to educate these untrained triage nurses. This study was introduced to evaluate the effect of a new triage training program on knowledge retention and the performance of emergency triage nurses in the national referral hospitals.

## Method

### Study design & setting

This was a single group pre-posttest study design conducted between January and July of 2018. The study was conducted in Orotta and Halibet EDs, located in Asmara, the capital of Eritrea. These Hospitals are the country’s Medical Surgical National Referral Hospitals, providing healthcare to medical and surgical patients from all zones of Eritrea. These Hospitals were chosen because they are the major referral centers of the country. They are public Hospitals where self-referred residents of Asmara also receive treatment and follow-up. A variety of medical and surgical cases, including trauma starting from road traffic accident to mass causalities, occupational accidents, burns and acute medical illnesses all receive care.

### Study participants

The study included a total of 33 nurses who work in the emergency departments of the aforementioned hospitals. Their qualifications include bachelor’s degree, diploma or certificate levels. Eligibility was based on the participants’ willingness to participate in the study. Nurses with less than six months of work experience, those who were absent during data collection time, and those who failed to give their consent to participate were supposed to get excluded from the study. Except for one participant from Orotta (not willing to participate) and one from Halibet (absent), all staff members in the EDs consented and were involved in the study. The attrition rate was 6% leaving 31 nurses who fulfilled the inclusion criteria and participated in the study.

### Data collection tools and variable measurement

The data collections tools included a self-administered questionnaire to assess triage knowledge of the nurses working in the adult emergency department and a hospital equipment checklist. The tools were adopted from a similar study conducted in Tanzania [24]. Permission was obtained from the corresponding author. The tools were in the English language and did not require translation as the language of instruction in Eritrea is English. After ensuring the face and content validity of the questionnaire through panel discussion of experts in the field, it was trialed as a pretest to evaluate the comprehension and confirm the questions were clearly understood by the study subjects.

The questionnaire included eight demographic questions and sixteen questions specifically assessing triage knowledge. Eight of these questions determined the nurse’s understanding of triage categories and interpretation of vital signs. The replies to these questions were set as “correct” or “incorrect”. The rest of the eight questions were scenario-based in which the questions asking the priority level of the mentioned scenario and the replies were set as “priority 1”, “priority 2” and “priority 3”. The nurses were required to identify and mark the correct reply. The observational check list employed practice questions and if any of the triage practices were accomplished or not were recorded in the check list as “yes” or “no”.

### Intervention

Permission to conduct the study was obtained from the ethics committee of Asmara College of Health Sciences, and the Ministry of Health at the department of research and human resource development. Further permission was obtained from the study sites prior to carrying out the study. Verbal and written informed consent was obtained from the participants before initiating the study. The researcher recruited seven trainers; an internist and a surgeon; both associate professors in the medical school, a lecturer and triage trainer in the nursing department of the medical college, a general practitioner and two triage trainers form the emergency department of the Orotta hospital, and an anesthetist with a master’s degree. Participants were trained for five days. Trainers were given different roles as per their experiences. Lectures, demonstrations and triage scenarios were prepared based on content within the Orotta triage system manual (OTS). Materials included pre-hospital (during mass casualty) and hospital triage of critically ill medical and surgical patients. Training was reviewed with the researches in detail beforehand with training including a total of 18 h of instruction. Triage flow charts were also provided to the hospitals and placed in the triage area for easy access by the nurse in charge. Data collection was undertaken at three different times; pre-training, two days post training and again three months later. Participants were asked to complete the knowledge questionnaire during their off duty time with everyone given half an hour time to finish. The observational checklist was employed in real time to document the triage practice of the study nurses. The checklist included; assessment skills, ability to prioritize and categorize patients, and documentation of the triage assessment findings. Observation was performed twice in an effort to assess the impact of training; prior to training and three months post training. Observation took place from Monday through Saturday between 07:00 -11:00 and 18:00 and 20:30, the busiest times of the emergency departments**.** Each study participant was observed once for a period of at least 120 min. To avoid duplication, a roster of all study participants was used, with each participant checked off the list following their observation. An equipment check list was also tracked to ensure the required triage equipment was present. The equipment included: thermometers, pulse oximeters, sphygmomanometers, glucometers with strips, spirometers, twelve lead electrocardiographs, arterial blood gas analyzer, and triage assessment form indicating the patient’s presenting level of severity, triage guideline/policy, and pain assessment scale.

### Data analysis

Data was entered in the SPSS (Version 22) software for analysis. Preliminary cleaning and exploratory investigations were done before conducting the main analysis. Demographic data was described using mean (SD), median (IQR), and frequency (percentage) as appropriate. The level of knowledge and skill in each of the respected questions was then obtained using the sum of the item responses converted into a percentage. Descriptive comparisons of the knowledge and practice questions at pre-intervention, immediate post, and 3 months later were done. Normality of the knowledge and practice scores was checked using Fisher’s measures of skewness and kurtosis. In order to investigate the contribution of educational intervention on the knowledge score (normally distributed) at pre, immediate post and 3 months later, one way repeated measures, ANOVA was used. Pair wise comparisons of the three time points was performed using Bonferroni post-hoc approach. Comparison of practice (not normally distributed) was done using Wilcoxon signed rank test. Tables and graphs were used to present the analyzed data. Statistical significance was considered at *p* < 0.05.

## Results

Among the participants, 71% were male. The median age of the study participants was 27 with interquartile range (IQR) of 9 with 64% of them less than 30 years old. Additional demographic and background characteristics of the study participants are shown in Table 1.

**Table 1:** Socio-demographic characteristics of the study participants (*N* = 31)

**Variable**	**Number (N)**	**Percent (%)**
Gender		
Male	22	71
Female	9	29
Age		
Less than 30	21	67
Greater than 30	10	33
Place of work		
Orotta Hospital	15	48
Halibet Hospital	16	52
Qualification		
Health Assistant	9	29
Diploma Nurse	19	61
Degree Nurse	3	10
Work experience (Median = 5, IQR = 5, Min = 1, Max = 35)		
Less than 4 years	14	45
Greater than 4 years	17	55
Experience in ED (median = 3, IQR = 5, min = 1, max = 35)		
Less than 4 years	24	77
Greater than 4 years	7	33

### Triage knowledge of nurses

#### Preliminary knowledge in triage and normal vital signs

Before the intervention, only 45% of the study participants correctly knew the definition of triage. However, the percentage able to correctly define triage increased after 2 days (90%) and three months (94%) of intervention. Correct knowledge on normal range of baseline blood pressure increased from 10% at pre-intervention to 42% at 2 days post and 52% three months later. At pre-intervention, waiting time for red, yellow and green patients were correctly identified by 36%, 29%, 19% of the participants respectively. Immediately post education, 100% of them correctly identified the waiting time to all codes, however, this knowledge declined to 52% 52% 48% respectively three months later. The percentage changes knowledge accuracy at pre-intervention, 2-days after intervention, and three months later are displayed in Table 2.

**Table 2:** Percentage distribution of participants with correct knowledge about general issues on triage

**Knowledge**	**Pre**	**2-Days**	**3-Months**
	**N (%)**	**N (%)**	**N (%)**
Triage definition	14 (45)	28 (90)	29 (94)
Normal adult BP range	3 (10)	13 (42)	16 (52)
Normal adult PR range	15 (48)	22 (71)	26 (84)
Normal adult RR range	8 (26)	14 (45)	26 (84)
Normal adult SpO_2_ range	9 (29)	25 (81)	22 (71)
Waiting time for red coded patient	11 (34)	31 (100)	16 (52)
Waiting time for Yellow coded patient	9 (29)	31 (100)	16 (52)
Waiting time for green coded patient	6 (19)	31 (100)	15 (48)

*RR* Respiratory rate, *PR* Pulse rate, *SBP* Systolic blood pressure

### Knowledge on case scenarios

The emergency case scenarios asked participants to correctly assign patients as red, yellow or green cases based on their presenting features and emergency needs. As presented in Table 3, eight case scenario were presented for the study participants to categorize. The same cases were presented on the three different study time periods: pre, immediately post training, and three months later. Nurses were most likely to correctly assign red patients and least likely to correctly assign green patients with evidence of short term improvement immediately post training, followed by an apparent erosion of that knowledge at three months. Looking at the various cases outline in Table 3, it is surprising that case 1, with marked respiratory distress and hypotension was identified as Red by 94% pre training, but only 74% three post training. Otherwise, except for the pregnant trauma patient, changes overt time were not dramatic.

**Table 3:** Percentage distribution of participants with correct knowledge about emergency case scenarios

**Case scenarios**	**Pre**	**2-days**	**3-months**
	**N (%)**	**N (%)**	**N (%)**
**Red patient (priority 1)**			
Breathing difficult, RR 8 breath/ minute SBP of 80 mmHg	29 (94)	26 (84)	23 (74)
Face burn, severe pain, RR = 29beat/min., PR = 129breath/min	15 (48)	16 (52)	24 (77)
Chest pain radiating to left arm, RR = 20 PR = 110, SBP = 90 mmHg	28 (90)	29 (94)	28 (90)
**Yellow patient (priority 2)**			
Patient with fast breathing, coughing, RR = 40 Temp 39 °C	14 (45)	17 (55)	15 (49)
Skin moderate pale, cool and dry. Reacts to voice RR = 28, PR = 110, SBP = 90 mmHg	12 (39)	11 (36)	15 (48)
**Green patient (priority 3)**			
Pregnant, walking. Trauma RR = 20, PR = 100, SBP = 130	14(45)	18(58)	10(32)
Patient on stretcher, controlled bleeding RR = 20, PR = 100, SBP = 130 mmHg	7(23)	11(36)	7(23)
Patient walking. Feeling discomfort, headache SBP = 200 mmHg	4(13)	4(13)	4(13)

The first type of case scenario were red coded patients requiring immediate medical intervention. Before the training, 94% of the nurses were able to correctly identify a red coded case who presented with an acute breathing difficulties and unstable vital signs. Correct identification decreased two days post educational intervention (84%) and even further 3-months later (74%).

There were two case scenarios representing yellow coded patients (second priority level). In the first case, the patient presented with rapid breathing, cough and high grade fever. At pre-intervention 45% of the nurses correctly identified the patient as yellow. This improved to 55% 2-days post, with a slight decrease to 49% at 3 months. Three cases scenarios represented the third priority level (green code). A pregnant ambulatory mother following minor trauma, but stable was one selected case. At pre-intervention 45% of the nurses identified her as green. This increased to 58% 2-days post, but decreased to 32% at 3-months.

### Effect of intervention through time

A one way repeated measures ANOVA (Analysis of Variance) was conducted to look the contribution of an educational intervention on the knowledge scores of the nurses over time. The average knowledge scores at time 1(pre-intervention), Time 2 (immediate post intervention) and Time 3 (three month follow up) were 6.23(SD = 2.29), 10.55 (SD = 1.79), and 9.39 (SD = 2.67) respectively. Mauchly’s test indicated that there was an evidence of sphericity (Mauchly’s W = 0.856, *p* = 0.104). Significant effect for time was observed at both univariate and multivariate ANOVA methods (Table 4).

**Table 4:** Comparison of level of knowledge of the nurses at Time 1, Time 2, and Time 3

**Time Period**	**N**	**Mean (SD)**	**Test of Sphericity**	**Wilk's Lambda**	**RANOVA *p*-value**
Time 1 (Pre-intervention	31	6.23(2.29)	Mauchly’s W = 0.856 *p* = 0.104	Wilk’s λ = 0.275 F(2,29) = 38.32 *p* < 0.0001 partial eta squared = 0.725	< 0.0001
Time 2 (Post intervention)	31	10.55(1.79)			
Time 3 (3 months follow-up)	31	9.39(2.67)			

### Practice of nurses on triage

#### Airway and respiratory system assessment

In order to assess the practice of nurses on triage, observation was done on the vital body conditions including respiratory and airway assessment, circulatory status assessment, as well as temperature and pain assessments (Table 5).

**Table 5:** Percentage distribution of participants regarding practice on airway and respiratory system, circulatory status temperature, and pain assessment

**Practice**	**Pre**	**3 months later**
**Airway & Respiratory system assessment**		
Airway assessment	15(100)	12(80)
Respiratory status assessment	3(20)	5(33)
Look for chest movement	2(13)	3 (20)
Listen for breathing sound	2(13)	3(20)
Breathing pattern	3(20)	5(33)
**Circulatory status assessment**		
Blood pressure	15(100)	15(100)
Pulse rate	15(100)	11(73)
Skin/ mucous color (pink, pale)	1(7)	1 (7)
Skin temperature (warm, hot, cool/ cold)	1(7)	1(7)
Capillary refill time	1(7)	1(7)
**Temperature & pain assessment**		
Temperature assessment	0 (0)	1 (7)
Pain assessment	0 (0)	6(40)
Neurological assessment	1(7)	8(53)
Triage documentation	0(0)	13 (87)
Correct reassessment way on regular bases	1(7)	6(40)

Observation were undertaken at two time points only: at pre-intervention and post-intervention 3 months follow-up. Regarding respiratory and airway assessment, it was generally observed that most of the nurses were performing a thorough check, other than ensuring airway patency. The percentage of nurses assessing blood pressure and pulse rate was 100%, but weak in assessing perfusion parameters. Appropriate pain and temperature assessments were not done at all during the pre-intervention time and showed some improvement post intervention from 7 to 40% in three months. A triage documentation rate of 0% showed a dramatic improvement to 87% at three months follow up. Observations also of triage documentation showed a dramatic improvement (87%) in the three months follow up. Observations also looked to determine whether triage nurses regularly reassessed their patients. This was an area of weakness with a rate of 1% pre-intervention, 1% post-innervation, and 6% 3 months post intervention.

Wilcoxon signed rank test was used to compare the score in appropriate practice on triage before and after the intervention. The median score of appropriate triage practice before intervention (Md = 6, IQR = 3) was not significantly different (*p* = 0.053) from the practice after three months (Md = 8, IQR = 5) of intervention (Table 6).

**Table 6:** Comparison of skill before and after intervention (*N* = 15)

**Time Period**	**N**	**Median (IQR)**	**Wilcoxon signed rank value**	***P*-value**
Pre-intervention	15	6(3)	73	0.053
3-month follow up	15	8(5)		

## Discussion

The role of the triage nurse has emerged in response to growing community demand for a more accessible and efficient ED services [25]. According to data from the study hospitals, the prevalence of accidents and medical problems and subsequent emergency visits have shown a steady increase. To better manage these increased demands, ED nurses should receive ongoing training and educational renewal to be optimally prepared to identify and collaborate in treating the acutely and critically ill patients. Worldwide, many emergency nurses receive comprehensive, evidence-based triage education and a clinical orientation with an experienced preceptor to enhance their triage knowledge and skills [22]. The lack of in-service training has been one of the great challenges in our settings. In this current study, the majority of the ED nurses (84%) had never received any emergency and critical care related in-service training and as a reflection, more than half (55%) were unable to define triage before the training was provided. Comparable results were reported in a similar study form Tanzania [24]. A study from a neighboring country also reported similar results in which 56% of the emergency nurses were unable to define triage [26]. In this current study, being able to define triage improved dramatically to 90% during the immediate post intervention period and 94% within the three months follow-up. These results followed a comprehensive lecture based and practical triage demonstration during the 18-h training sessions. Charts and a booklet based on OTS were also disseminated in the study hospitals. The average knowledge scores prior intervention, immediate post intervention and after three months follow-up were 6.23 (SD = 2.29), 10.55 (SD = 1.79), and 9.39 (SD = 2.67) respectively. Similar results were reported from other studies [18, 24, 26, 27].

Emergency department nurses are required to demonstrate situational awareness and apply their triage knowledge according to their professional standards [22]. They must be able to assess patient acuity and quickly prioritize them based on anticipated emergency requirements [28]. Seconds and minutes can be vital for saving patient lives. Pre study less than 35% of the nurses were able accurately define the waiting time for red, yellow, and green coded cases. Timeliness is considered as a key component in quality of ED care and delays can negatively influence patient outcomes [29]. Rapid and accurate triage of patients is one key to quality patient care. Accurate triage of injured patients has reduced fatalities and improved resource usage [7]. Many triage education programs are underpinned by the assumption that knowledge acquisition will result in improved triage decisions. Factual knowledge is an important factor in improving triage decisions and ultimately enhancing patient outcomes [1]. The post training knowledge of the nurses in this study showed a level of erosion at the three months follow-up time. This suggest that ongoing educational enhancement, supervision and review are all important components of quality assurance programs. Several studies have examined the issues of degradation of knowledge and skills over time [30]. To lessen the impact ongoing education and refresher course are important [31]. Given the importance of timely interventions for patients in need, quick and accurate decision making is crucial to determine which patients need immediate care and who can safely wait [32]. By correctly identifying presenting patient conditions and initiating necessary and appropriate interventions timely, triage nurses serve as the eyes and ears of the acute care system [19]. In this study, the nurses were asked to identify and give first priority for those patients who need immediate care. Initially, less than 50% assigned the correct triage category. This increased to 77% post intervention. A better understanding of the relationships between clinical decisions, knowledge, and experience is pivotal for the rigorous evaluation of education programs [1].

### Practice

A participatory observation of practice performance was performed in the ED of Orotta Hospital. Despite the training given and the knowledge improvement, nursing practice remained deficient and triage inaccuracy was observed. A similar study from Brazil reported consistent results in which 17% of the admitted patients were triaged inaccurately [33]. All of the nurses failed to check the airways of unconscious patients for patency or airway obstruction despite its importance. None were seen to measure the respiratory rate. Despite their knowledge of normal vital sign ranges, the practice of vital sign measurement was poor. Similar reports were reported by Aloyce et al. [24]. Respiratory rate is a good indicator of serious respiratory illness and helps in discriminating between stable patients and those who at risk, one indicator of triage acuity [34]. In our study, the nurses were observed to rely on reading of the pulse oximetry to determine the respiratory status of the patient. Studies have shown that pulse oximetry measurement is not be a replacement for respiratory rate measurement as it is not an indicator of a number of serious illnesses [35]. Three months post training there was no significant improvement in their practice. The reasons given by the nurses included patient overcrowding, too few nurses, and lack of necessary triage equipment were the major barriers to improved standards of practice. Lack of basic equipment for assessment has been reported as a factor that contributes to triaging delays [25]. Similarly, a study from Pakistan reported that, most healthcare providers were inadequately equipped to treat emergencies [36]. Another study from Pakistan stated that their nursing curriculum for different nursing programs lacked sufficient triage content to prepare nurses for the role of emergency triage [19]. A study carried out by Abd-Hamid, (2011) to determine the level of nurses' knowledge and practice regarding triage in emergency care in Ismailia university hospital, challenged the notion that education was the problem, reporting that triage training courses didn’t show a significant effect on nurses' practice [37].

In this study, the emergency nurses were observed to routinely measure blood pressure. They also generally assessed arterial oxygen saturation using the pulse oximetry, however, when pulse oximetry was unavailable, they didn’t count pulse rate and appeared unable to determine pulse status of patients, indicating a lack of assessment skill. Triage assessment should not only be done in the triage rooms, but should be an on-going process involving careful observation and re-assessment when indicated [1]. Pre study after triaging, only one nurse (7%) was seen to do regular re-assessments. This increased to 40% post-intervention. Poor performance of triage documentation was observed to increase dramatically post training improving from 0 to 87%.

### Limitations

The variation in the triage practice among the reviewed articles could be a source of bias on the results. Since all the nurses of the emergency room was included in the study, and being the only national referral hospital, the workload could have affected the workshop demonstrations on how to perform standard patient triage. The triage training was conducted only in the national referral hospitals found in the capital city, thus the results may only be generalizable to these hospitals and cannot be generalized to smaller and nonurban locations.

## Conclusion

The study concluded that, the level of knowledge and practice of triage among nurses of the ED was low at pre-training. The training provided an initial improvement in knowledge, without significant improvement in triage nursing performance. To optimize ED triage performance, appropriate, timely in-service training including skills workshops to teach triage assessment and documentation standards are required to ensure new staff are appropriately trained and experienced staff have their knowledge and skills refreshed.

## Supplementary Information

**Additional file 1.**

**Additional file 2.**

**Additional file 3.**

**Additional file 4.**

**Additional file 5.**

**Additional file 6.**

**Additional file7.**

**Additional file 8.**

## Data Availability

All data generated or analyzed during this study are included in this published article and as supplementary files.

## Abbreviations

ANOVA: Analysis of Variance
CI: Confidence Interval
ED: Emergency Department
ENA: Emergency Nurse Association of United States of America
IQR: Interquartile Range
MoHE: Ministry of Health Eritrea
NCHE: National Commission for Higher Education
OTS: Orotta Triage System
SD: Standard Deviation
SPSS: Statistical Package for Social Sciences
