# Supplementary material for: Effect of triage training on the knowledge application and practice improvement among the practicing nurses of the emergency departments of the National Referral Hospitals, 2018; a pre-post study in Asmara, Eritrea
Source: BMC Emerg Med. 2022 Dec 2;22:190. doi: 10.1186/s12873-022-00755-w (PMC9719223; doi:10.1186/s12873-022-00755-w)
Supplement: Supplementary file 1 — Additional file 1. [file 12873_2022_755_MOESM1_ESM.pdf]

OROTTA TRIAGE SYSTEM DEVELOPMENT AND IMPLEMENTATION

Introduction

Everyday emergency departments face with large numbers of patients suffering from a wide range of problems. The workload varies from day to day and from hour to hour and depends on the numbers of patients attending and what is wrong with them. It is absolutely essential that there is a system in place to ensure that these patients are seen in order of clinical need rather tan in order of attendance. In the past year great steps have been made towards establishing Triage System in Europe; this follows on from similar work in Australia and Canada. Triage is a system of clinical risk management employed in Emergency Departments worldwide to manage patient flow safely when clinical need exceeds capacity. Systems are intended to ensure care is defined according to patient need and in a timely manner. This Triage system was developed by Italian Healthcare Team (Azienda Ospedaliera Universitaria Careggi – Florence – Italy) during the international cooperation project named "Firenze-Asmara Km zero" in collaboration with Eritrean Ministry of Health and with medical and nursing staff of Orotta National Referral Hospital (Asmara – Er). The Orotta Triage system was derived from the Manchester Triage System © (Last issue in 2005). The Manchester Triage Group was set up in November 1994 with the aim of establishing consensus amongst senior emergency physicians and emergency nurses about triage standards and now is one of worldwide best triage system. The activation of the triage function appears crucial to guarantee a suitable safety level for the patients and a correct ED functioning (currently the patients are managed in the examination and treatment rooms without a prior evaluation of the clinical conditions or priority of access with consequent danger for the patients that are visited according the order of arrival or however casually). It seems reasonable that the triage process should include the Triage Out activity for patients that don't need urgent care and that can be sent safely to the Out-patient Department (OPD). It is currently assumed that the use of 4 priority levels triage (the 5 levels systems are shown more effective for ED with more than 100 daily accesses) is safe and effective in accord with the international literature and with a previous draft paper issued by the healthcare team of the Italian Cooperation. The levels of priority are defined as follows:

- Red code: Emergency (compromised or absent vital functions) immediate access to care
- Yellow code: urgency (potential life threatening condition or serious damage for the health state due to alteration of the vital parameters or clinical presentation) fast access to care
- Green code: acute problem (No potential life threatening condition or risk of serious damage for the health state) delayed access to care after red and yellow patients
- White code: non acute problem without any risk for life or for patient's health (improper access). No access to ED care (send patient to OPD when operational) or delayed access to care.

Each of the triage categories was given a number, a colour and a name and was defined in terms of ideal maximum time to first contact with the treating clinician.

NUMBER	NAME	COLOUR	MAX TIME (Minutes)
1	Immediate	Red	0
2	urgent	Yellow	10 - 30
3	Standard	Green	60 - 120
4	Non Urgent	White	120 - 240

As practice has developed over the past 10 years triage scales have been established around the world. The target times themselves are locally set, being influenced by politics as much as medicine particularly at lower priorities, but the concept of varying clinical priority remains current.

THE OROTTA TRIAGE SYSTEM PROJECT

During September 2006 a review and an adaptation to the local needs of the Manchester Triage System © was performed. At the end of September will begin an educational programme for whole emergency department nursing staff of Orotta hospital. In October 2006 will start the Triage activity at Orotta Emergency Department with clinical supervision and tutorship performed by Italian healthcare team. The Eritrean nursing staff deal with a large variety of forms and papers and for this reason appear reasonable the use of clinical card (green card) also for the triage purpose without the introduction of a new triage evaluation sheet. The project provides the arrangement of triage area and triage station. After analysis and strict collaboration with ED Head Nurse the triage area should be arranged as shown in figure:

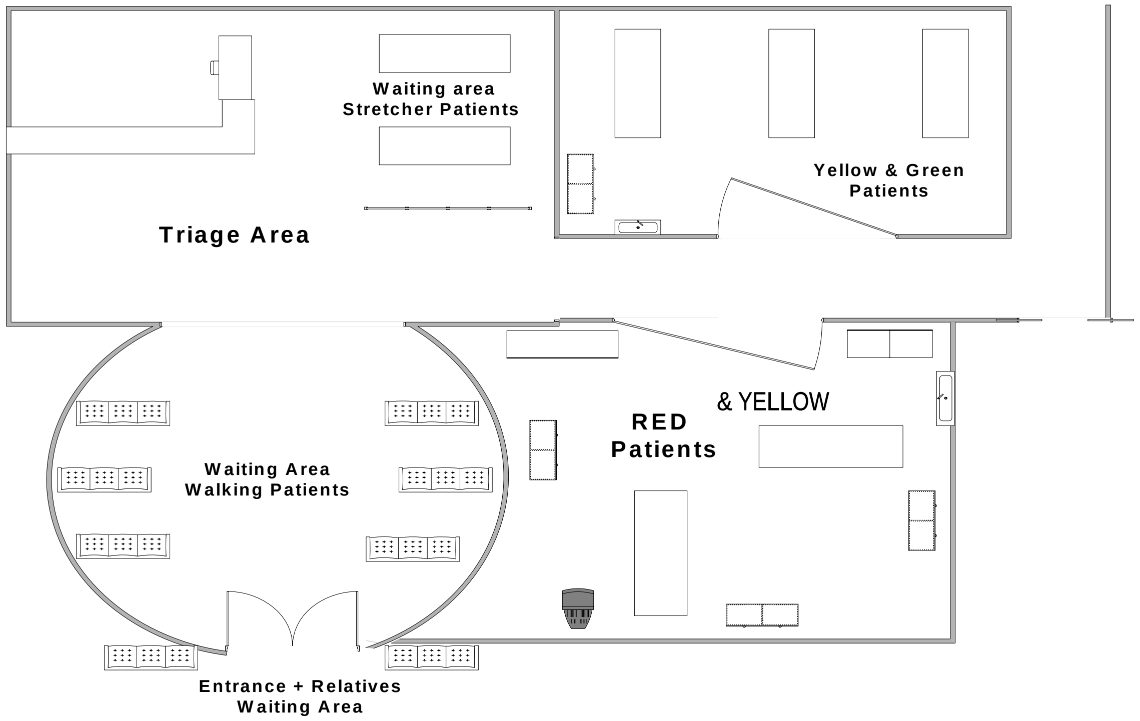

The triage station will be set near the desk in the triage area and will include a **triage cart** with the following materials:

- Sphygmomanometer
- Stethoscope
- Pulse oxymeter
- Random blood glucose tester
- Thermometer
- Box with sterile gauzes
- Gloves
- Plaster
- Disinfectant

Due to the activation of triage function seems to be necessary also a redesign of job organisation and a shared way to use the ED rooms. Currently the ED work schedule encompass at least the presence of 3 nurses (1 RN and 2 AN) in the morning shift (h 08 - 16), in the afternoon shift (h 16 - 20) and in the night shift (h 20 - 08) with a

maximum of 5 nurses in the morning shift according to availability. Actually the nurses don't have particular assignments (everyone looks after everything).

Proposed job organisation for Orotta Hospital ED nursing staff

3 Nurses model		4 Nurses model		5 Nurses model	
Nurse	Assignment	Nurse	Assignment	Nurse	Assignment
A	Triage & Observation area	A	Triage	A	Triage
B	Procedure room	B	Procedure room	B	Procedure room
C	Resuscitation Room	C	Resuscitation Room	C	Resuscitation Room
		D	Observation beds	D	Observation beds
				E	“Flying” Nurse

The proposed job organisation is based on the actual patients flow and is subject to further evaluation and adjustments. Moreover the job organisation must be flexible (i.e. if a new critical patient arrive in resuscitation room one of another nurses help the resuscitation room nurse for initial stabilisation). Actually the patients flow don’t seems too high and is possible to bring them in the examination and treatment room without long waiting time but in the future, when the Orotta Hospital will became fully operational, it will be necessary to use appropriately the ED Rooms. The resuscitation room and the procedure room must be used for initial patient assessment and management and, after that, the patients that don’t needs instrumental monitoring or life support interventions must be moved in the observation rooms waiting the end of diagnostic and therapeutic process. The triage activity at Orotta ED will be monitored for three months for verification of appropriateness of triage presentation charts and triage nurses skills. The following standards, measured in a random sample of triage evaluations, help in appraising the triage system and in suggesting some change and olso for verification of achievement of learning process:

- 0% episode incomplete
- 95 % accuracy
- 95 % agreement between triage nurses and tutor
- Max 15% overestimation
- Max 5% underestimation

TRIAGE METHODOLOGY

In general terms a triage method can try and provide the practitioner with the diagnosis, with the disposal, or with a clinical priority. The Manchester Triage Group quickly decided that the triage methodology should be designed to allocate a clinical priority. This decision was based on three major tenets - first the aim of the triage process in an Emergency Department is to aid both clinical management of the individual patient and departmental management; this is best achieved by accurate allocation of a clinical priority. Second the length of the triage process is such that any attempts to accurately diagnose a patient are doomed to failure. Finally it is apparent that diagnosis is not accurately linked to clinical priority, the latter reflects a number of aspects of the particular patient’s presentation as well as the diagnosis; for example a patient with a final diagnosis of ankle sprain may present with severe, moderate or no pain, and their clinical priority must reflect this. In outline the triage method requires practitioners to select from a range of presentations, and then to seek a limited number of signs and symptoms at each level of clinical priority. The signs and symptoms that discriminate between the clinical priorities are termed *discriminators* and they are set out in the form of flow charts for each presentation - the *presentational flow charts*. Discriminators that indicate higher levels of priority are sought first, and to a large degree patients who are allocated to the standard clinical priority are selected by default.

Priority and Management

It is easy to become confused between the clinical priority and the clinical management of a patient. The former requires that enough information is gathered to enable the patient to be placed into one of the four defined categories as discussed above; the latter may well require a much deeper understanding of the patients needs, and may be affected by a large number of extraneous factors such as time of day, the state of the staffing and the number of beds available. Furthermore the availability of services for particular patients will fundamentally affect individual patient flow. Separately staffed "streams" of care for particular patient groups will run at different rates. This does not affect underlying clinical priority which affects the order of care within rather than between streams in such a system. Triage is a fundamental part of clinical risk management in all departments when clinical load exceeds clinical availability. Emergency Triage promulgates a system that delivers a teachable, auditable method of assigning clinical priority in Emergency settings. It is not designed to judge whether patients are appropriately in the Emergency setting, but to ensure that those that need care receive it appropriately quickly. It can be used to monitor care and to signpost streams of care – these will be determined by local provision and actual availability.

The decision making process and triage

Decision making is an essential and integral part of medical and nursing practice. Sound clinical judgement about patient care requires both thought and intuition, and both of these must be based on professional knowledge and skill. Many practitioners argue that critical decision making is only about 'common sense' and 'problem solving', and to a certain extent they are correct. It is, however, more than this and requires a certain level of skill. Within the decision making process clinicians are expected to:

- Interpret
- Discriminate
- Evaluate

the information they gather about patients, and critically appraise their actions following that decision. Without a framework of reference on which to base these decisions, they will be unstructured, chaotic and potentially unsafe. The ability to make sound decisions is essential for good patient care.

DECISION MAKING DURING TRIAGE

Despite all the theories, decision making is quite simply a series of steps to reach a conclusion and consists of three main phases - identification of a problem, determination of the alternatives and selection of the most appropriate alternative. An approach to making critical decisions has been described which uses the following five steps.

- Identify the problem
- Gather and analyse information related to the solution
- Evaluate all the alternatives and select one for implementation
- Implement the selected alternative
- Monitor the implementation and evaluate outcomes

This approach incorporates a number of theories and methods.

CHANGING CURRENT DECISION MAKING PRACTICE

For many experienced nurses the introduction of a new framework for triage decisions poses some problems. It is difficult to unlearn individual methods of decision making which have developed over years of practice. However this change should be viewed as a further refinement of their present system, providing for the first time a clear rational for their decisions and an auditable system. This systematic approach will be a major contribution to the body of knowledge when used to teach junior staff, who rely so heavily on experts to inform and guide their own practice. The actual process of triage decision making presented in this manual is effective and adaptable to any practice setting, and has value to nurses irrespective of their level of experience.

The Triage Method

The Triage method has been designed to allow the Triage Practitioner to rapidly assign a clinical priority to each patient. The system selects patients with the highest priority first and works without making any assumptions about diagnosis; this is deliberate, and recognises that Emergency Departments are to a large extent driven by the patients presenting signs and symptoms.

IDENTIFYING THE PROBLEM

Clinical practice is geared around the concept of *presenting complaint* - that is the chief sign or symptom identified by the patient or carer. A list of presentations pertinent to triage is shown below:

Abdominal pain in adults	Ear problems	Pregnancy
Abdominal pain in children	Exposure to chemicals	PV bleeding
Abscesses and local infections	Eye problems	Rashes
Allergy	Facial problems	Seizure
Apparently drunk	Falls	Self-harm
Assault	Foreign body	Sexually acquired infection
Asthma	GI bleeding	Shortness of breath in adults
Back pain	Head injury	Shortness of breath in children
Behaving strangely	Headache	Sore throat
Bites and stings	Irritable child	Testicular pain
Burns and scalds	Limb problems	Torso injury
Chest pain	Limping child	Unwell adult
Collapsed adult	Major trauma	Unwell child
Crying baby	Mental illness	Urinary problems
Dental problems	Neck pain	Worried parent
Diabetes	Overdose and poisoning	Wounds
Diarrhoea and vomiting	Palpitations	

The first part of the triage method requires the practitioner to select an appropriate presentation from the list. By selecting the appropriate presentation the practitioner is led to a *presentational flow chart;* the chart identifies discriminators which allow the clinical priority to be determined. Great care has been taken to ensure that the charts are consistent in their approach, since it is recognised that a number of patients chief complaints may lead to more than one presentational flow chart. Thus a patient who is feeling unwell with a stiff neck and a headache will be given the same priority whether the practitioner uses the *Unwell Adult, Neck Pain* or *Headache* flow charts.

GATHERING AND ANALYSING INFORMATION

To a large degree the patients' presentation will dictate which presentational flow diagram is selected. Following this selection information must be gathered and analysed to allow the actual priority to be determined. The flow diagram structures this process by showing key discriminators at each level of priority - the assessment is carried out by finding the highest level at which the answer posed by the discriminator question is positive. Discriminators are deliberately posed as questions to facilitate the process.

Discriminators

Discriminators, as their name implies, are factors that discriminate between patients such that they allow them to be allocated to one of the four clinical priorities. They can be *general* or *specific.* The former apply to all patients irrespective of their presentation and therefore appear time and time again throughout the charts; on each

occasion the general discriminators will lead the Triage Practitioner to allocate the same clinical priority. Specific discriminators are applicable to individual presentations or to small groups of presentations, and tend to relate to key features of particular conditions. Thus while *severe pain* is a general discriminator, *cardiac pain* and *pleuritic pain* are specific discriminators. General discriminators appear in many more charts than specific ones. All the discriminators used are defined in the discriminator dictionary at the end of this manual, and the definitions of the specific ones in use on individual charts are repeated on the accompanying chart notes or ease of reference. All the specific discriminators have been reviewed. Key additions include *acute neurological deficit* and *significant respiratory history* which are designed to ensure that patients with stroke and unstable COPD respectively receive early assessment and investigation. General discriminators are a recurring feature of the charts, and a proper understanding of them is essential to an understanding of the triage method. Six general discriminators are discussed further here - these are shown in the box.

- Life threat
- Haemorrhage
- Pain
- Conscious level
- Temperature
- Acuteness

Life threat

To a practising Emergency Nurse or Emergency Physician *Life threat* is perhaps the most obvious general discriminator of all. Broadly speaking this recognises that any cessation or threat to the vital (ABC) functions places the patient in the first priority group. Patients are unable to maintain their own airway for any length of time have an insecure airway. Additionally patients with stridor have significant airway threat - this may be an inspiratory or expiratory noise, or both. Stridor is heard best on breathing with the mouth open. Absence of breathing is defined as no respiration or respiratory effort as assessed by looking, listening and feeling for 10 seconds. Inadequacy is a more difficult concept - but in general patients who are failing to breathe well enough to maintain adequate oxygenation have inadequate breathing. There may be a increased work of breathing, signs of inadequate breathing, or exhaustion. Absence of pulses is only diagnosed after palpation over a central pulse for 10 seconds. Shock can be difficult to diagnose - the classical signs include sweating, pallor, tachycardia, hypotension and reduced conscious level.

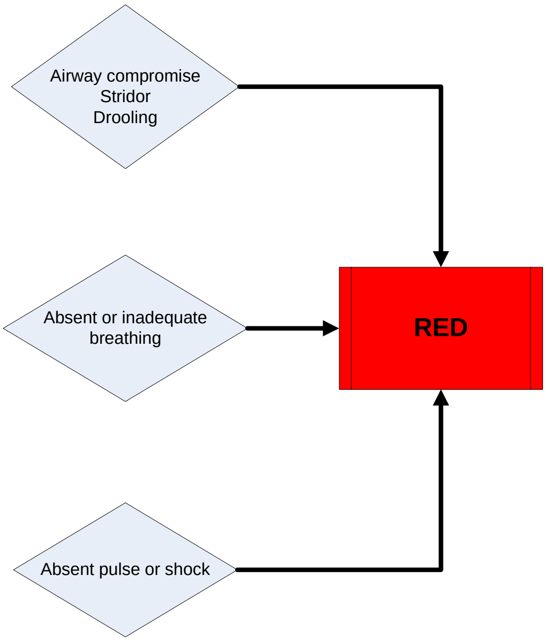

	RED	YELLOW	GREEN
AIRWAY	Airway compromise		
BREATHING	Inadequate breathing<br>Very low SaO2	Acutely SoB<br>Low SaO2	
CIRCULATION	Shock<br>Exsanguinating haemorrhage<br>Uncontrollable major haemorrhage	Abnormal pulse<br>Marked tachycardia<br><br>Uncontrollable minor haemorrhage	

DISABILITY	Unresponsive child<br>Currently fitting	Altered conscious	

Pain

From the patients perspective pain is a major factor in determining priority. The use of pain as a general discriminator throughout the presentational flow charts recognises this fact and implies that every triage assessment should include an assessment of pain. Pain assessment is dealt with in detail in a next section and readers are referred there for detailed discussion; in general terms the discriminator severe pain is intended to imply pain that is unbearable - often described as the worst ever, while moderate pain refers to pain that is bearable but intense. Any patient with any lesser degree of pain should, if no other discriminators suggest a higher categorisation, be allocated the standard rather than the non-urgent priority. The general pain discriminator describes the intensity or severity of pain only. Other characteristics of pain, such as site, radiation and periodicity may feature as specific discriminators in particular presentational flow charts.

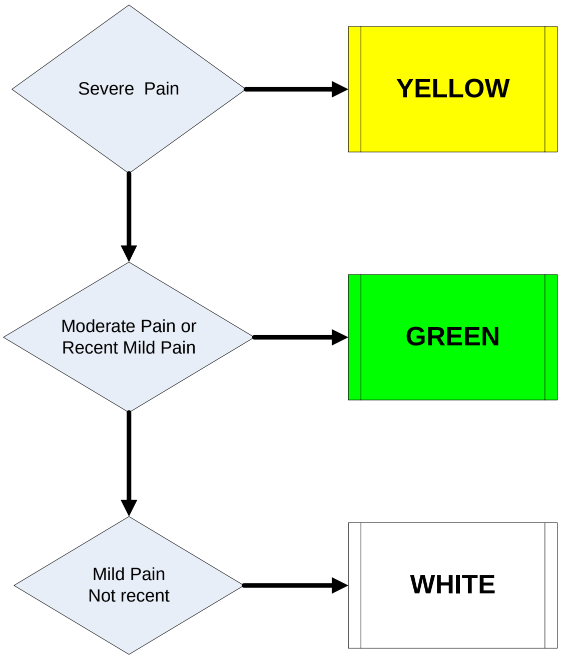

Hemorrhage

Haemorrhage is a feature of many presentations - particularly, but not exclusively, those involving trauma. The haemorrhage discriminators are exsanguinating, uncontrolled major, or uncontrolled minor. The use of the success of attempts to control the haemorrhage is deliberate since, in general, continuing bleeding has a higher clinical priority. While of course in practice it can be difficult to decide which category a particular haemorrhage falls into, the definitions of the discriminators are designed to help the practitioner do this. Exsanguinating haemorrhage is present if death will ensue rapidly unless bleeding is stopped. A haemorrhage that is not rapidly controlled by the application of sustained direct pressure, and which continues to bleed heavily or soak through large dressings quickly is described as a uncontrolled major haemorrhage, while bleeding that continues to bleed slightly or ooze is described as uncontrolled minor haemorrhage. Any bleeding, however modest, will - unless another discriminator leads to allocation of a higher clinical priority - be allocated to the standard priority.

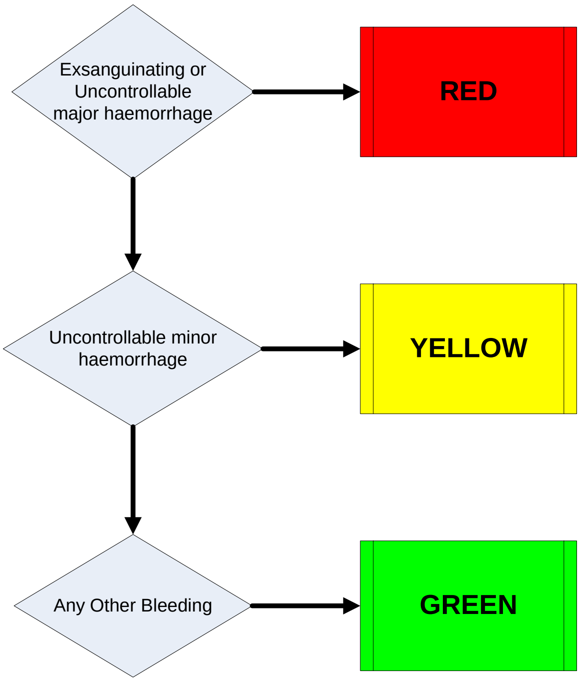

Conscious level

Conscious level is considered separately in adults and children. In adults only currently fitting patients are always be categorised immediate, whilst all unresponsive child are placed in this clinical priority. Adults patients with altered conscious level (responding to voice or pain or unresponsive) are categorised as urgent as are children who respond to voice or pain only. All patients with a history of

unconsciousness should be allocated to the Standard category. The fact that all patients with alterations in conscious level are allocated to the urgent priority may be at odds with current practice; this is especially so with regard to the clinical priority given to patients that are intoxicated or under the influence of drugs. Two points need to be made about this: First the aetiology of alterations in level of consciousness is largely irrelevant to determining the risk to the patient - an altered conscious level due to drugs or alcohol is clinically as important as altered conscious level due to other causes. Secondly most drunk patients do not have an altered level of consciousness. Specific points about the allocation of clinical priority to patients who are apparently drunk are dealt with in the presentational flow chart of that name.

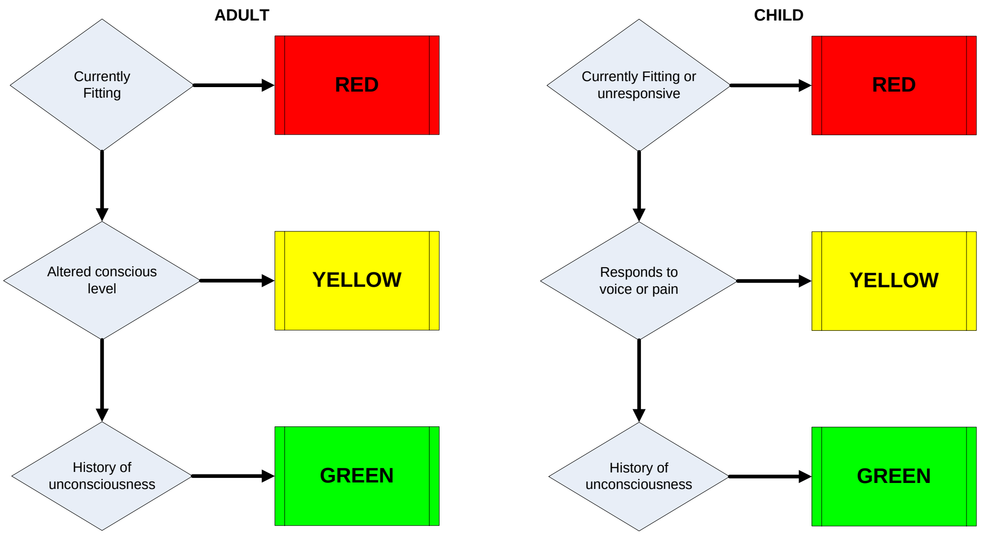

Temperature

Temperature is used as a general discriminators. It may be difficult to obtain an accurate measurement during the triage process, although rapid reading tympanic membrane thermometers make this more attainable; clinical impression of skin temperature followed as soon as possible by an accurate assessment of core temperature is an alternative approach. If the skin feels very hot the patient is clinically said to be very hot - this corresponds with a temperature > 40°C, similarly if the skin feels hot the patient is clinically said to be hot and this corresponds with a temperature > 38.5°C. A patient with warm skin fulfils the discriminator of warmth and this goes with a temperature > 37.5°C. Patients with cold skin can be said to be clinically cold - a core temperature of < 35°C matches this. A very hot adult and hot child will always be categorised as urgent, whilst a hot adult will be categorised as standard. Patients who are cold (whatever their age) will be allocated to the urgent priority.

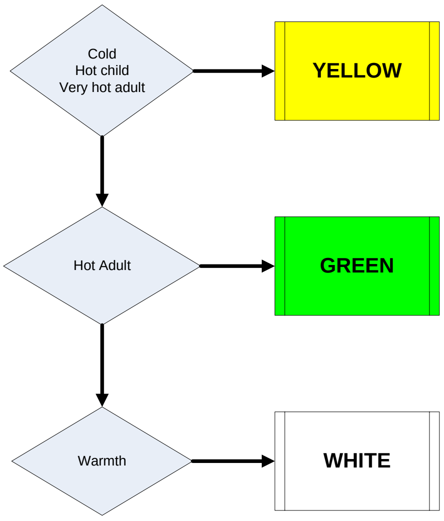

Acuteness

Within this text certain conventions have been used to help with consistency. The term abrupt is used to indicate onset within seconds or minutes, rapid to indicate less than 12 hours and acute to indicate a time period of 12-24 hours. Recent symptoms and signs are those that have appeared within the past 7 days. Whilst most clinicians have no problem accepting that the accounting of onset can help indicate the clinical priority, it is slightly more controversial to argue that chronicity (in this case greater than 7 days) is used to define a non-urgent problem. However on reflection it is intuitive that the relatively long time that the problem has been present indicates that the

patient can be allocated the non-urgent priority (ie to wait up to 4 hours) without clinical risk. The triage method is such that the presence of any other general or specific discriminators relevant to the presentation will result in allocation of a higher priority. The use of this discriminator is not intended to "punish" patients for turning up "inappropriately", nor it is intended to ensure that patients who have had injuries or illnesses for a long time have extended waiting times. The actual waiting time for patients with stable problems not of recent onset will depend on the current case mix and case load of the department, and the resources available.

Secondary triage

It may not be possible to carry out all the assessments necessary at the initial triage encounter - this is particularly so if the workload of the department is high. In such circumstances the necessary assessments should still be carried out, but as secondary procedures by a receiving nurse. More time consuming assessments (such as blood glucose estimation) are often left to the secondary stage. Many charts have a ‘risk limit’ placed on them. This indicates the lowest priority that can be applied to the patient if all observations needed are not complete.

EVALUATING ALTERNATIVES AND SELECTING ONE

Selection of a presentational flow chart leads to the selection of a number of general and specific discriminators which can then be tested against the patient. The skill in implementing the triage method presented here lies in the evaluation of this testing. Practitioners must decide whether the criteria for the presence of certain discriminators are fulfilled, and must decide which of the discriminators that are present lead to the highest clinical priority.

IMPLEMENTING THE SELECTED ALTERNATIVE

This step is essentially a procedural one. The inevitable outcome of the information gathering, analysis and evaluation discussed above will be the decision that a particular discriminator (general or specific) is the highest positive one. This leads to allocation of one of the clinical priorities. Implementation involves recording the allocated priority. The triage method outlined here allows documentation to be very simple and precise. All that is required is a record of which presentation chart is being used, which discriminator defines the category and which category has been selected. Thus, for instance, the triage record of a patient with chest pain might be as shown in the box. This simple approach to documentation not only allows for simple audit but also means that the reasons for the decision are quite overt.

Chest Pain ⇨ Pleuritic pain ⇨ Standard (Green)

MONITORING AND EVALUATING

Clinical priority can change and triage must therefore be dynamic. The triage method described here can be carried out rapidly and reliably by trained staff - it is therefore useful as a tool for multiple re-evaluations of clinical priority during the patients stay. Every nursing encounter can be used as a triage assessment, and any change in clinical priority can be rapidly notified and acted upon.

Pain assessment as part of the triage process

INTRODUCTION

Little information is available about the pain experienced by patients presenting to the Emergency Department, but that that is suggests that pain is a key issue for patients, that staff assess pain poorly and that pain management is suboptimal (oligoanalgesia). Pain is therefore an important issue for a number of reasons :

- The majority of patients attending Emergency Departments have some degree of pain
- The amount of pain influences the urgency
- The adequacy of pain management is a key criteria for patient satisfaction
- Patients in pain can become agitated and aggressive
- Patients in pain are a source of distress and stress to both staff and other patients
- Patients have an expectation that their pain will be dealt with

There are a number of advantages in assessing pain as part of the triage process. First it ensures that a patients pain is managed at the earliest opportunity – if the patient is made comfortable it may be possible to recategorise them to a lower level of priority; this allows staff to be released to treat patients who need to be seen urgently for other reasons. Patient anxiety is reduced and communication is improved. Without pain assessment the provision of analgesia type at triage is not possible.

The pain assessment process at triage

Pain assessment is an integral part of the triage methodology. This was a deliberate and explicit recognition of the importance of pain for the reasons discussed above. It is recognised that this resulted in quite a few patients being categorised into a higher priority than was traditionally the case: this was a deliberate attempt to change poor practice. If a patients pain is to be assessed formally at triage, and the outcome of that assessment is to help determine the urgency with which that patient is to be seen, then all triage practitioners must be competent in assessing pain - and the pain assessment must be valid and reproducible. It is unrealistic to expect that only the patients subjective assessment will be taken in to consideration during this process. By the same token it is inappropriate that the triage nurse makes their own subjective assessment of the patients pain in isolation.

Pain assessment in the Emergency Department

This can be difficult because patients may feel under pressure to say that their pain is severe so as to justify their attendance, and some patients, particularly children, may deny that they have pain to avoid having treatment or being admitted to hospital. Some practitioners assessment and management of pain may be influenced by 'traditional' pathways of care. For example patients who have fractures are offered immediate analgesia, but patients with abdominal pain may not be offered analgesia until the surgeons have seen them. There may be concerns that a patient will score pain higher if it is thought that this will result in quicker treatment

PAIN ASSESSMENT TOOLS

Many Emergency Departments now use a formal pain assessment tool, but many such tools suffer from the fact that they were developed for use with post-operative and chronically ill patients.

There are three main types of pain assessment tools:

- Verbal descriptor scales
- Visual analogue scales
- Pain behaviour tools

Verbal descriptor scales

These scales consist of a number of word descriptors, usually three or five, which are numerically ranked. The most common descriptors are

Descriptors	Numerical value
none	2
slight	4
moderate	6
severe	8
agonising	10

and the numerical value increases with the severity of the pain. The verbal descriptor scale is short and relatively easy for the patient to use and has been employed in the Emergency department environment.

Advantages	Disadvantages
It provides a score which is easy for the nurse to analyse	The use of a single word from an limited list may not reflect the pain that the patient is experiencing

It probably produces reliable data	It is not suitable for patients with speaking or language comprehension problems
It can be modified for use with children	It is the patients subjective assessment

Visual Analogue Scales

These scales usually consist of a straight line representing varying levels of pain with verbal anchors at each end.

NO PAIN **PAIN AS BAD AS IT COULD BE**

Patients can mark anywhere on the line. Verbal or numerical (Numerical Rating Scale) descriptors can also be added beneath the line in addition to the word anchors. The line can also be broken down to facilitate scoring for evaluation or comparative purposes.

NO PAIN

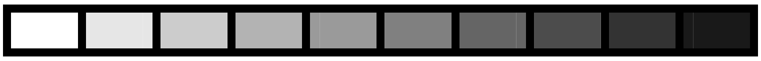

1 2 3 4 5 6 7 8 9 10

PAIN AS BAD AS IT COULD BE

Advantages	Disadvantages
Easy and fast to use and score	Some patients choose to mark the line near one of the verbal anchors
These scales may be more sensitive than Verbal descriptors	Certain patients find VAS too abstract to use in particular those in severe pain, those with lower educational abilities or those with impaired motor co-ordination. Elderly people have some difficulty in using these scales.
If used correctly they are reproducible and reliable	

Pain Behaviour Tools

These tools have been developed relying on the principle that patients who are in pain exhibit certain behaviours and physiological changes. These tools can measure:

- verbal response
- body language
- facial expression
- behavioural changes
- conscious level
- physiological
- changes

A number of different tools exist, each based on combining a number of the above factors.

Advantages	Disadvantages
Can be used in patients with communication problems.	Complex scales. Comparison and scoring is difficult.
	The patients subjective assessment is not included.
	Difficult to ensure that pain alone underlies the observed changes.
	Time consuming, taking 5 - 15 minutes to use.

The Pain Ruler

No single pain assessment tool is better than another, although some would seem to be more suited to particular clinical areas than others. The Pain Ruler is a well established pain assessment tool which would seem to lend itself for use in the Emergency department setting more than some others. In particular the advantages are:

- It measures the intensity of the pain and the effects on normal function.
- It combines the use of verbal descriptors and a visual analogue scale
- It is quick and easy to use
- It is easily weighted to allow pain assessment to be part of the triage process
- By helping in the normal function assessment the nurse can become involved in the pain assessment process.
- It promotes dialogue which in turn encourages the patient that his pain is being taken seriously
- It produces a score facilitating on-going assessment.
- The outcome of the assessment is quick and easy to document.
- It can easily be adapted for use with children.

A pain ruler is shown in the figure

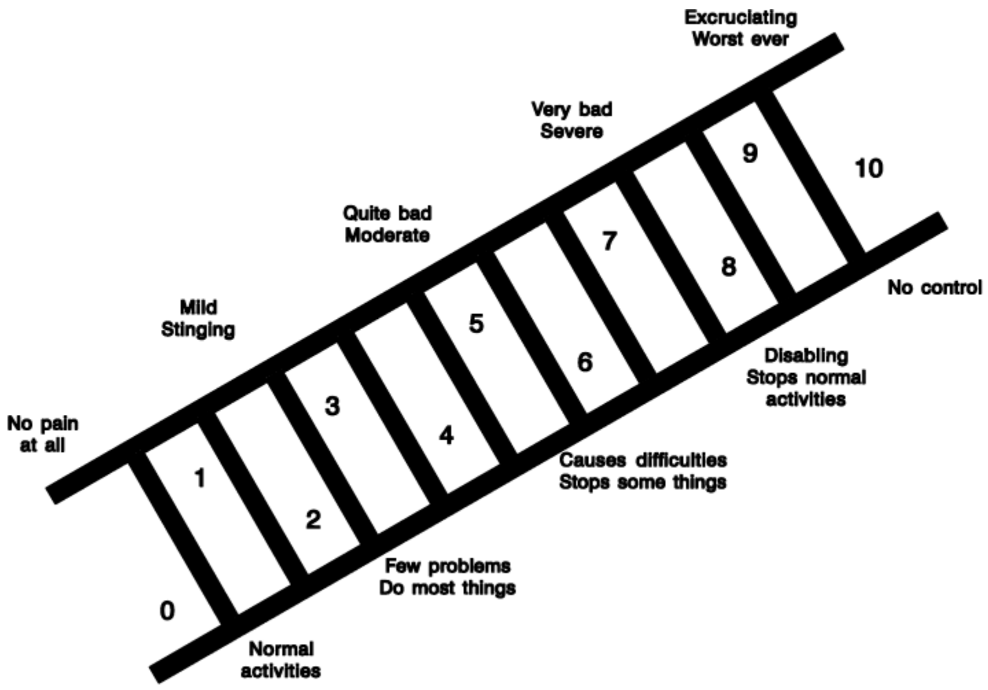

This can be supplemented by a pain scale for use with small children as shown in figure

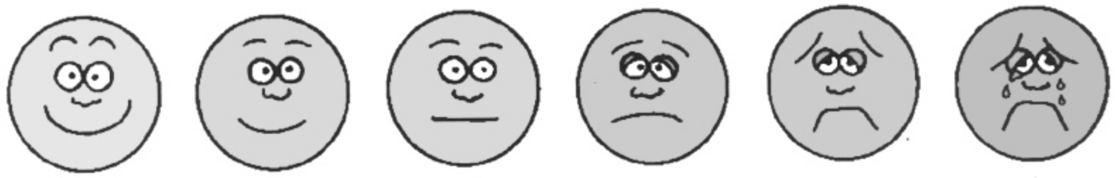

PAIN ASSESSMENT AT TRIAGE

Pain assessment is a skilled process in any environment and the assessment carried out during triage is no exception. There are particular constraints in this setting reflecting the emergency nature of the patients and the lack of assessment time. Nevertheless an accurate assessment of the patients pain into one of the categories shown in the figure is essential if proper and timely care is to be given. The triage nurse must take into account a number of factors that influence the patients perception of their pain.

Age

Children may imagine the worst possible outcome of their pain. They use catastrophic thinking which increases their anxiety and fear and may therefore enhance their perception of pain. Many elderly people suffer from multiple pain problems and may consider a significant pain level to be normal. Many accept pain and cope well with it.

Previous experience of pain

Patients are influenced by their previous experiences of pain. They may compare this pain to previous episodes as to whether this is more or less severe. They will also be influenced by how the pain was managed previously.

Culture

Illness behaviour, and therefore pain behaviour, has a strong cultural component, and because of different cultural and social influences, not all individuals express pain in the same way. Pain behaviours continue to be reinforced throughout life by the social group to which the individual belongs. Particular cultural groups do not feel pain less than others, they only differ in how they respond to, or express, their pain. It is essential that the triage nurse recognises that their own cultural and social background will inevitably influence how they interpret a patients pain behaviour. This identifies one particular difficulty with relying on assessment tools that consider only the patients (or the nurses) subjective assessment.

Anxiety

There is a link between high anxiety levels and high pain scoring. Patients can be anxious for a number of reasons, they may be concerned about the effect of this illness / accident on their ability to carry out their everyday activities, they may be anxious about attending hospital or worried about what is actually wrong with them. There are considerable benefits in addressing a patients pain at triage, in that the patient is shown at the earliest opportunity that his pain is being taken seriously. Reassurance and explanation from the triage nurse at this time may play a part in effectively reducing the level of pain.

Disruption to patients usual activities

Any individual functions at a level which is what he, or she, consider to be normal. Pain can destroy the patients ability to perform at that level, affecting their physical and emotional wellbeing, their financial situation and their position within society. Each patients perception of their pain will be influenced to some extent by how the pain will stop them functioning normally. It may not be possible to fully assess the level of disruption to the patients usual activities, but the nurse may be able to help the patient to focus on the effect of the pain. By asking pertinent questions such as - does the pain stop you eating / drinking / sleeping / breathing properly?, does the pain stop you walking / sitting etc ? does the pain stop you working / going to school?

If a patient scores his pain as 10 but then is able to perform all his usual activities, the nurse should consider other factors that may be influencing the patients assessment of his pain.

PATIENT MANAGEMENT, TRIAGE AND THE TRIAGE NURSE

There is a difference between absolute clinical priority and relative priority within and between triage categories. In overview the process of triage as outlined here is quite simple - patients are assigned to a triage category and then managed in order of priority and time of attendance. However there are many other factors apart from clinical priority which may from time to time influence how the patient is handled within the Emergency Department. This section outlines these other factors and discusses their importance. Clinical priority, and the factors that determine it are clearly very important, but failure to recognise other factors can be detrimental to both departmental function and quality of care for individual patients.

TYPE OF PATIENT

There are a number of issues about the nature of individual patients that affect their management in addition to their clinical priority. These are summarised below:

Children

Children may need special management, especially in Emergency Departments without special paediatric facilities. They are always accompanied by some one else (usually a parent but teachers, relatives, social workers may also be present), as are siblings and friends who, although well, need entertaining. Children have very short attention spans and get bored, frightened, and tired very easily. They may get very distressed and agitated because of communication and understanding difficulties, and this makes later handling more difficult. Children who can be happily entertained by a play leader or in a separate waiting room with play facilities, probably do not need any special attention other than frequent reassessment. It is helpful if child friendly food and drink eg snacks and drinks in cartons, bottles etc are available (provided the carer of any child who may need a general anaesthetic or sedation is aware of the need to keep the child nil by mouth). It may be worth while having a special policy for children who present late in the evening or at night. The child who is very tired may prove impossible to examine and treat, so a relatively early examination may be considered.

Elders

Relative immobility can cause increased discomfort in the waiting room and may cause difficulty reaching the toilet or going for refreshments. A person who is normally able to cope well in familiar surroundings may become quite confused and disorientated in the Emergency Department even if only slightly injured. The elderly are often set in a routine and become anxious if unable to meet their normal time table. There may be carers at home who have responsibilities who would need to be informed about the elderly patients attendance. The elderly are very prone to pressure sores which can develop after only half an hour on a hospital trolley. If they cannot be seen quickly for treatment they need frequent nursing attention for turning. They may have problems with continence which if not anticipated may lead to embarrassment. Memory problems may lead to poor information giving. Practitioners should be aware of these issues and consider the relative needs of this group of patients.

Patients with physical disability or learning difficulties

Apart from the extremes of age, there will be the patients who have particular difficulties. These include those with special needs, poor sight, poor hearing etc. Persons who can cope quite well in the community under controlled circumstances may have great difficulties in the strange environment of the Emergency Department. Communications again become particularly important, and it may be appropriate for such patients to be seen relatively quickly.

Abusive / aggressive patients

There are few things worse than having a full waiting room, with one or more patients (or more often relatives or friends of patients) constantly demanding attention. Although the guiding principle must be that these patients are not given priority just because they shout louder the distress they cause to others must be taken into consideration. An initial attempt to communicate departmental policy may be followed by a number of actions. The patient may be placed in an individual cubicle to wait in order to minimise the disruption to the waiting room. Alternatively such patients can be seen, treated and discharged rapidly for the benefit of others. If all else fails the patient (or the patients relatives) may be asked to leave, assisted as necessary by security or police.

Patients under the influence of alcohol

These patients are difficult to assess because of the effect of alcohol on conscious level, and on pain perception. They need frequent assessment to check that they are not deteriorating or developing a problem not immediately apparent at triage. Disruptive drunk patients should be treated as outlined above.

The Regular

Most departments have a number of patients who are frequent attenders. It is undoubtably tempting to place these patients into the non-urgent category without proper assessment. Beware, even the regulars develop organic pathology, injure themselves or have a serious complication of their disease. These patients (even those with predominantly social problems) are in fact more likely to develop illnesses or sustain injuries than the general population. Each attendance should be treated as a new visit and proper assessment should be undertaken; this avoids underestimation of possible serious causes for attending.

Patients who re-attend

There are occasions when a patient returns to the department, usually because their original presenting complaint has not resolved or has developed a complication. Sometimes the patients expectations of the natural progress if an injury or illness is unrealistic. The patient may also return having failed to wait for definitive treatment on a prior occasion. The patient should be allocated a triage category according to the symptoms presenting at the time of triage, and not according to the original triage category. Some departments may have policies recommending such patients are reviewed by a senior doctor if available. It may also be appropriate to offer some of these patients a review clinic appointment for assessment by a senior doctor if the problem does not seem to need immediate treatment.

Patients referred by other hospitals

Many departments allow their facilities to be used by other teams for assessment and referral of severely ill patients. These patients are usually pre-arranged or accepted patients from primary care Physicians or hospitals. They are often patients who are accepted for possible admission and many have a relatively high clinical priority. *These patients must be triaged in the same way as Emergency Department patients*. If the patient is triaged as first priority it would be usual for the Emergency department team to initiate resuscitation, unless the referral team is in the department. The triage nurse should inform the referral team of the triage category of the patients in order to try and ensure that these patients are treated with a similar degree of urgency as Emergency Department patients. It may also be appropriate for the triage nurse to ask departmental clinical staff to provide analgesia or initiate immediate investigations, in order to smooth the patients stay in the department. Some patients may have been brought in by the police (for instance under mental health legislation), by social services or by other professional services. Triage practitioners should be aware of the pressures on staff from other agencies and consider this when deciding the relative priority of such patients.

ROLE OF THE TRIAGE NURSE

The triage nurse's main role is the accurate priorisation of patients, and this must be the prime objective. The triage nurse needs to become accomplished at rapid assessment – this involves quick decision making and suitable delegation of tasks. Long conversations with patients should be avoided as should exhaustive history taking. Clinical observations such as temperature/pulse etc need to be delegated if they are not required to establish priority as they are too time consuming. In small departments the triage nurse will see all patients coming in the department. In others there may be separate nurses dealing with walking and stretcher patients. The method of arrival of the patient does not always accord with the seriousness of the illness. Therefore there must be close liaison between triage staff in order to place the patients correctly. The triage method here proposed should assist this process by standardising triage practice. Rapid influxes of patients may require the triage nurse to seek assistance from another member of staff. The triage process is integral to the clinical management of most departments, and a variety of additional asks may be undertaken.

First Aid/Analgesia

The triage nurse may need to provide or facilitate some first aid treatment, and recognise the need to provide analgesics if required (see pain). Application of a sling or dressing will immediately improve patient's comfort and help minimise further trauma and bleeding.

Patients information

The triage nurse is the first clinical contact for the patient, and talking the patient through the illness and probable course in the department alleviates much distress and anxiety. Patients appreciate knowing the waiting time, the probable time spent in the department, whether any investigations may be ordered and possible treatment. This information can be provided quite quickly for most common conditions.

Health promotion

The triage nurse (if times allows) can usefully act as a health promoter. The patient is quite receptive to health care advice when an adverse event has occurred. If possible brief advice about relevant topics such as locked cabinets, cycle helmets and stopping smoking may be appropriate. It is helpful if patients information leaflets are available.

Disposition of patients around the department

The triage nurse will often have to decide where to place the patients in the department. This will depend on departmental facilities and policies. Patients who are distressed, in pain, bleeding or at extremes of age may be best placed in cubicles away from the general waiting room. Patients who need to be lying down for examination (for example those suffering from knee injuries, back complaints and abdominal pain) should be placed in an area where they can lie down. Ill patients may well walk into the department and may need to be placed in the appropriate area of the department. To achieve this the triage nurse needs to be continuously aware of the occupancy of the department and the current disposition of patients.

Managing the waiting room

Until they have been seen by the clinician the patients' main contact is with the triage nurse. Further advice may be sought by these patients, and criticisms delivered. The triage nurse needs to keep the waiting room informed of the current approximate waiting time. Constant observation and reassessment is necessary in order to spot those patients whose condition is changing. Triage is a dynamic process and the patients often need regular reassessment. This might occur after an intervention eg the administration of analgesic, or after an appropriate length of time. Patients may be dropped into a lower category after pain relief or brought forward if they deteriorate. No one can anticipate all problems and it is not a 'failure' of accurate assessment to change the triage category according to further developments in the patients condition, or indeed with further information that may be acquired. The waiting room should be considered to be a clinical area.

Discriminators dictionary

Abdominal pain	Any pain felt in the abdomen. Abdominal pain associated with back pain may indicate abdominal aortic aneurysm, whilst association with PV bleeding may indicate ectopic pregnancy or miscarriage.
Abnormal pulse	A bradycardia (<60 min in adults), a tachycardia (> 100 min in adults) or an irregular rhythm. Age appropriate definitions of bradycardia and tachycardia should be used in children.
Abrupt onset	Onset within seconds or minutes. May cause waking from sleep.
Acute chemical eye injury	Any substance splashed into or placed into the eye within the past 24 hours that caused stinging, burning, or reduced vision should be assumed to be have caused chemical injury.
Acute complete loss of vision	Loss of vision in one or both eyes within the preceding 24 hours

Acute neurological deficit	Any loss of neurological function that has come on within the previous 24 hours. This might include altered or lost sensation, weakness of the limbs (either transiently or permanently) and alterations in bladder or bowel function.
Acute onset after injury	Onset of symptoms immediately within 24 hours of a physically traumatic event.
Acutely avulsed tooth	A tooth that has been avulsed intact within the previous 24 hours.
Acutely short of breath	Shortness of breath that comes on suddenly, or a sudden exacerbation of chronic shortness of breath.
Age less than 25 years	25 years old or younger.
Airway compromise	An airway may be compromised either because it cannot be kept open or because the airway protective reflexes (that stop inhalation) have been lost. Failure to keep the airway open will result either in intermittent total obstruction or in partial obstruction. This will manifest itself as snoring or bubbling sounds during breathing.
Altered blood	Darker than fresh blood and often smelling more like melaena.
Altered conscious level	Not fully alert. Either responding to voice or pain only or unresponsive.
Altered conscious level not wholly attributable to alcohol	A patient who is not fully alert, with a history of alcohol ingestion, in whom there is any doubt at all that other causes of reduced conscious level may be present fulfils this discriminator definition.
Altered conscious level wholly attributable to alcohol	A patient who is not fully alert, with a clear history of alcohol ingestion, and in whom there is no doubt that all other causes of reduced conscious level have been excluded fulfils this discriminator definition.
Altered facial sensation	Any alteration of sensation on the face
Atypical behaviour	A child that is behaving in a way that is not usual in the given situation. The carers will often volunteer this information. Such children are often referred to as fractious or "out of sorts".
Auricular haematoma	A tense haematoma (usually post traumatic) in the outer ear.
Black stool	Any blackness fulfils this criterion.
Bleeding disorder	Congenital or acquired bleeding disorder.
Breathing after airway opened	In major incidents the presence of breathing after simple airway opening manoeuvres allows respiratory rate to be counted. Absence of breathing when the airway is open indicates death.
Capillary refill time	The capillary refill time is the time taken for the nail bed capillaries to refill after pressure has been applied for 5 seconds. The normal time is less than 2 seconds. This sign is less useful if the patient is cold.
Capillary refill time abnormal	Major incident casualties whose capillary refill is prolonged (more than 2 seconds) are categorised as RED
Cardiac pain	Classically a severe dull "gripping" or "heavy" pain in the centre of the chest, radiating to the left arm or to the neck. May be associated with sweating and nausea.
Chemical injury	Any substance splashed onto or placed onto the body that causes stinging, burning, reduced vision or any other symptoms should be assumed to be capable of causing a chemical injury.
Chest infection	A chest infection usually causes a cough and production of sputum. This is usually purulent (green or yellow).
Chest injury	Any injury to the area below the clavicles and above the level of the lowest rib.

	Injury to the lower part of the chest can cause underlying damage to abdominal organs.
Cold	If the skin feels cold the patient is clinically said to be cold. The temperature should be taken as soon as possible - a core temperature < 35 C is cold.
Colicky pain	Pain that comes and goes in waves. Renal colic tends to come and go over 20 minutes or so.
Critical skin	A fracture or dislocation may leave fragments or ends of bone pressing so hard against the skin that the viability of the skin is threatened. The skin will be white and under tension.
Currently fitting	Patients who are in the tonic or clonic stages of a grand mal convulsion, and patients currently experiencing partial fits fulfil this criteria.
Current palpitation	A feeling of the heart racing (often described as a fluttering) that is still present.
Deformity	This will always be subjective. Abnormal angulation or rotation is implied.
Diplopia	Seeing double.
Direct trauma to the back	This may be top to bottom (loading) for instance when someone falls and lands on their feet, bending (forwards, backwards or to the side) or twisting.
Direct trauma to the neck	This may be top to bottom (loading) for instance when something falls on the head, bending (forwards, backwards or to the side), twisting, or distracting such as in hanging.
Discharge	In the context of sexually acquired infection this is any discharge from the penis or abnormal discharge from the vagina.
Disruptive	Disruptive behaviour is behaviour that affects the smooth running of the department. It may or may not be threatening.
Distal vascular compromise	There will be a combination of pallor, coldness, altered sensation, and pain with or without absent pulses distal to the injury.
Distressed by pain	A child that is distressed by pain and inconsolable.
Drooling	Saliva running from the mouth as a result of being unable to swallow.
Dysuria	Pain or difficulty in passing urine. Pain is typically described as stinging or hot.
Electrical injury	Any injury caused or possibly caused by electric current. This includes AC and DC and both artificial and natural sources.
Exhaustion	An exhausted patient appears to reduce the effort they make to breathe despite continuing respiratory insufficiency. This is preterminal.
Exsanguinating haemorrhage	Haemorrhage which is occurring at such a rate that death will ensue unless bleeding is stopped.
Externalisation of organs	Herniation or frank extrusion of internal organs.
Eye injury	A recent physically traumatic event to the eye.
Facial oedema	Diffuse swelling around the face usually involving the lips.
Facial swelling	Swelling around the face which may be localised or diffuse.
Fails to react to parents	Failure to react in any way to a parents face or voice. Abnormal reactions and apparent lack of recognition of a parent are also worrying signs.
Floppy	Parents may describe their children as floppy. Tone is generally reduced - the most noticeable sign is often lolling of the head.
Focal or progressive loss of function	Loss of function that is limited to a particular part of the body (limb, side, eye etc) or a loss of function that is getting worse over hours.
Foreign body sensation	A sensation of something in the eye, often expressed as scraping or grittiness.

Frank haematuria	Red discolouration of the urine caused by blood.
Fresh blood	Unaltered blood - readily identified by both the patient and their carers.
Generalised rash	The rash may be of any form - but will usually be erythematous or urticarial.
Gross deformity	This will always be subjective. Gross and abnormal angulation or rotation is implied.
Headache	Any pain around the head that is not related to a particular anatomical structure. Facial pain is not included.
Head injury	Any traumatic event involving the head fulfils this criteria.
Heavy PV blood loss	PV loss is extremely difficult to assess. The presence of large clots or constant flow fulfil this criteria. The use of large numbers of sanitary towels is suggestive of heavy loss.
High blood pressure	A history of raised blood pressure or a raised blood pressure on examination.
High lethality	Lethality is the potential of the substance taken to cause harm. Advice from a poisons centre may be required to establish the level of risk of serious illness or death. If in doubt assume a high risk.
High lethality envenomation	Lethality is the potential of the envenomation to cause harm. Local knowledge may allow identification of the venomous creature, but advice may be required. If in doubt assume a high risk.
High risk of (further) harm to others	The presence of a potential risk of harm to others can be judged by looking at posture (tense and clenched), speech patterns (loud and using threatening words), and motor behaviour (restless, pacing). High risk should be assumed if weapons and potential victims are available, or if self control is lost.
High risk of (further) self harm	An initial view of the risk of self harm can be formed by considering the patients behaviour. Patients who have a significant history of self harm, are actively trying to harm themselves or who are actively trying to leave with the intent of harming themselves are at high risk.
History of acutely vomiting blood	Frank haematemesis, vomiting of altered blood (coffee ground) or of blood mixed in the vomit within the past 24 hours.
History of fitting	Any observed or reported fits that have occurred during the period of illness or following and episode of trauma.
History of recent foreign travel	Recent foreign travel (within 2weeks).
History of head injury	A history of a recent physically traumatic event involving the head. Usually this will be reported by the patient but, if the patient has been unconscious this history should be sought from a reliable witness.
History of overdose or poisoning	This information may come from others or may be deduced if medication is missing.
History of trauma	A history of a recent physically traumatic event.
History of unconsciousness	There may be a reliable witness who can state whether the patient was unconscious (and for how long). If not a patient who is unable to remember the incident should be assumed to have been unconscious.
Hot joint	Any warmth around a joint fulfils this criterion. Often accompanied by redness.
Hot child	If the skin feels hot the child is clinically said to be hot. The temperature should be taken as soon as possible - a temperature > 38.5 C is hot.

Hot adult	A temperature > 38.5 C is hot.
Hyperglycaemic with ketosis	Glucose greater than 400 mg/dL with urinary ketones or signs of acidosis (deep sighing respiration etc).
Hyperglycaemic	Glucose greater than 800 mg/dL.
Hypoglycaemic	Glucose less than 50 mg/dL.
In active labour	A woman who is having regular frequent painful contractions fulfils this criterion.
Inability to weight bear	Inability to carry the full weight of the body through one or both lower limbs. This may be because of pain or loss of function.
Inadequate breathing	Patients who are failing to breathe well enough to maintain adequate oxygenation have inadequate breathing. There may be a increased work of breathing, signs of inadequate breathing, or exhaustion.
Inadequate history	If there is not a clear and unequivocal history of acute alcohol ingestion, and if head injury, drug ingestion, underlying medical condition etc. cannot be definitely excluded then the history is inadequate.
Inappropriate history	When the history (story) given does not explain the physical findings it is termed inappropriate. This is important as it is a marker of non-accidental injury in vulnerable children and adults and may be the sentinel for abuse.
Increased work of breathing	Increased work of breathing is shown as increased respiratory rate, use of accessory muscles and grunting.
Inconsolable by parents	Children whose crying or distress does not respond to attempts by their parents to comfort them, fulfil this criteria.
Inhalational injury	A history of being confined in a smoke filled space is the most reliable indicator of smoke inhalation. Carbon deposits around the mouth and nose and hoarse voice may be present. History is also the most reliable way of diagnosing inhalation of chemicals - there will not necessarily be any signs.
Inhalational chemical injury	A history of having inhaled a potentially hazardous chemical. Some chemicals may leave specific signs while others may not. The nature of the hazard may not be immediately apparent.
Insecure airway	Patients who cannot continually maintain their own airway have an insecure airway.
Known immunosuppression	Any patient on immunosuppressive drugs (including long term steroids) or who is HIV positive.
Lethality	The potential of the substance taken to cause illness or death. Advice from a poisons centre may be required to establish this. If in doubt assume a high risk.
Local infection	Local infection usually manifests as inflammation (pain, swelling and redness) confined to a particular site or area, with or without a collection of pus.
Local inflammation	Local inflammation will involve pain, swelling and redness confined to a particular site or area.
Low SaO2	This is a saturation of < 95% on air.
Marked distress	Patients who are markedly physically or emotionally upset fulfil this criterion.
Moderate itch	An itch that is bearable but intense.
Moderate lethality	Lethality is the potential of the substance taken to cause serious illness or death. Advice from a poisons centre may be required to establish the level of risk to the patient.

Moderate lethality envenomation	Lethality is the potential of the envenomation to cause harm. Local knowledge may allow identification of the venomous creature, but advice may be required.
Moderate pain	Pain that is bearable but intense. See the chapter on pain assessment.
Moderate risk of (further) harm to others	The presence of a potential risk of harm to others can be judged by looking at posture (tense and clenched), speech patterns (loud and using threatening words), and motor behaviour (restless, pacing). Moderate risk should be assumed if there is any indication of potential harm to others.
Moderate risk of (further) self harm	An initial view of the risk of self harm can be formed by considering the patients behaviour. Patients without a significant history of self harm, who are not actively trying to harm themselves, who are not actively trying to leave with the intent of harming themselves, but who profess the desire to harm themselves are at moderate risk.
New neurological deficit	Any loss of neurological function including altered or lost sensation, weakness of the limbs (either transiently or permanently) and alterations in bladder or bowel function.
Non-blanching rash	A rash that does not blanche (go white) when pressure is applied to it. Often tested using a glass tumbler to apply pressure as any colour change can be observed through the bottom of the tumbler.
No improvement with own asthma medications	This history should be available from the patient. A failure to improve with bronchodilator therapy given by the GP or paramedic is equally significant.
Normal menstruation	Menstrual blood loss and pain occurring on the expected date for the expected length of time.
Not distractible	Children who are distressed by pain or other things who cannot be distracted by conversation or play fulfil this criteria.
Not feeding	Children who will not take any solid or liquid (as appropriate) by mouth. Children who will take the food but always vomit afterwards may also fulfil this criterion.
Not passing urine	Failure to produce and pass urine. This may be difficult to judge in children (and the elderly) and reference to the number of nappies or pads used may be useful.
Oedema of the tongue	Swelling of the tongue of any degree.
Open fracture	All wounds in the vicinity of a fracture should be regarded with suspicion. if there is any possibility of communication between the wound and the fracture then the fracture should be assumed to be open.
Pain on joint movement	This can be pain on either active (patient) movement or passive (examiner) movement.
Pain radiating to the back	Pain that is also felt in the back either intermittently of constantly.
Passing fresh or altered blood PR	In active massive GI bleeding dark red blood will be passed PR. As GI transit time increases this becomes darker - eventually becoming melaena.
Penetrating eye injury	A recent physically traumatic event involving penetration of the globe.
Penetrating trauma	A recent physically traumatic event which involves discrete penetration of any body area by a knife, bullet or other object.
Persistent vomiting	Vomiting that is continuous or that occurs without any respite between episodes.
Pleuritic pain	A sharp, localised pain in the chest worse on breathing, coughing or sneezing.
Possibly pregnant	Any woman whose normal menstruation has failed to occur is possibly pregnant. Furthermore any woman of childbearing age that is having unprotected sex should be considered to be potentially pregnant.

Presenting foetal parts	Crowning or presentation of any other foetal part in the vagina.
Priapism	Sustained penile erection
Prolapsed umbilical cord	Prolapse of any part of the umbilical cord through the cervix.
Prolonged or uninterrupted crying	A child who has cried continuously for 2 hours or more fulfils this criteria.
Pulse rate abnormal	If the capillary refill time cannot be measured then major incident casualties whose pulse is raised over 120 beats per minute are categorised RED.
Purpura	A rash on any part of the body that is cause by small haemorrhages under the skin. A purpuric rash does not blanche (go white) when pressure is applied to it.
PV Blood loss and more tan 20 weeks pregnant	Any loss of blood per vaginum in a woman known to be beyond the 20th week of pregnancy
PV blood loss	Any loss of blood per vaginum
Rapid onset	Onset within the preceding 12 hours.
Recent hearing loss	Loss of hearing in one or both ears within the previous week
Recent injury	An injury occurring within the last week is said to be recent.
Recent mild itch	Any itch that has occurred in the past 7 days.
Recent mild pain	Any pain that has occurred within the past 7 days.
Recent problem	A problem arising in the last week is said to be recent.
Recent reduced visual acuity	Any reduction in corrected visual acuity within the past 7 days.
Recent signs of mild pain	Young children and babies in pain cannot complain. They will usually cry occasionally and may act atypically.
Red eye	Any redness to the eye. A red eye may be painful or painless and may be complete or partial.
Redcurrant stool	A dark red stool classically seen in intersussception. Absence of this type of stool does not rule out the diagnosis.
Respiratory rate	Major incident casualties whose respiratory rate is abnormal either by being too high (over 29) or too low (less than 10) are categorised as RED
Responds to pain	Response to a painful stimulus. Standard peripheral stimuli should be used - a pencil or pen is used to apply pressure to the finger nail bed. This stimulus should not be applied to the toes since a spinal reflex may cause flexion even in brain death. Supraorbital ridge pressure should not be used sine reflex grimacing may occur.
Responds to voice	Response to a vocal stimulus. It is not necessary to shout the patients name. Children may fail to respond because they are afraid.
Retention of urine	Inability to pass urine per urethra associated with an enlarged bladder. This condition is usually very painful unless there is altered sensation.
Risk of continued contamination	If chemical exposure is likely to continue (usually due to lack of adequate decontamination) then this discriminator applies. Risks to health care workers must not be forgotten if this situation occurs.
Risk of harm to others	The potential of the patient to actively attempt to harm others. This may be assessed by considering the state of mind, body posture and behaviour. If in doubt assume a high risk
Risk of self harm	The potential of the patient to actively attempt further self harm. If in doubt assume a high risk.

Scalp haematoma	A raised bruised area to the scalp (bruises below the hair line at the front are to the forehead.
Scrotal cellulitis	Redness and swelling around the scrotum.
Scrotal gangrene	Dead blackened skin around the scrotum and groin. Early gangrene may not be black but may appear like a full thickness burn with or without flaking.
Scrotal trauma	Any recent physically traumatic event involving the scrotum.
Severe pain	Pain that is unbearable - often described as the worst ever. See the chapter on pain assessment.
Severe itch	An itch that is unbearable.
Shock	Shock is inadequate delivery of oxygen to the tissues. The classical signs include sweating, pallor, tachycardia, hypotension and reduced conscious level.
Shoulder tip pain	Pain felt in the tip of the shoulder. This often indicates diaphragmatic irritation.
Significant cardiac history	A known recurrent dysrhythmia which has life-threatening effects is significant as is a known cardiac condition that may deteriorate rapidly.
Significant mechanism of injury	Penetrating injuries (stab or gunshot) and injuries with high energy transfer such as falls from heights and high speed road traffic accidents (speed > 40 mph) are significant especially if there has been ejection from the vehicle, the death(s) of other victim(s) of the accident or marked deformation of the vehicle.
Significant haematological history	A patient with a haematological disorder that is known to deteriorate rapidly
Significant history of allergy	A known sensitivity with severe reaction (eg. to nuts or bee sting) is significant.
Significant history of GI bleed	Any history of massive GI bleeding or of any GI bleed associated with oesophageal varices
Significant respiratory history	A history of previous life threatening episodes of a respiratory condition (eg COPD) is significant as is brittle asthma.
Significant medical history	Any pre-existing medical condition requiring continual medication or other care.
Significant psychiatric history	A history of a major psychiatric illness or event.
Signs of dehydration	These include dry tongue, sunken eyes, increased skin turgor and, in small babies, a sunken anterior fontanelle. Usually associated with a low urine output.
Signs of meningism	Classically a stiff neck together with headache and photophobia.
Signs of moderate pain	Young children and babies in moderate pain cannot complain. They will usually cry intermittently and are often intermittently consolable.
Signs of severe pain	Young children and babies in severe pain cannot complain. They will usually cry out continuously and inconsolably and be tachycardic. They may well exhibit signs such as pallor and sweating.
Smoke inhalation	Smoke inhalation should be assumed if the patient has been confined in a smoke filled space. Physical signs such as oral or nasal soot are less reliable but significant if present.
Special risk of infection	Known exposure to a dangerous pathogen, or travel to an area with an identified, current serious infectious risk.
Stridor	This may be an inspiratory or expiratory noise, or both. Stridor is heard best on breathing with the mouth open.
Subcutaneous gas	Gas under the skin can be detected by feeling for a "crackling" on touch. There may be gas bubbles and a line of demarcation.
Swelling	An abnormal increase in size

Temporal scalp tenderness	Tenderness on palpation over the temporal area (especially over the artery).
Testicular pain	Pain in the testicles
Threatening to others	Patients with threatening posture (tense and clenched), speech patterns (loud and using threatening words), and motor behaviour (restless, pacing).
TRTS	Triage revised trauma score: this is calculated using the coded respiratory rate (0 - 4), the systolic blood pressure (0 - 4) and the Glasgow Coma Scale score (0 - 4) to give a score from 0 - 12.. This scoring system is shown on most triage labels.
Unable to feed	This is usually reported by the parents. Children who will not take any solid or liquid (as appropriate) by mouth.
Unable to talk in sentences	Patients who are so breathless that they cannot complete relatively short sentences in one breath.
Unable to walk	It is important to try and distinguish between patients who have pain and difficulty walking, and those who cannot walk. Only the latter can be said to be unable to walk.
Uncontrollable major haemorrhage	A haemorrhage that is not rapidly controlled by the application of sustained direct pressure, and which continues to bleed heavily or soak through large dressings quickly.
Uncontrollable minor haemorrhage	A haemorrhage that is not rapidly controlled by the application of sustained direct pressure, and which continues to bleed slightly or ooze.
Unresponsive	Patients who fail to respond to either verbal or painful stimuli are unresponsive.
Unresponsive child	A child who fails to respond to either verbal or painful stimuli is unresponsive.
Vaginal trauma	Any history or other evidence of direct trauma to the vagina fulfils this criteria.
Vascular compromise	There will be a combination of pallor, coldness, altered sensation, and pain with or without absent pulses distal to the injury.
Vertigo	An acute feeling of spinning or dizziness, possibly accompanied by nausea and vomiting.
Very low SaO2	This is a saturation < 95% on O2 therapy or < 90% on air.
Very hot adult	If the skin feels very hot the patient is clinically said to be very hot. The temperature should be taken as soon as possible - a temperature > 40 C is very hot.
Visible abdominal mass	A mass in the abdomen that is visible to the naked eye.
Vomiting	Any emesis fulfils this criteriion.
Vomiting blood	Vomited blood may be fresh (bright or dark red) or coffee ground in appearance.
Walking	In a major incident any patient who can walk fulfils this criteria.
Warmth	If the skin feels warm the patient is clinically said to be warm. The temperature should be taken as soon as possible - a temperature > 37.5 C is warm.
Wheeze	This can be audible wheeze or a feeling of wheeze. Very severe airway obstruction is silent (no air can move).
Wound contamination	A wound that contains extrinsic material of any description is said to be contaminated.
Widespread rash or blistering	Any discharging or blistering eruption covering more than 10% body surface area.
