# Supplementary material for: Effect of triage training on the knowledge application and practice improvement among the practicing nurses of the emergency departments of the National Referral Hospitals, 2018; a pre-post study in Asmara, Eritrea
Source: BMC Emerg Med. 2022 Dec 2;22:190. doi: 10.1186/s12873-022-00755-w (PMC9719223; doi:10.1186/s12873-022-00755-w)
Supplement: Supplementary file 2 — Additional file 2. [file 12873_2022_755_MOESM2_ESM.pdf]

Abdominal pain in adults

Orotta TS — 01 June 2007 - V. 1.1

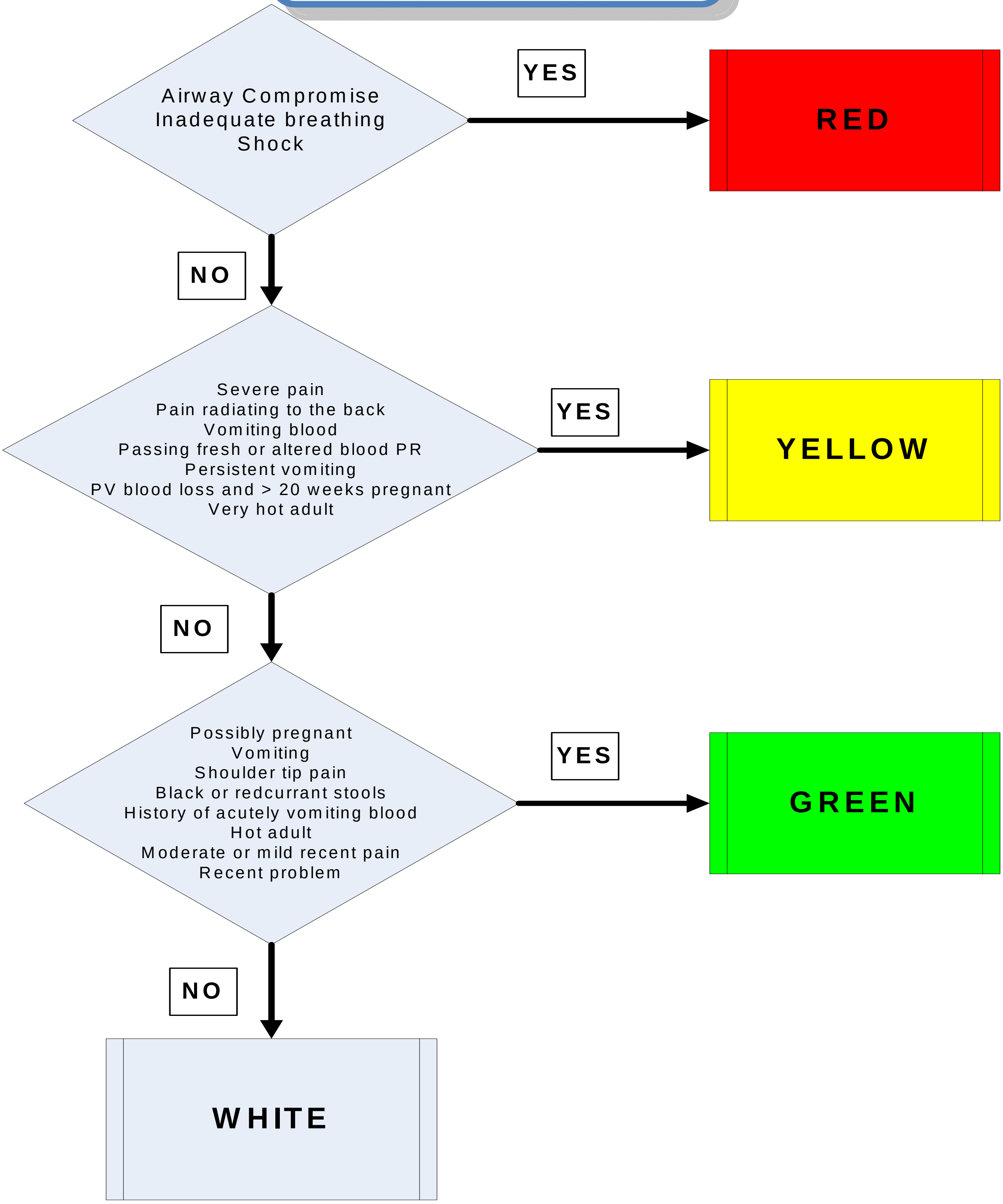

See also:	Chart notes:
GI Bleeding,<br>Diarrhoea and Vomiting,<br>Pregnancy	This is a presentation defined flow diagram. Abdominal pain is a common cause of presentation of surgical emergencies. A number of general discriminators are used including Life Threat and Pain. Specific discriminators are included in the GREEN and YELLOW categories to ensure that the more severe pathologies are appropriately triaged. In particular discriminators are included to ensure that patients with moderate and severe GI Bleeding and those with signs of retroperitoneal or diaphragmatic irritation are given sufficiently high categorisation.

Abdominal pain in children

Orotta TS - 01 June 2007 - V. 1.1

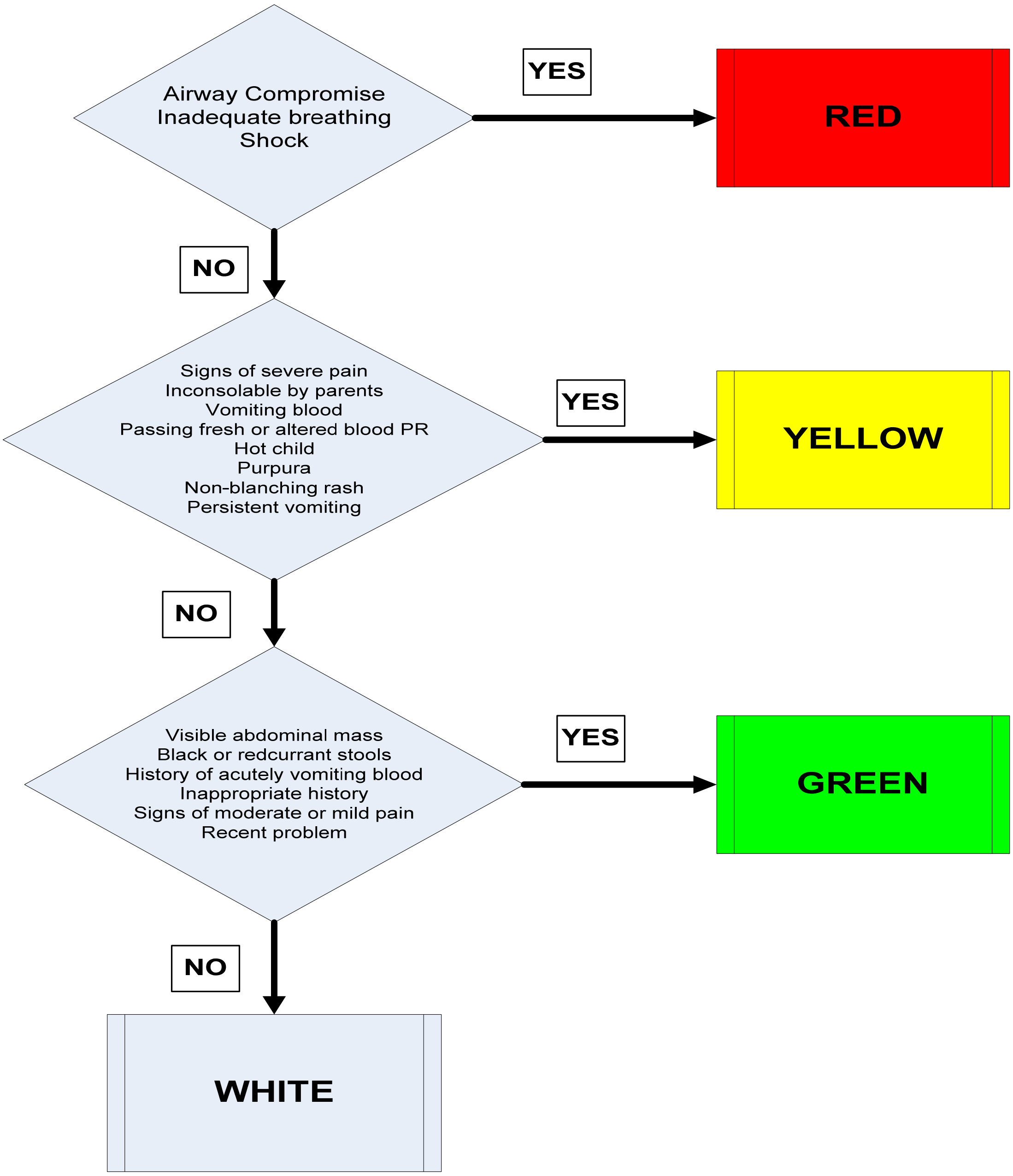

See also:	Chart notes:
Diarrhoea and Vomiting	This is a presentation defined flow diagram. Children who present with abdominal pain may have a range of pathologies and this chart has been designed to allow them to be accurately prioritised. A number of general discriminators are used including Life Threat and Pain. Specific discriminators are included to ensure the children who are actively bleeding, and those who have the signs or symptoms of more severe pathologies such as intersussception are seen urgently.

Abscesses and Local Infections

Orotta TS - 01 June 2007 - V. 1.1

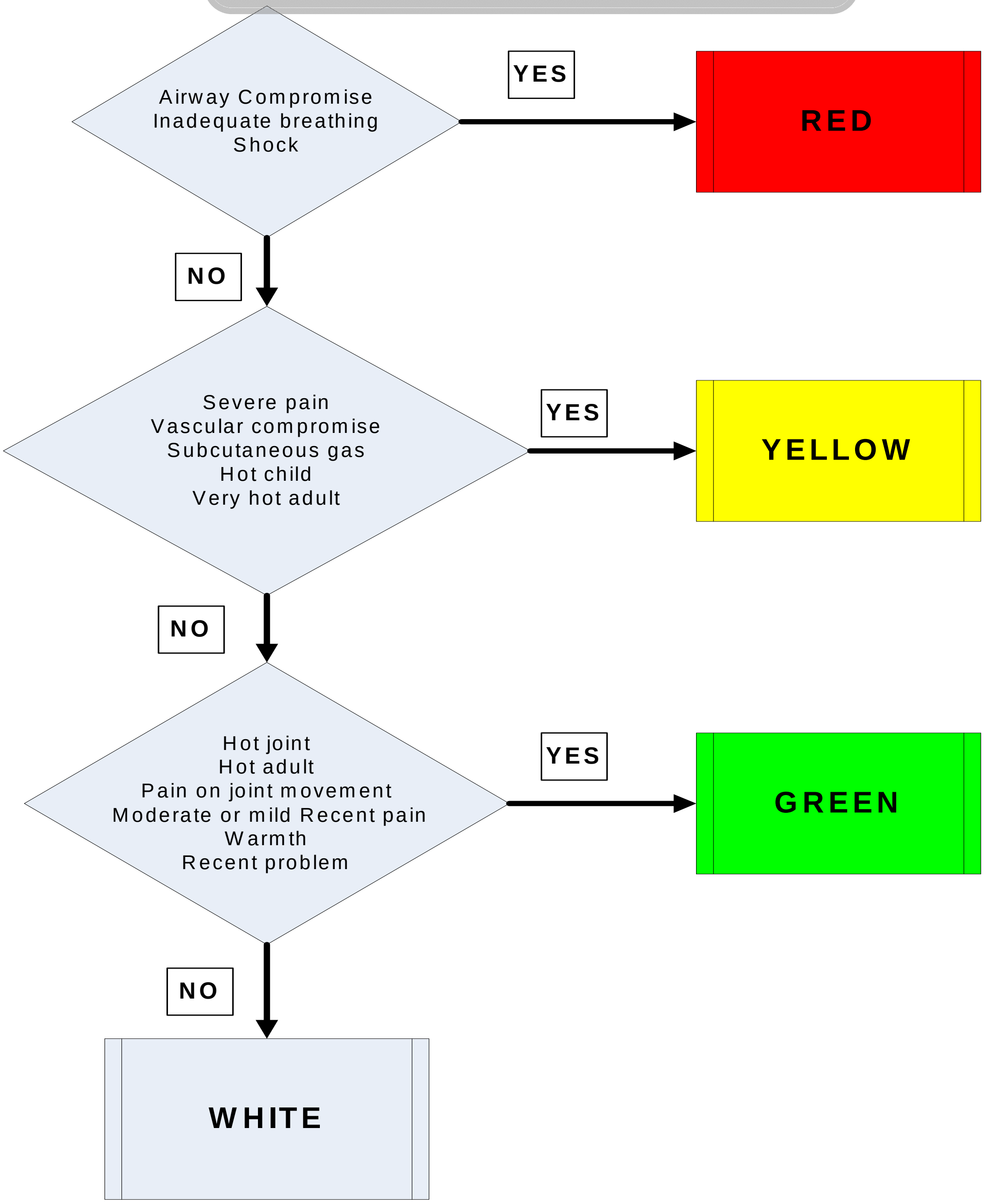

See also:	Chart notes:
Bites and stings	This is a presentation defined flow diagram designed to allow prioritisation of patients who present with a variety of obvious local infections and abscesses. Underlying conditions may vary from life threatening orbital cellulitis to acneiform spots. A number of general discriminators are used including Life Threat, Pain and Temperature. Specific discriminators have been included to allow identification of more urgent conditions such as gas gangrene and septic arthritis.

Allergy

Orotta TS - 01 June 2007 - V. 1.1

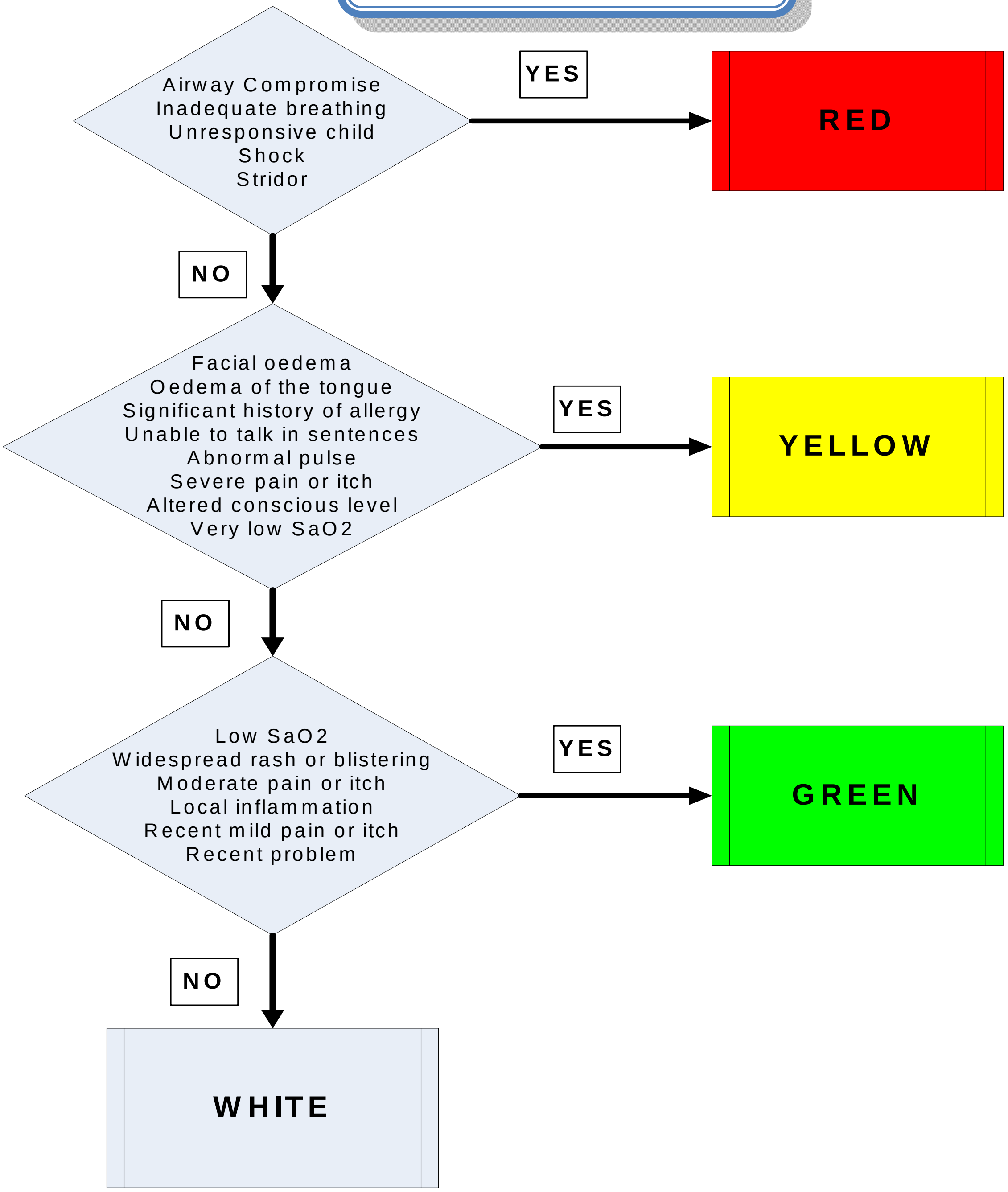

See also:	Chart notes:
Collapsed Adult<br>Unwell Adult<br>Asthma<br>Bites and Stings	This is a presentation defined flow diagram designed to allow prioritisation of patients who present with symptoms and signs that may indicate allergy. Patients with allergic reactions range from those with life-threatening anaphylaxis to those with an itchy insect bite. A number of general discriminators are used including Life Threat, Conscious Level and Pain. Specific discriminators have been included to allow prioritisation of the most urgent conditions.

Apparently Drunk

Orotta TS - 01 June 2007 - V. 1.1

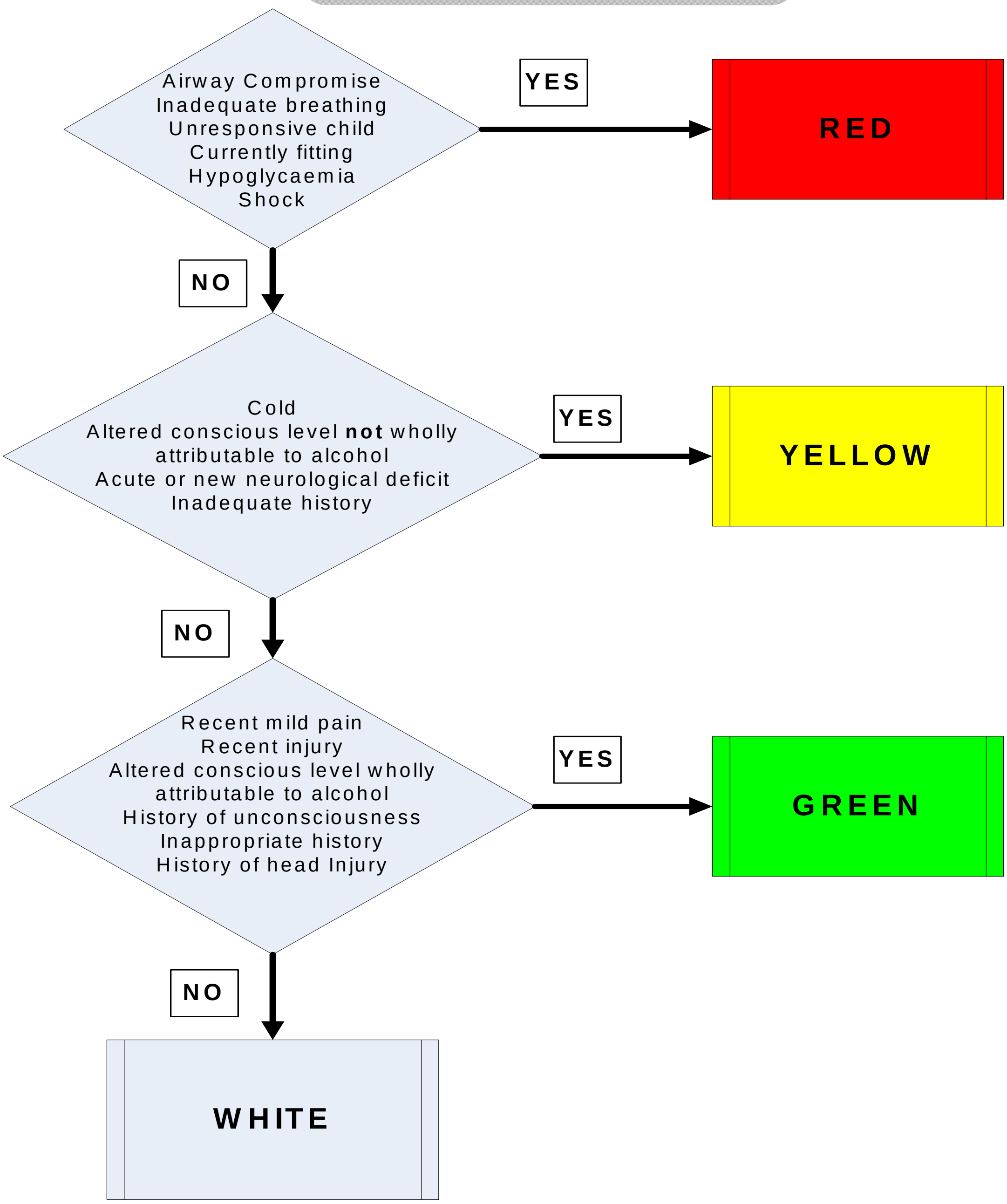

See also:	Chart notes:
Behaving strangely<br>Head injury<br>Collapsed adult	This is a presentation defined flow diagram. Large numbers of patients attend for emergency treatment in an apparently drunken state. This chart implicitly recognises that not all these patients are drunk and is designed to ensure accurate identification and prioritisation of patients who are suffering from conditions which make them appear drunk, or from such severe drunkenness that their life is threatened. A number of general discriminators have been used including Life Threat, Conscious Level in Children and Blood Glucose Level. A minor modification has been made to Conscious Level in Adult discriminator to ensure that only those adults who are unresponsive are placed into the urgent category. However a specific discriminator is included to ensure that patients in which there is an inadequate history of alcohol ingestion are seen rapidly and treated. If there is any doubt then the patient should be seen very urgently.

Assault

Orotta TS - 01 June 2007 - V. 1.1

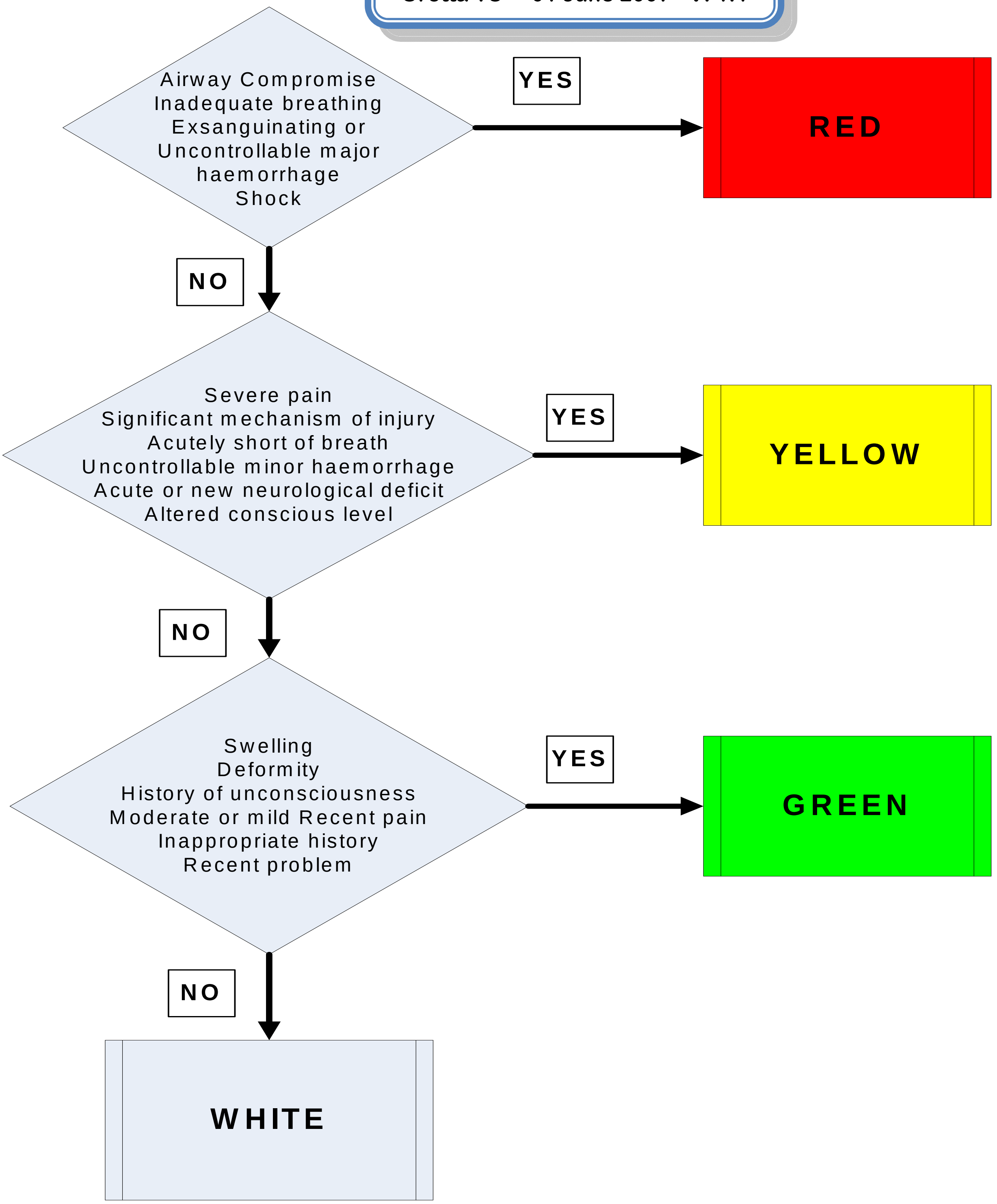

See also:	Chart notes:
Head Injury<br>Torso Injury<br>Wounds	This is a presentation defined flow diagram. Assault is a common presentation, and patients with non specific conditions following assault may be triaged using this chart. Patients who have specific injuries are better triaged using the charts which pertain to those injuries. A number of general discriminators are used including Life Threat, Haemorrhage and Pain. Specific discriminators are included to identify patients who have a significant history of injury which may indicate a more urgent requirement for treatment.

Asthma

Orotta TS - 01 June 2007 - V. 1.1

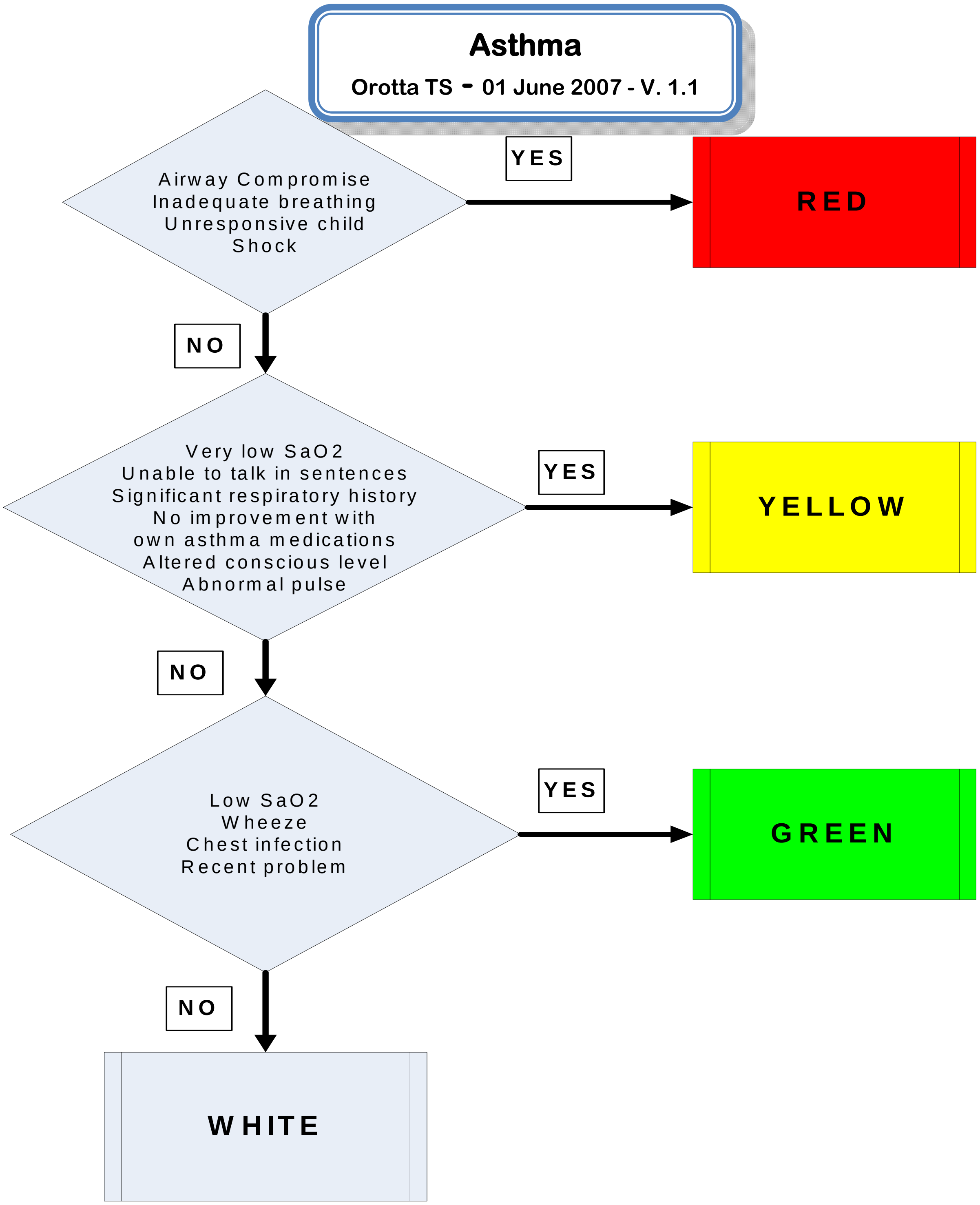

See also:	Chart notes:
Shortness of breath in Adults<br>Shortness of breath in children<br>Allergy	This is a presentation defined flow diagram which is intended for use in patients who present with the symptoms and signs of known asthma. The severity of asthmatic patients at presentation varies from those whose lives are threatened to those requiring a repeat prescription of inhalers. A number of general discriminators are used including Life Threat, Conscious Level (in adults and children) and Oxygen Saturation. Specific discriminators are included to indicate those signs and symptoms which indicate severe and life threatening asthma.

Back pain

Orotta TS - 01 June 2007 - V. 1.1

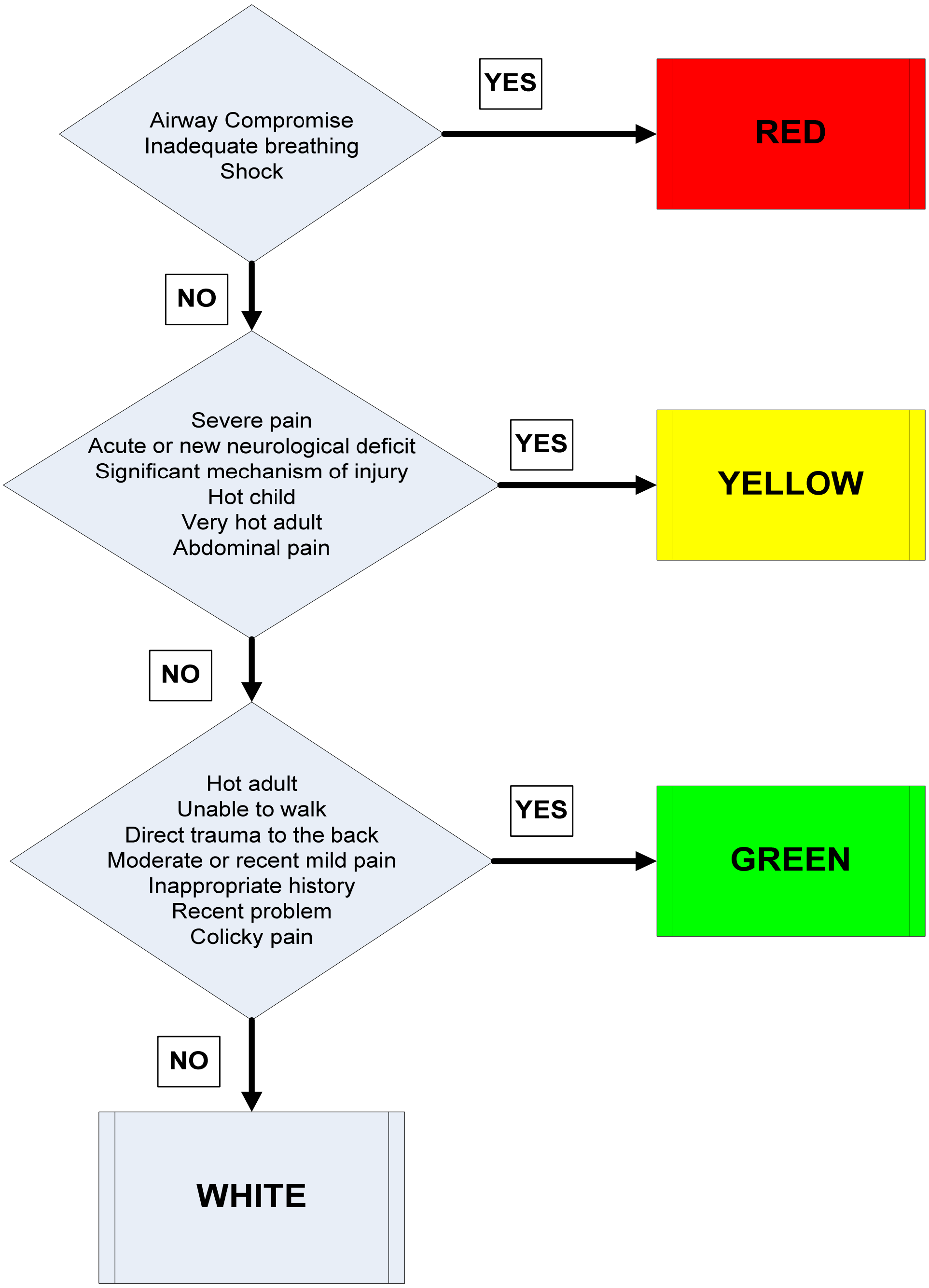

See also:	Chart notes:
Neck pain<br>Abdominal pain	This is a presentation defined flow diagram. Back pain may present to the Emergency Department either as an acute event or as an acute exacerbation of the chronic problem. A number of general discriminators are used including Life Threat, Pain and Temperature. Specific discriminators have been selected in order to allow appropriate categorisation of more urgent problems. In particular discriminators are included to allow appropriate classification of abdominal aneurysm, and patients with neurological signs and symptoms following disc prolapse.

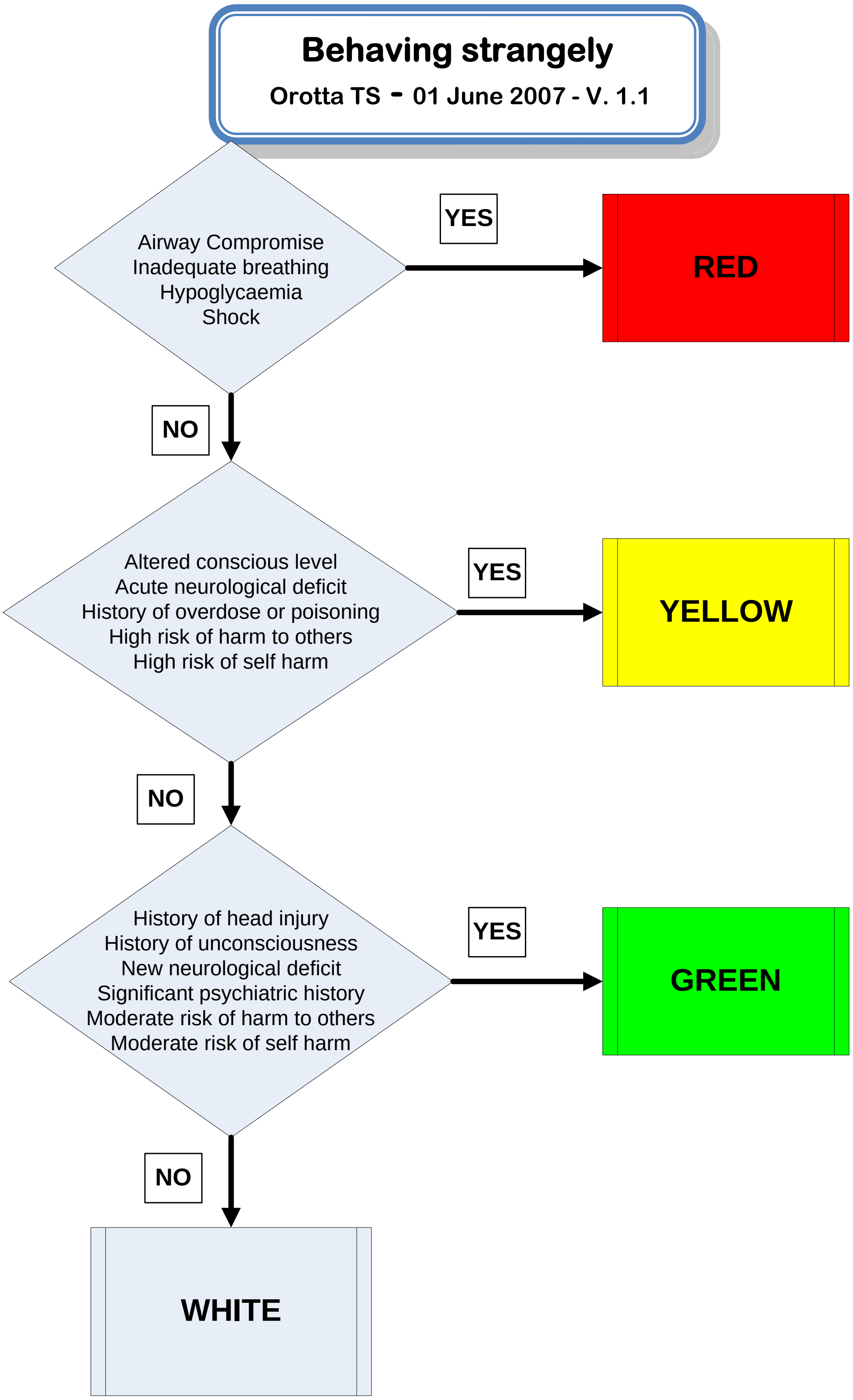

See also:	Chart notes:
Apparently drunk<br>Mental illness	This is a presentation defined flow diagram. Patients who are behaving strangely may have either a psychiatric or a physical cause for their presentation. This chart is designed to allow the accurate prioritisation of both these groups of patients. A number of general discriminators have been used including Life Threat and Conscious Level. Specific discriminators are used and in particular the concepts of risk of harm to others and risks of self harm are introduced.

Bites and stings

Orotta TS - 01 June 2007 - V. 1.1

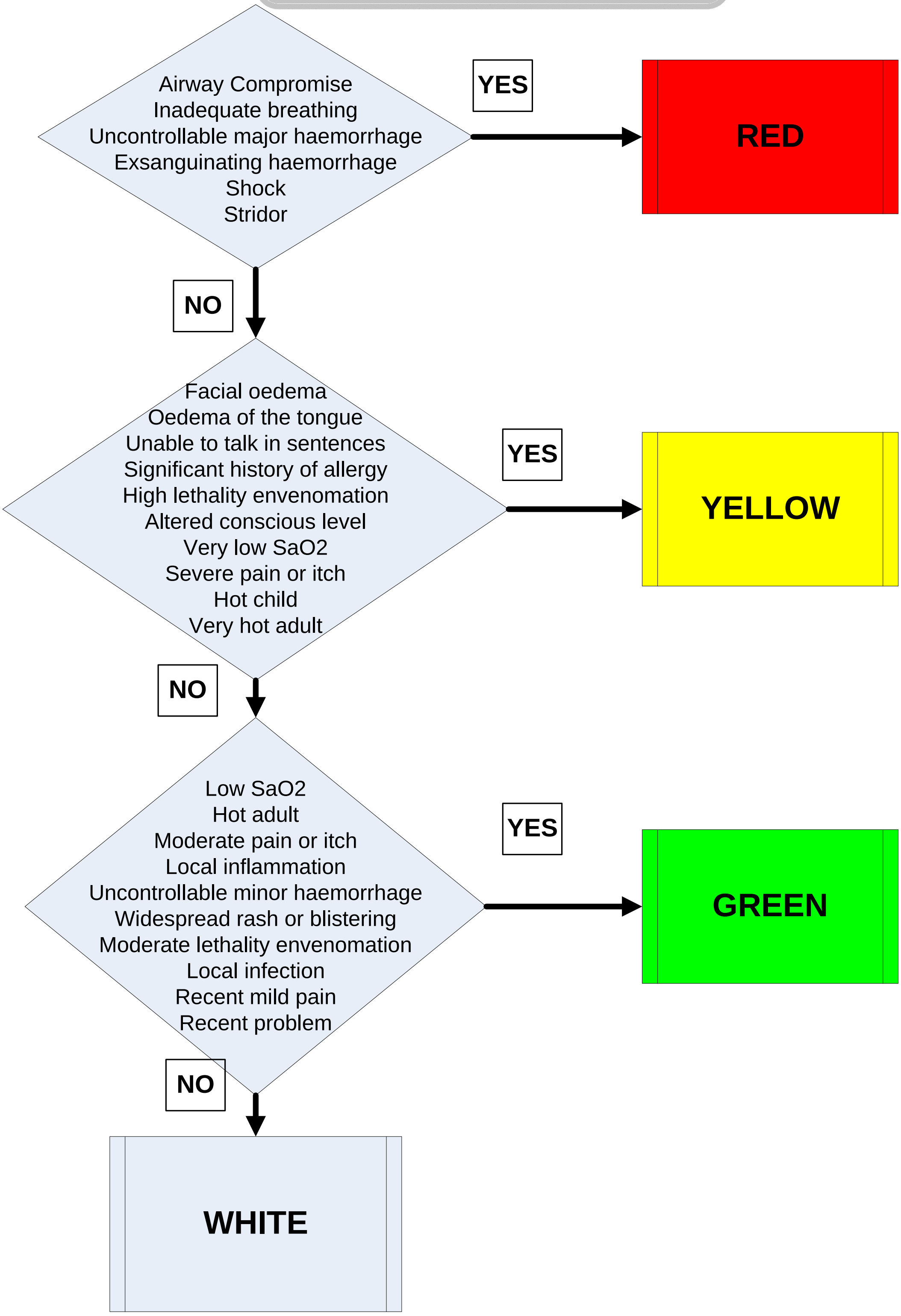

See also:	Chart notes:
Allergy, Abscesses and Local Infections	This is a presentation defined flow diagram designed to allow accurate prioritisation of patients who present following bites and stings. Bites may, of course, range from those delivered by insects to those delivered by large animals, therefore there is a complete range of priority covered by this presentation. A number of general discriminators are used including Life Threat, Haemorrhage and Pain. Specific discriminators have been added to the chart to allow accurate identification of patients requiring more urgent treatment because of more severe injury or the development of allergic reactions.

Burns and scalds

Orotta TS - 01 June 2007 - V. 1.1

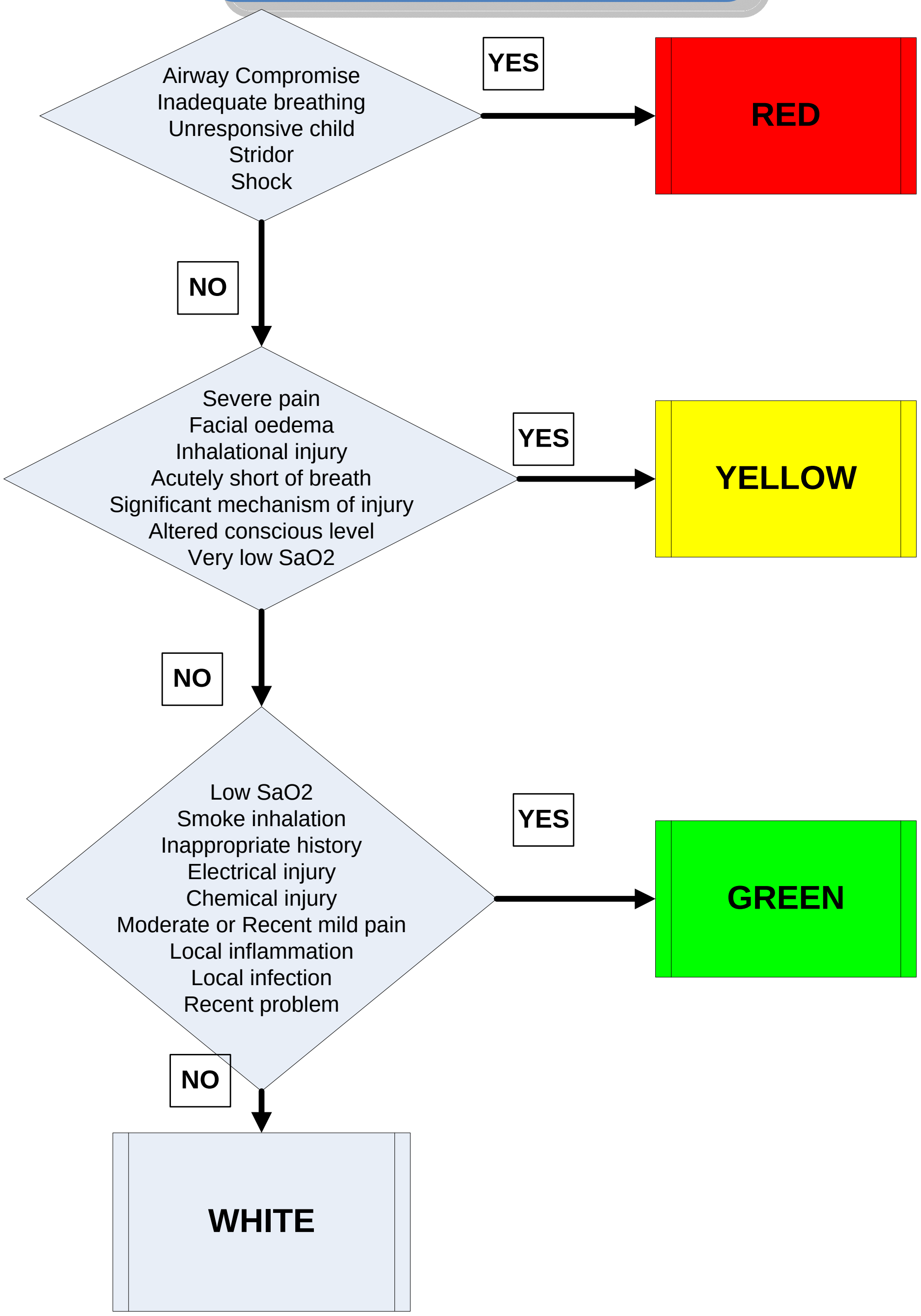

See also:	Chart notes:
	This is a presentation defined flow diagram. There is a complete range of severity with this presentation and the chart has been designed to allow accurate identification of patients within each category. A number of general discriminators are used including Life Threat, Conscious Level and Pain. Specific discriminators have been added to allow identification of patients who have suffered inhalation injury, and those in whom the mechanism suggests that further investigation and treatment may be appropriate.

Chest Pain

Orotta TS - 01 June 2007 - V. 1.1

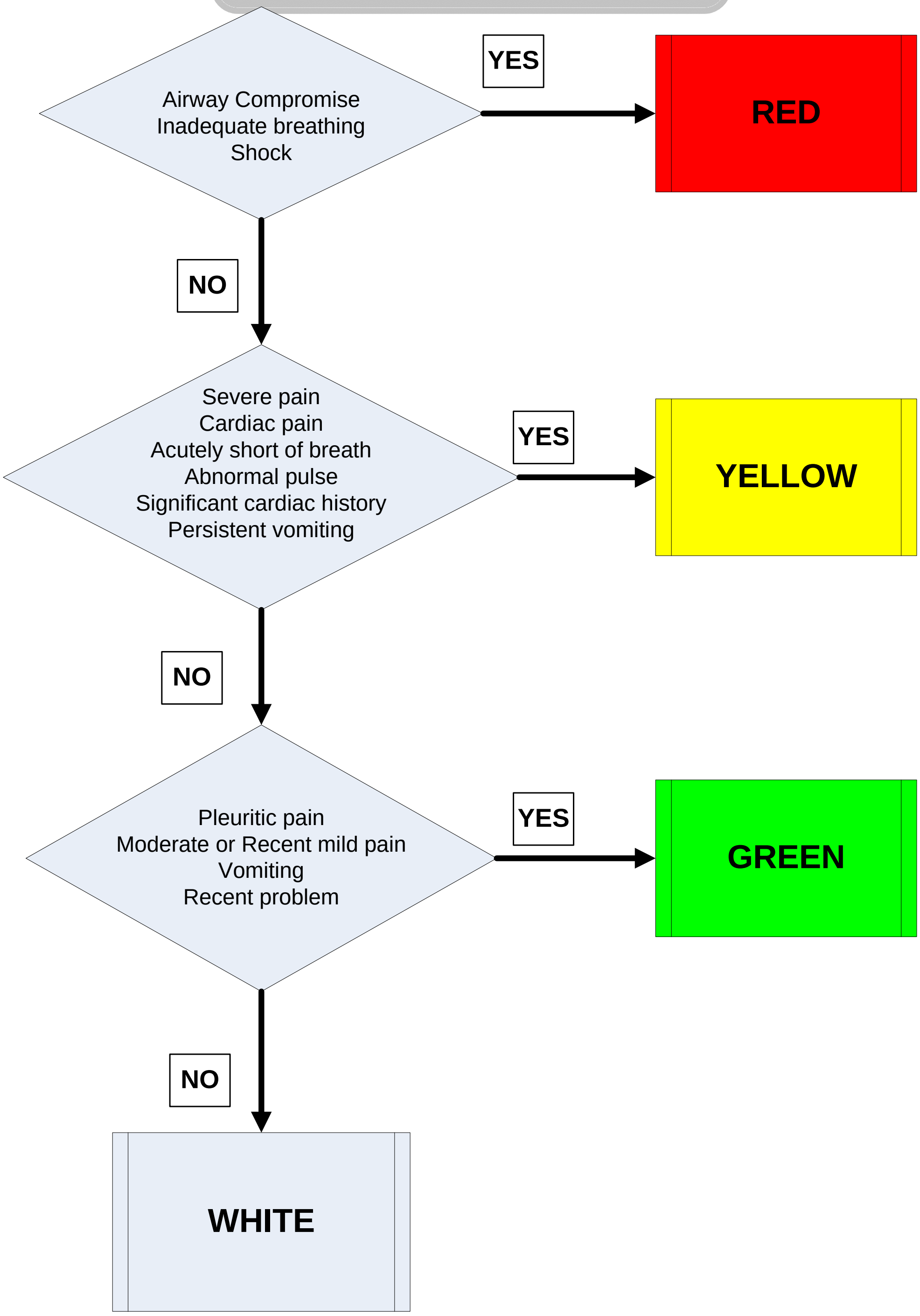

See also:	Chart notes:
	This is a presentation defined flow diagram. Chest pain is a common presentation to Emergency Departments forming some 2-5% of all patient contacts. Causes of chest pain may vary from acute myocardial infraction to muscular irritation, and appropriate categorisation is paramount. A number of general discriminators are used including Life Threat and Pain. Specific discriminators include the nature and severity of pain (cardiac or pleuritic) and abnormalities of pulse.

Collapsed Adult

Orotta TS - 01 June 2007 - V. 1.1

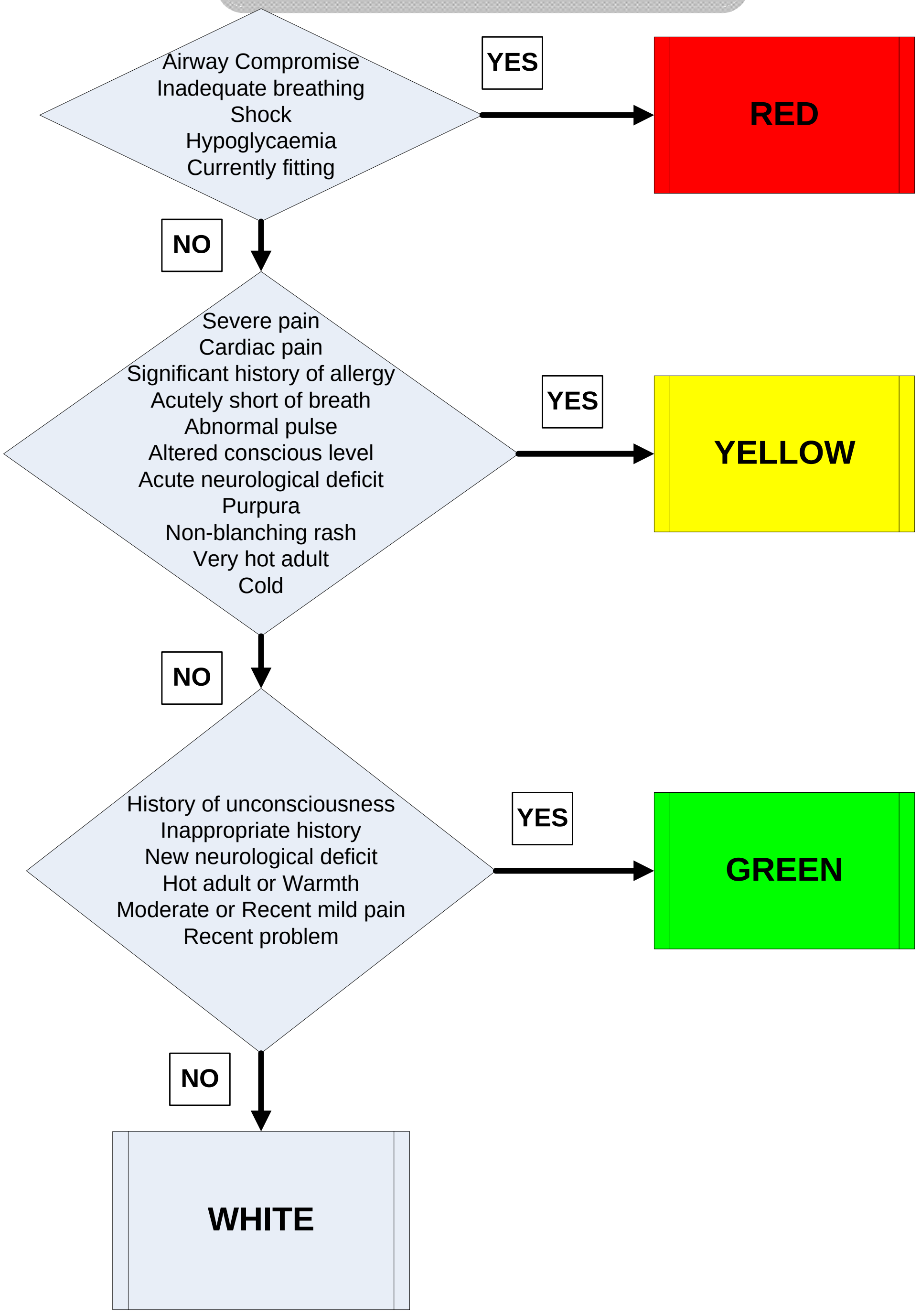

See also:	Chart notes:
Fits<br>Unwell<br>Apparently drunk	This is a presentation defined flow diagram. Presentation with Collapse is not uncommon in an emergency department and this chart is designed to allow rapid triage of patients who present in this way. A number of general discriminators are used including life threat, conscious level, pain, and temperature. Specific discriminators have been added to the chart to try and rule out more serious pathology. As with all charts those pathologies (such as myocardial infarction) which can potentially benefit from early intervention are deliberately categorised highly.

Diabetes

Orotta TS - 01 June 2007 - V. 1.1

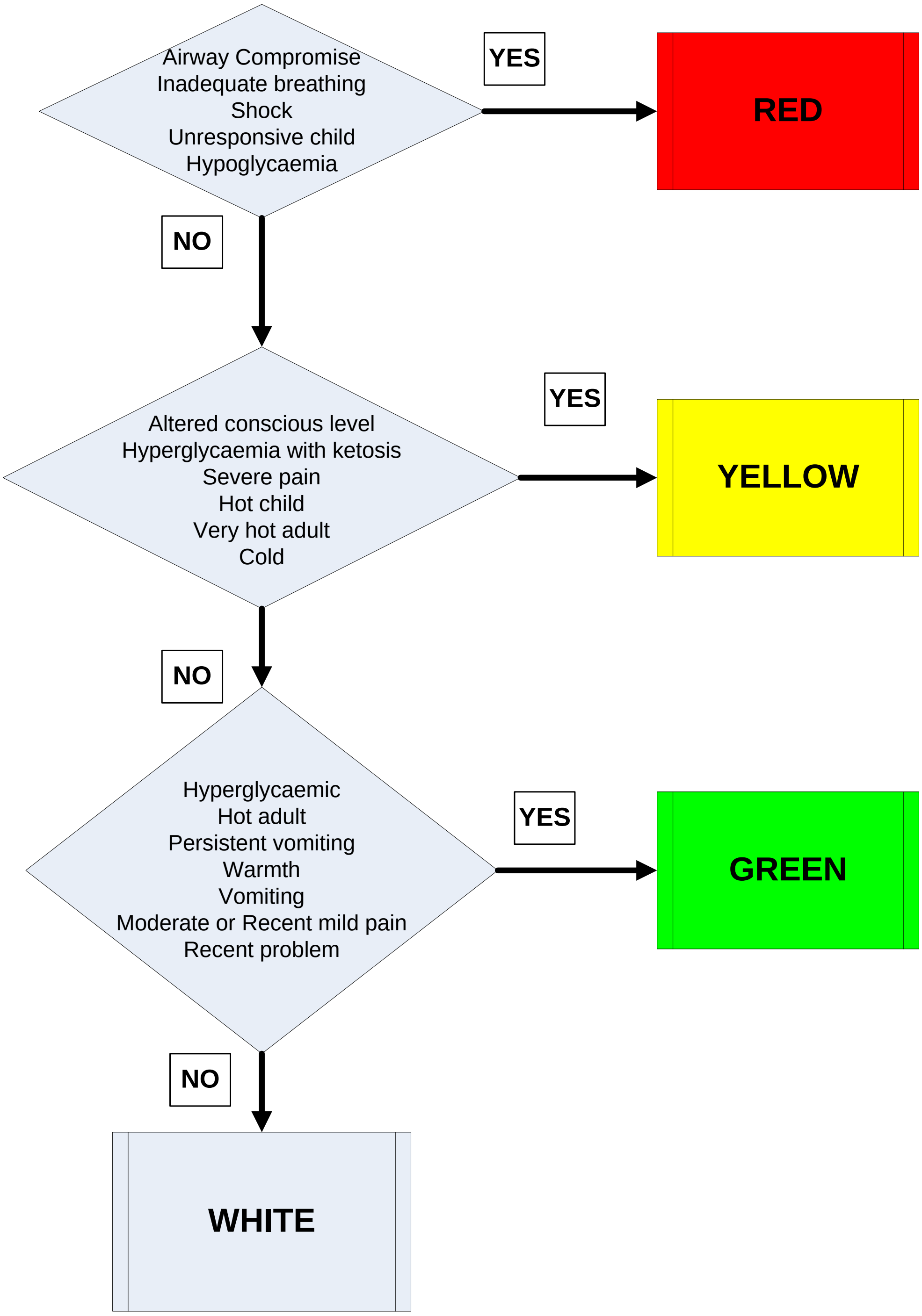

See also:	Chart notes:
	This is a presentation defined flow diagram designed to allow categorisation of patients who present with known cases of diabetes. A number of general discriminators are used including Life Threat, Conscious Level (both adult and child), Blood Glucose Level and Temperature.

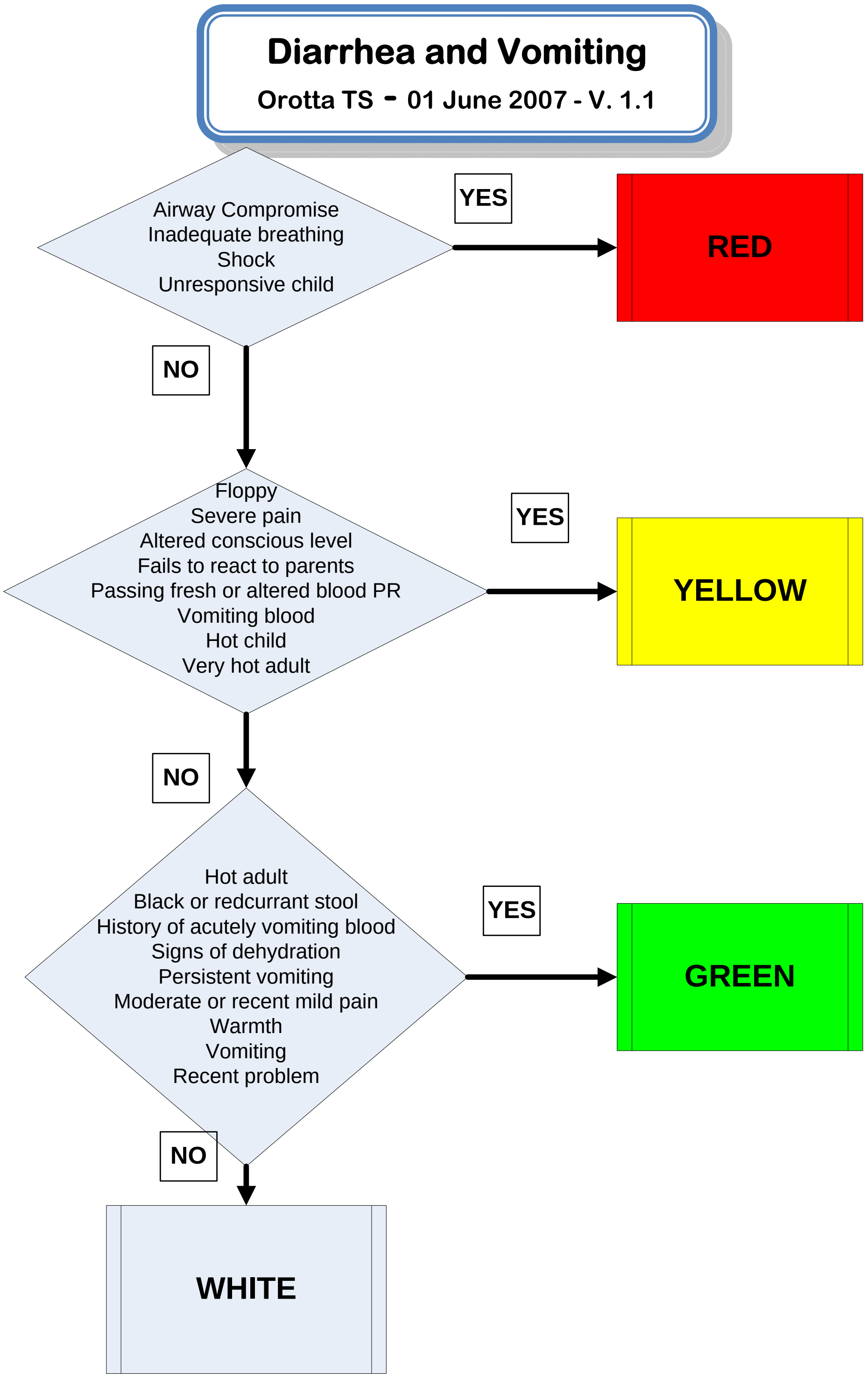

See also:	Chart notes:
GI bleeding<br>Abdominal Pain in adults, Abdominal Pain in Children	This is a new presentation defined flow diagram, combining the previous diarrhoea and vomiting charts. Most patients who present with diarrhoea or vomiting do not have high priority. However a number may have serious underlying pathology. A number of general discriminators are used including Life Threat and Pain. Specific discriminators have been included to ensure that patients suffering from GI bleeding, and those with dehydration and other severe effect of diarrhoea and vomiting are included in the appropriate categories.

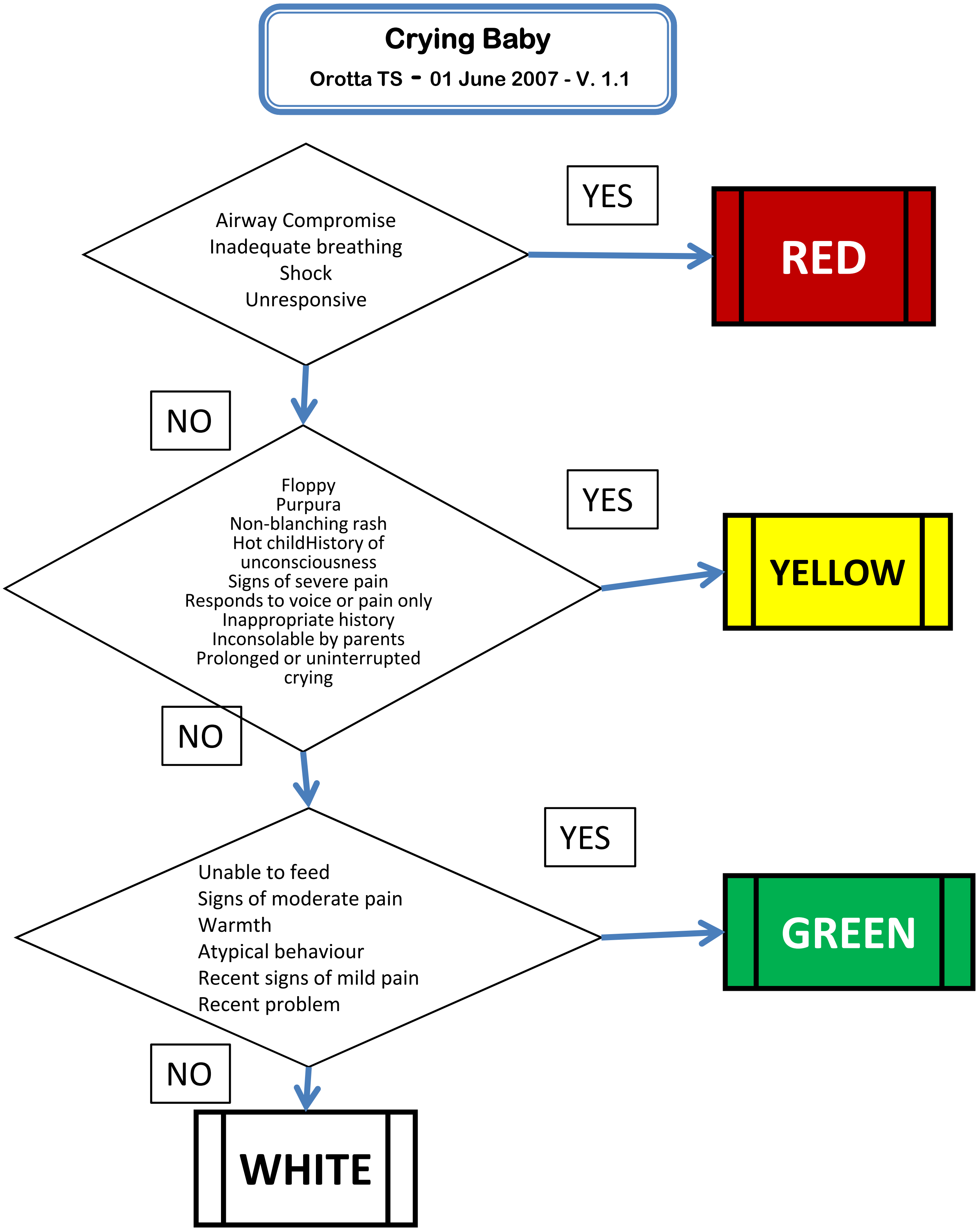

**See also:**	**Chart notes:**
Unwell child	This is a presentation defined flow diagram. This chart has been designed to allow accurate prioritisation of children who are presented by their parents with a chief complaint of crying. A number of general discriminators have been used including Life Threat, Conscious Level and Pain. Specific discriminators include those which allow recognition of more specific pathologies such as septicaemia, or which indicate that a more serious pathology might exist. No children can be categorised as GREEN or WHITE until all the specific and general discriminator outlined under the RED and YELLOW categories have been specifically excluded. This may take longer than the time available for initial assessment.

Dental Problems

Orotta TS - 01 June 2007 - V. 1.1

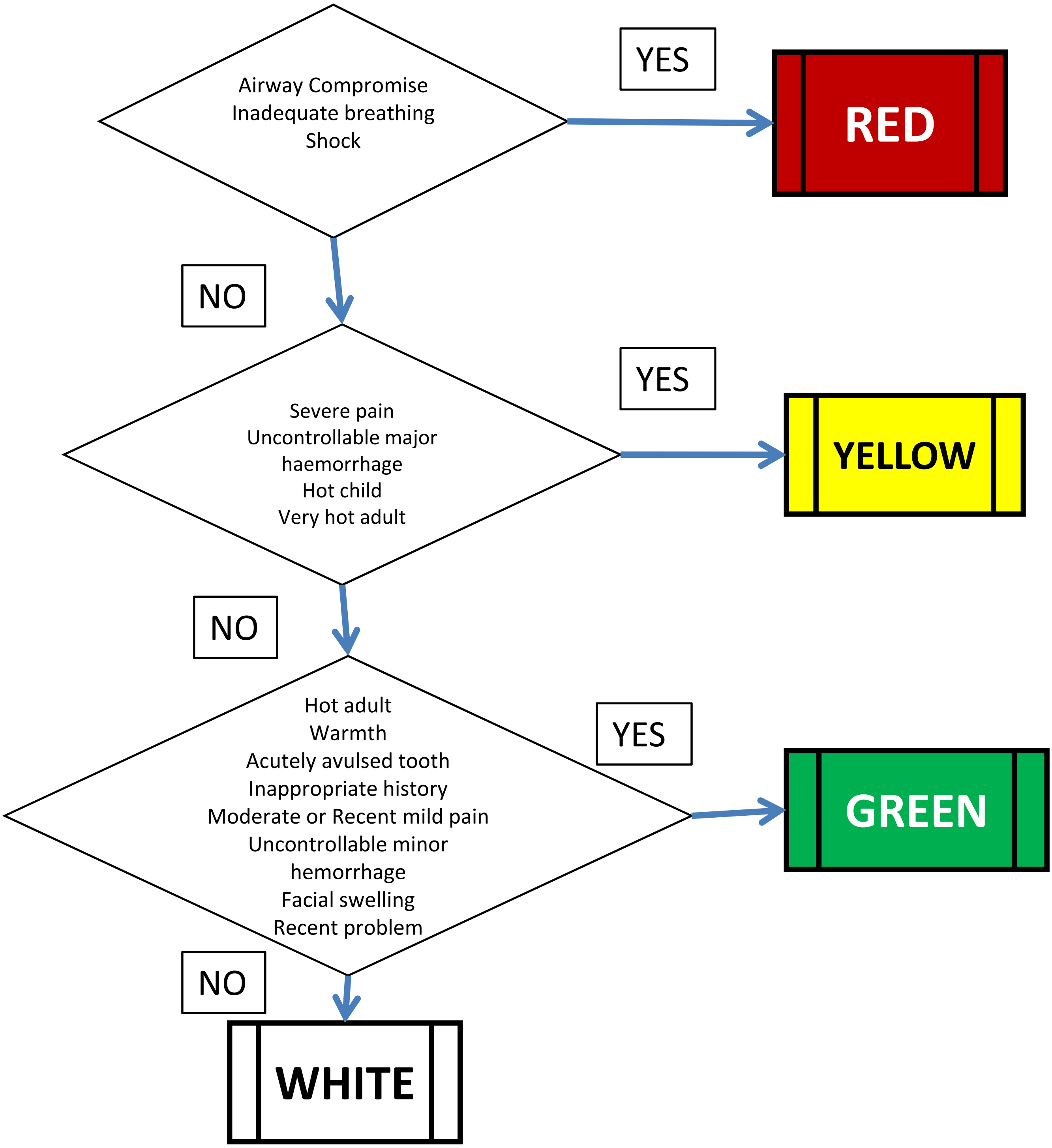

**See also:**	**Chart notes:**
Facial Problems	This is a presentation defined flow diagram designed to allow accurate prioritisation of patients presenting problems affecting the teeth or gums. A number of general discriminators have been used including Life Threat, Pain, Haemorrhage and Temperature. Acute avulsion of a tooth has been included in the urgent. It is important to ensure that preconceptions about disposal do not affect accurate triage of patients with these presentations.

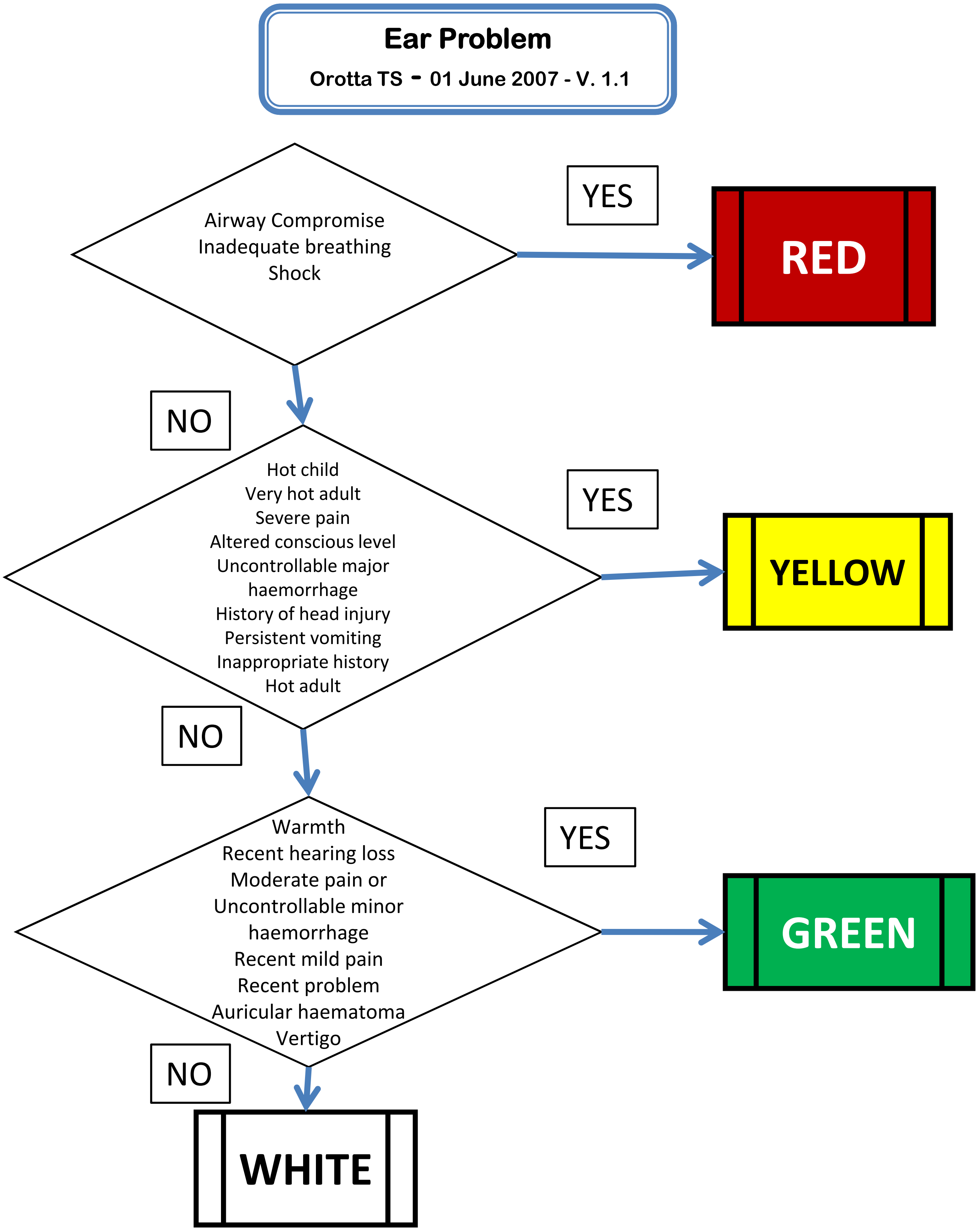

See also:	Chart notes:
Facial Problems<br>Head injury	This is a presentation defined flow diagram designed to allow accurate prioritisation of patients presenting with conditions affecting the ear. A number of general discriminators are used including *Life Threat, Pain, Hemorrhage and Temperature*.

Exposure to Chemicals

Orotta TS - 01 June 2007 - V. 1.1

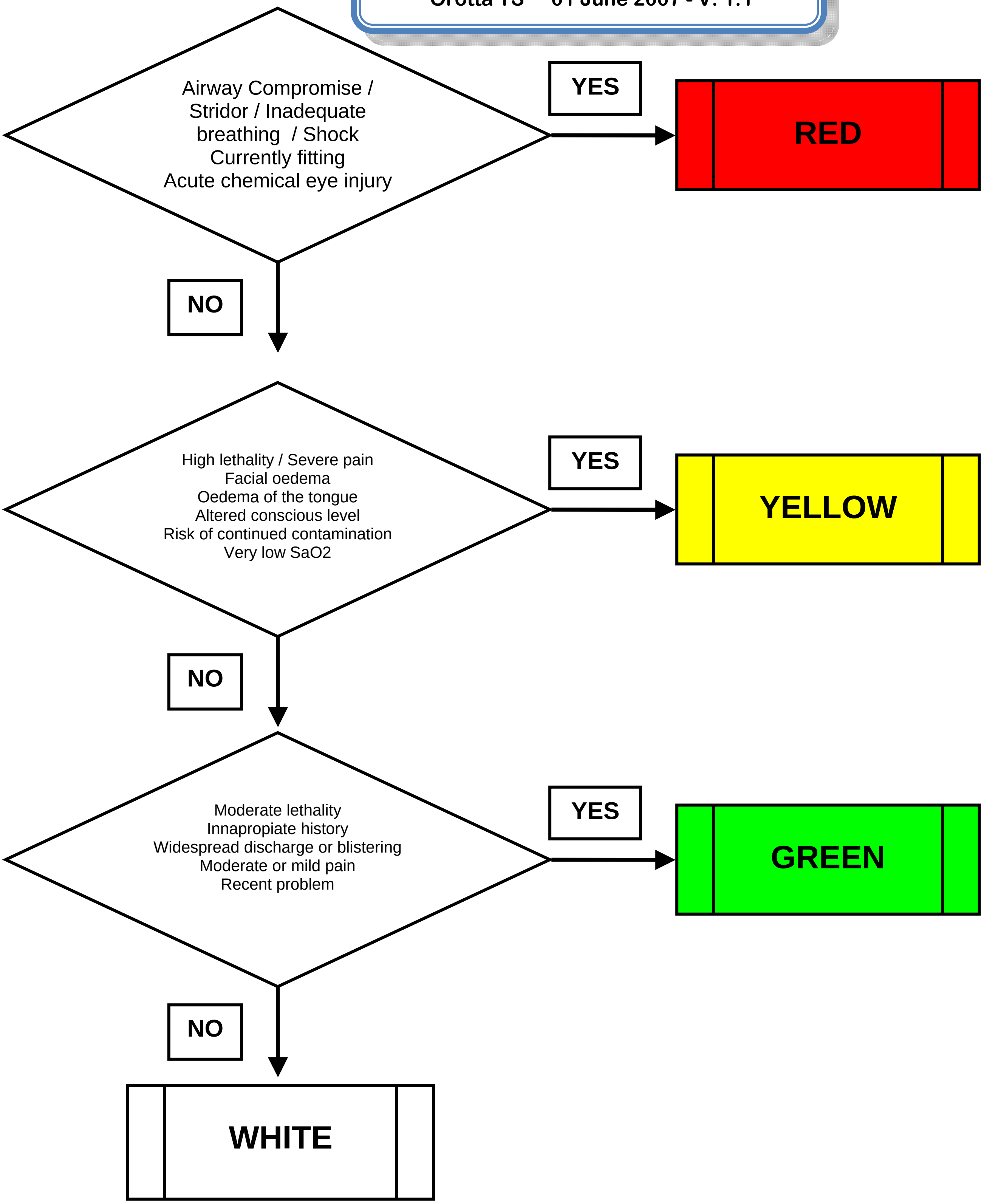

**See also:**	**Chart notes:**
Shortness of breath<br>Overdoses and poisoning	This is a presentation defined flow diagram. While this presentation is not common it is important because it is often the chief complaint of the patient. The signs and symptoms do not necessarily fit easily into any other presentational group. A number of general discriminators are used including *Life Threat, Conscious Level, Pain and Oxygen Saturation*. Specific discriminators which include those for the shortness of breath have been added to appropriate categories. *Acute Chemical Eye Injury* appears in the RED category and *Risk of continued contamination* appears in the YELLOW

Eye problems

Orotta TS - 01 June 2007 - V. 1.1

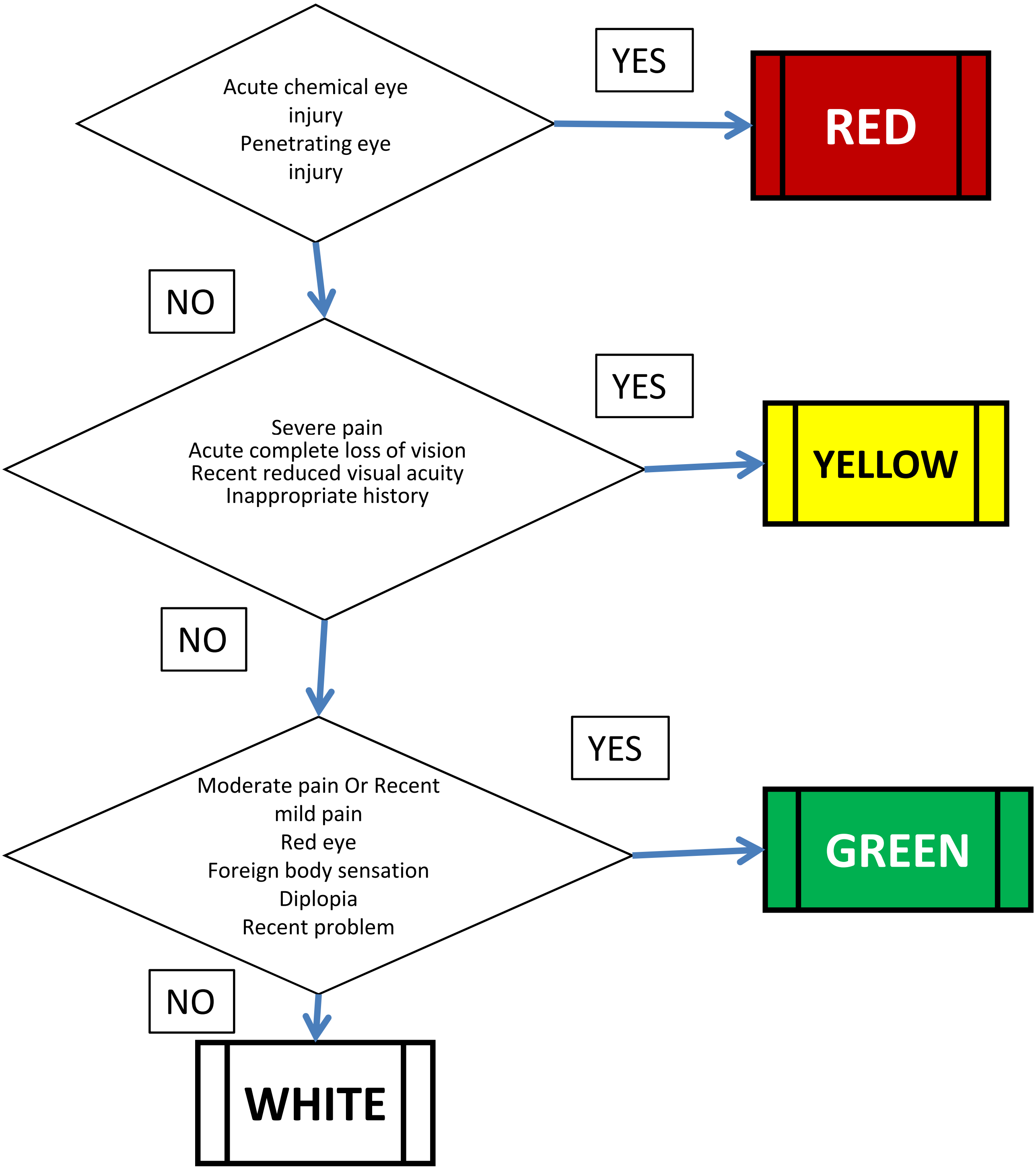

**See also:**	**Chart notes:**
Facial Problems	This is a presentation defined flow diagram designed to allow accurate priorisation of patients attending with conditions affecting the eye. Pain is used as a general discriminator. A number of specific discriminators have been used including a history of acute chemical injury, which indicates that immediate action is require, a history of penetrating eye injury or sudden or acute complete loss of vision and an assessment of visual acuity.

Facial Problems

Orotta TS - 01 June 2007 - V. 1.1

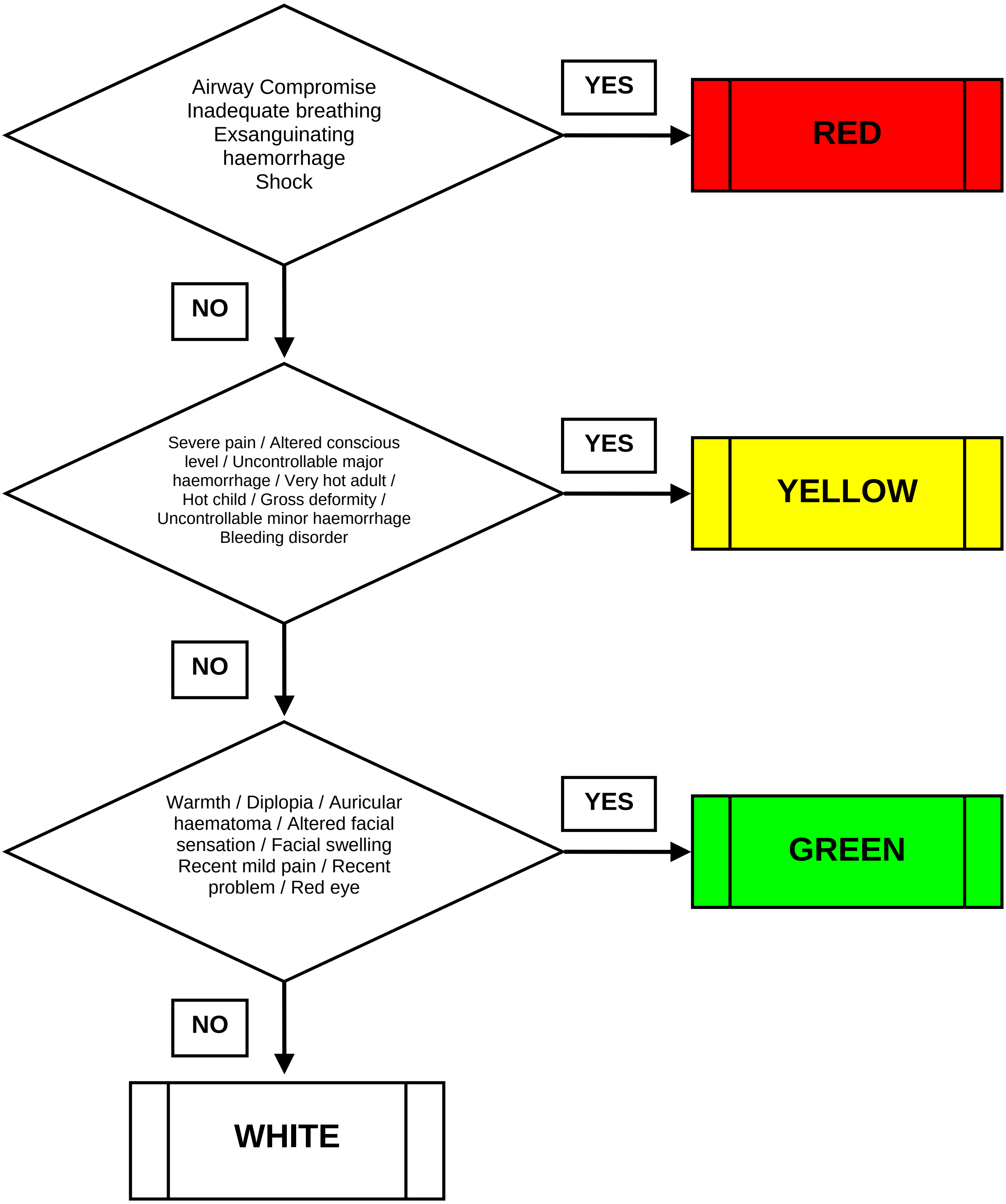

See also:	Chart notes:
Dental Problems<br>Ear Problems<br>Eye Problems<br>Head Injury	This is a new presentation defined flow diagram which supersed the nasal problems chart. It has been designed to allow accurate prioritisation of patients attending with problems affecting the face. A number of general discriminators have been used including *Life Threat, Haemorrhage and Pain*.

Falls

Orotta TS - 01 June 2007 - V. 1.1

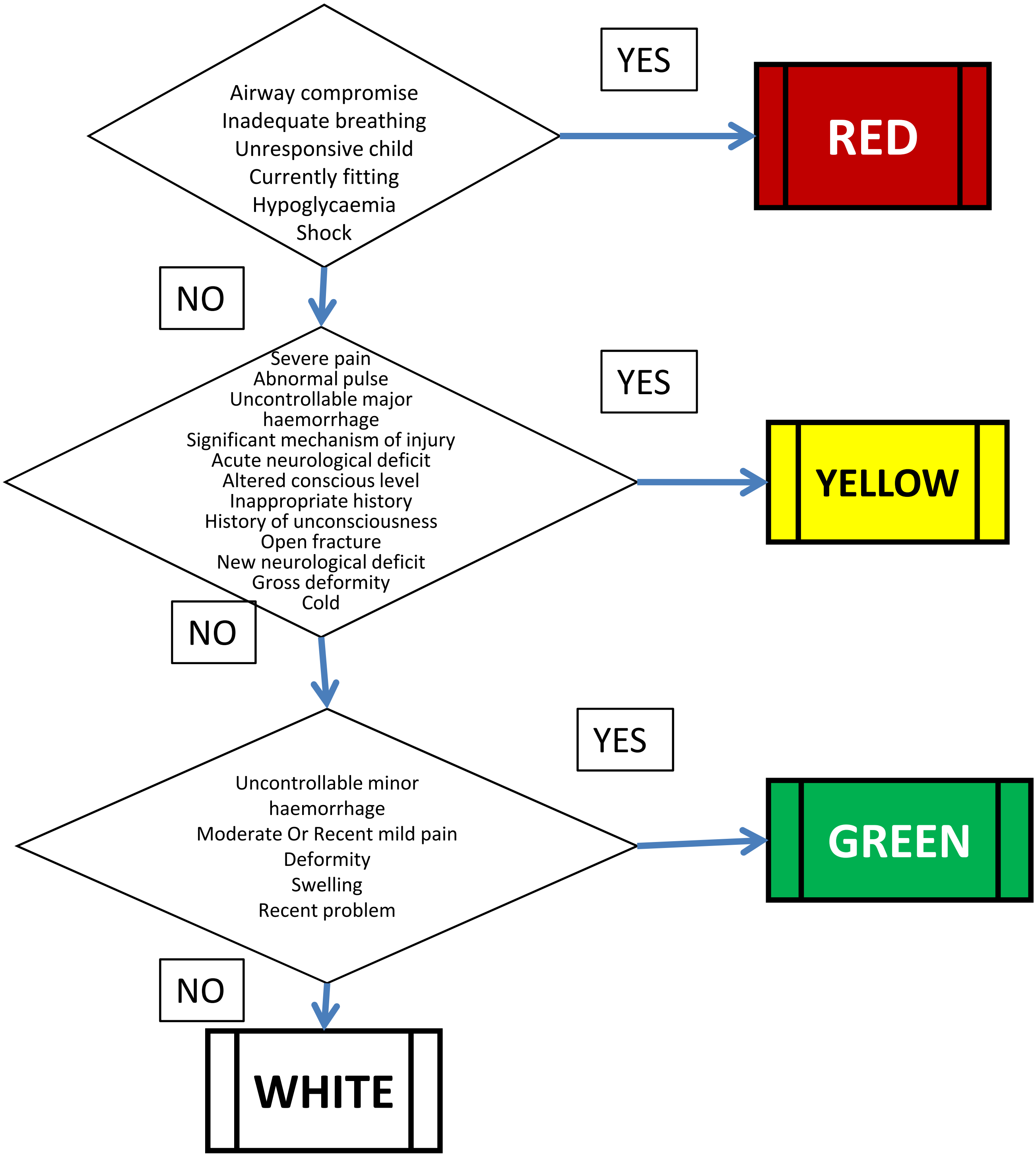

See also:	Chart notes:
Collapsed adult	This is a presentation defined flow diagram. Many patients who present with a history of falls have suffered trauma as a result, and their priority will reflect the injuries suffered. Some, however, may have a serious underlying pathology which has caused them to fall, or may have developed complications after falling. This chart is designed to allow accurate prioritisation whether the injury or underlying cause is more pressing. A number of general discriminators have been included to ensure that patients suffering from serious underlying conditions or limb threatening injuries are given a high priority.

Fits (convulsions)

Orotta TS - 01 June 2007 - V. 1.1

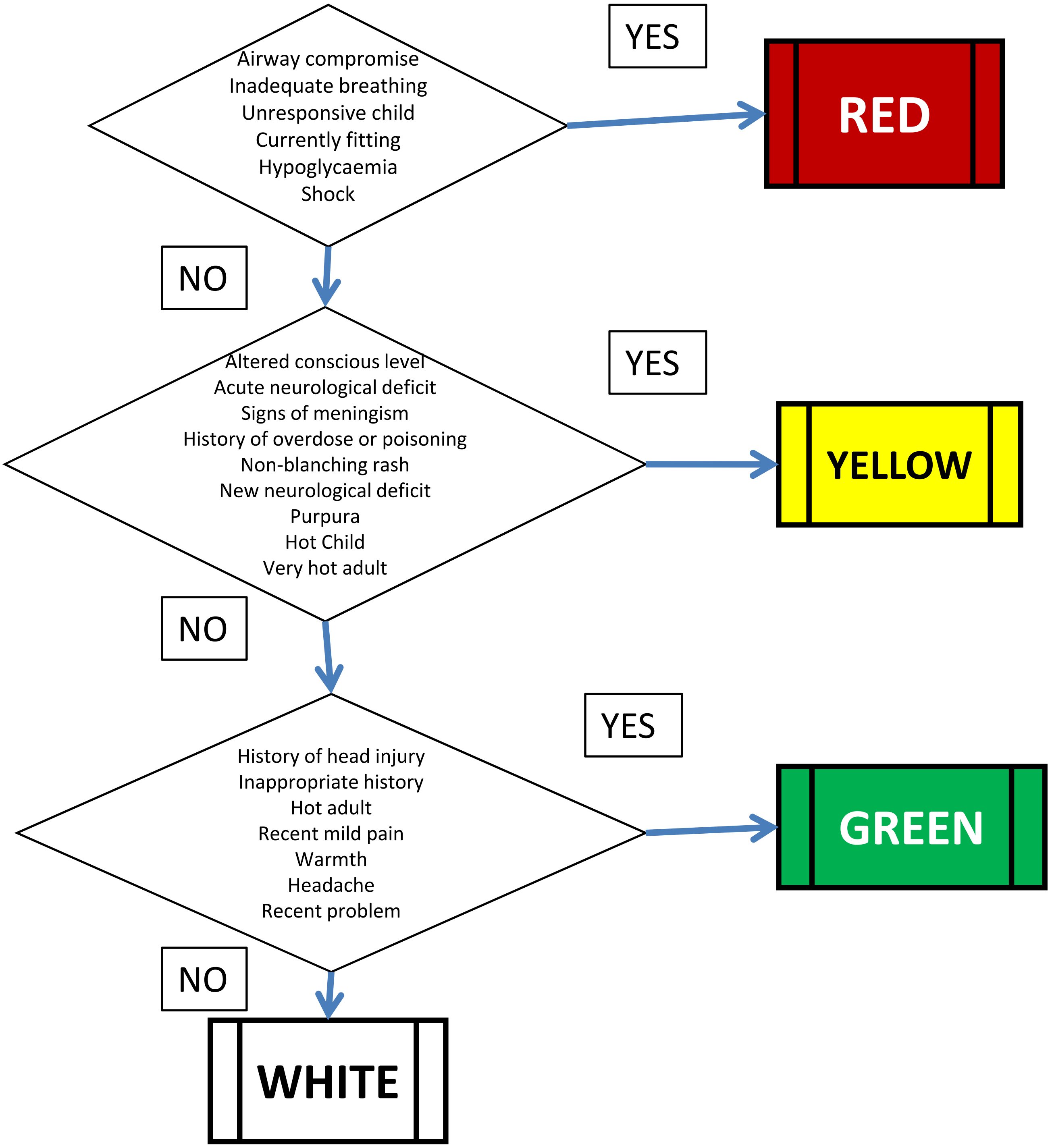

**See also:**	**Chart notes:**
Head injury<br>Headache<br>Overdoses and poisoning	This is a presentation defined flow diagram. It is not an uncommon presentation to the Emergency Department and this chart is designed to allow rapid categorisation of patients who are currently fitting or who have fitted. A number of general discriminators are used including life threat, conscious level, and temperature. Specific discriminators include signs of meningism and a focal or progressive loss of function. As with all unconscious patients rapid blood sugar estimation would be indicated to exclude hypoglycaemia.

Foreign Body

Orotta TS - 01 June 2007 - V. 1.1

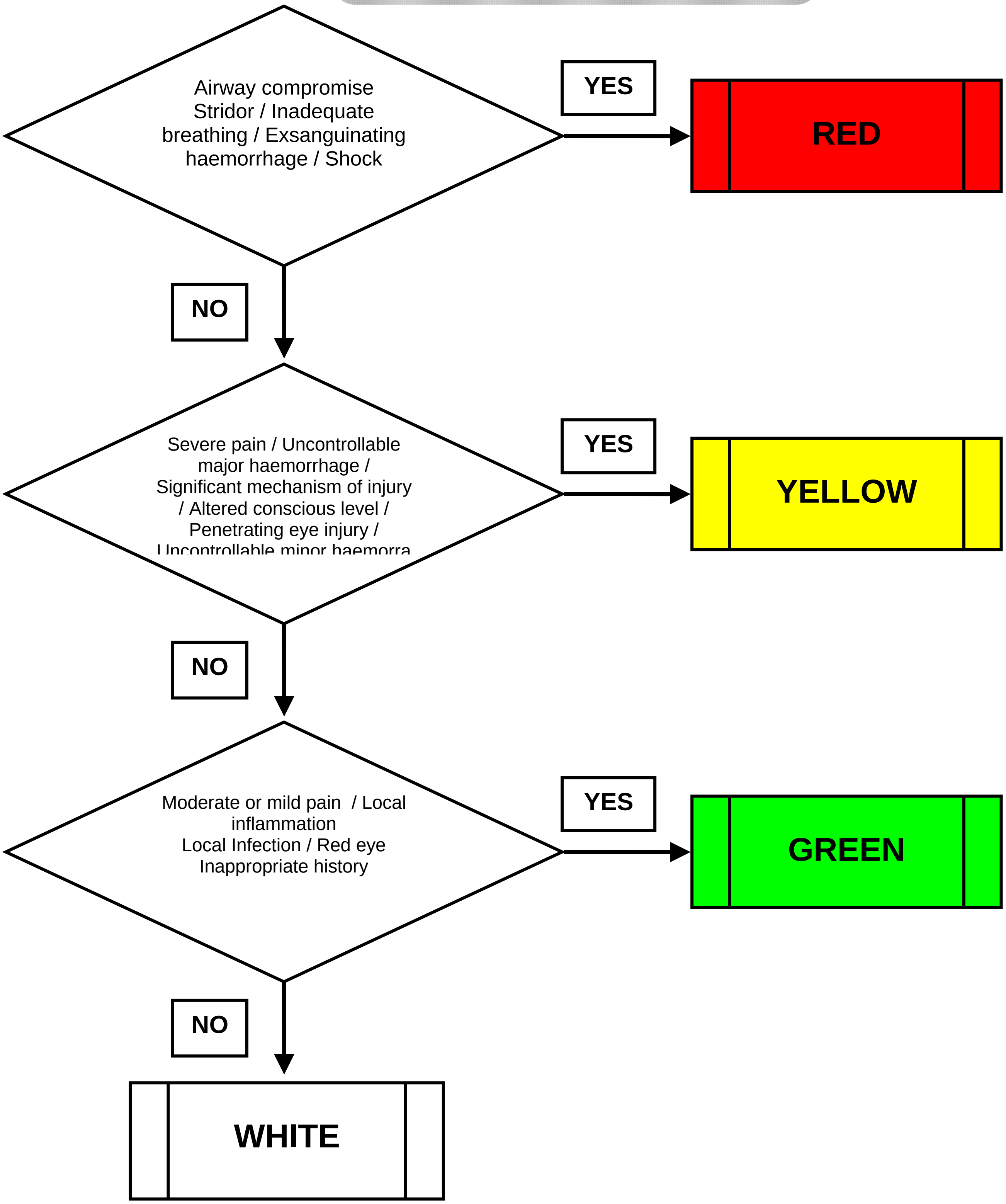

**See also:**	**Chart notes:**
Wounds<br>Torso Injury	This is a presentation defined flow diagram designed to allow accurate prioritisation of patients who present with foreign bodies in any part of their anatomy. The severity of such cases can range from the inconvenient to the life threatening and this chart is designed to differentiate between these. A number of general discriminators have been used including *Life Threat, Haemorrhage and Pain*. The only specific discriminator that relates to anatomical site is that of eye penetration.

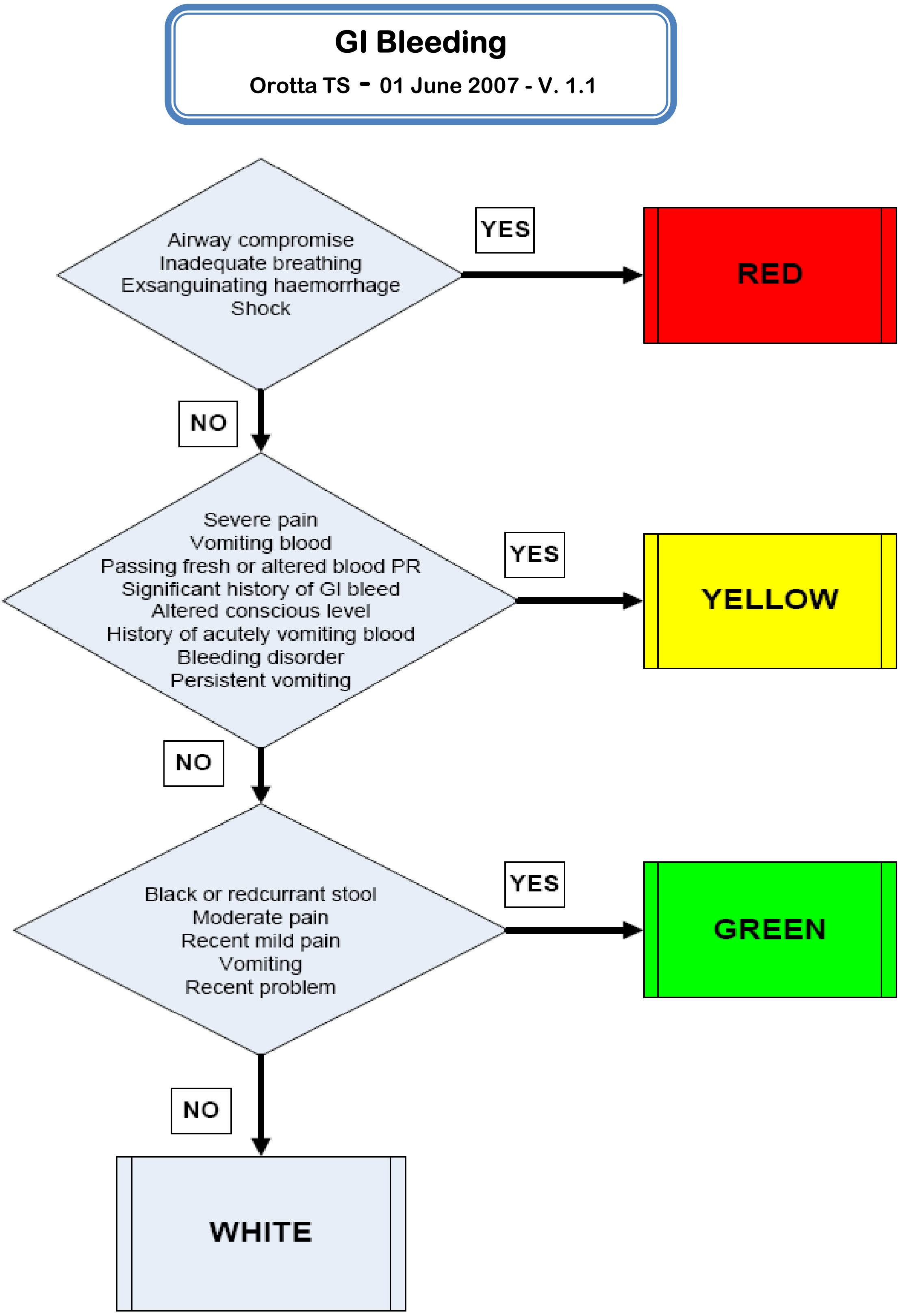

See also:	Chart notes:
Diarrhoea and Vomiting, Abdominal Pain in Adults, Abdominal Pain in Children	This is a presentation defined flow diagram. Patients may present with GI Bleeding either as vomiting altered or unaltered blood, or by passing blood PR. A number of general discriminators are used including Life Threat and Pain. Specific discriminators have been selected to indicate the current severity of the GI Bleeding. Thus patients vomiting blood or those passing fresh or altered blood PR (Per Rectum) have a higher category than those with a history of vomiting.

Head Injury

Orotta TS - 01 June 2007 - V. 1.1

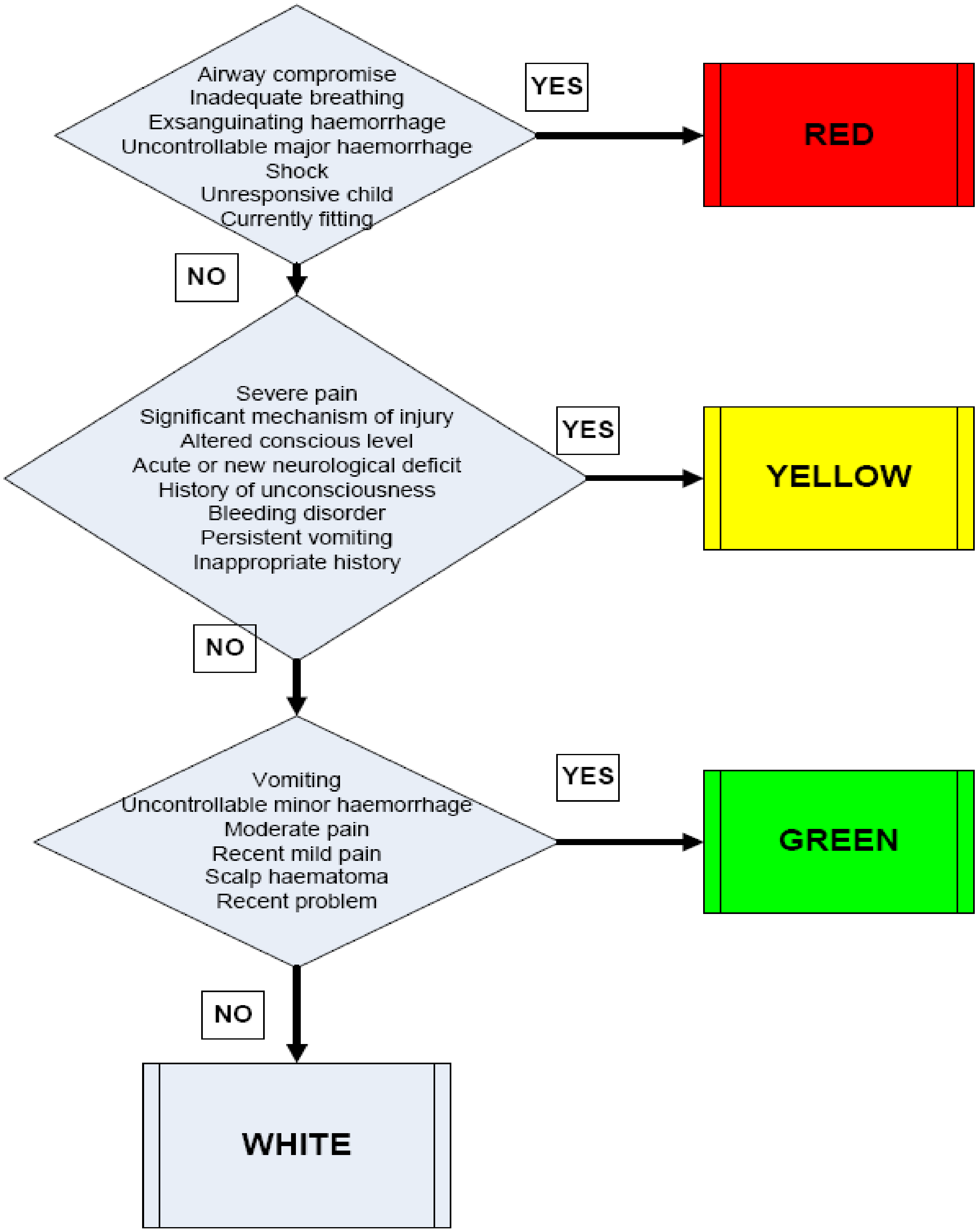

**See also:**	**Chart notes:**
Headache<br>Neck pain<br>Fits	This is a presentation defined flow diagram. Head injury is an extremely common presentation and its effects may vary from life threatening extradural haemorrhage to minimal scalp injury. A number of general discriminators have been used including Life Threat, Conscious Level (both in adults and children), Haemorrhage and Pain. Specific discriminators are included to select those patients with significant mechanism and the development of neurological signs and symptoms, to a higher priority.

Headache

Orotta TS - 01 June 2007 - V. 1.1

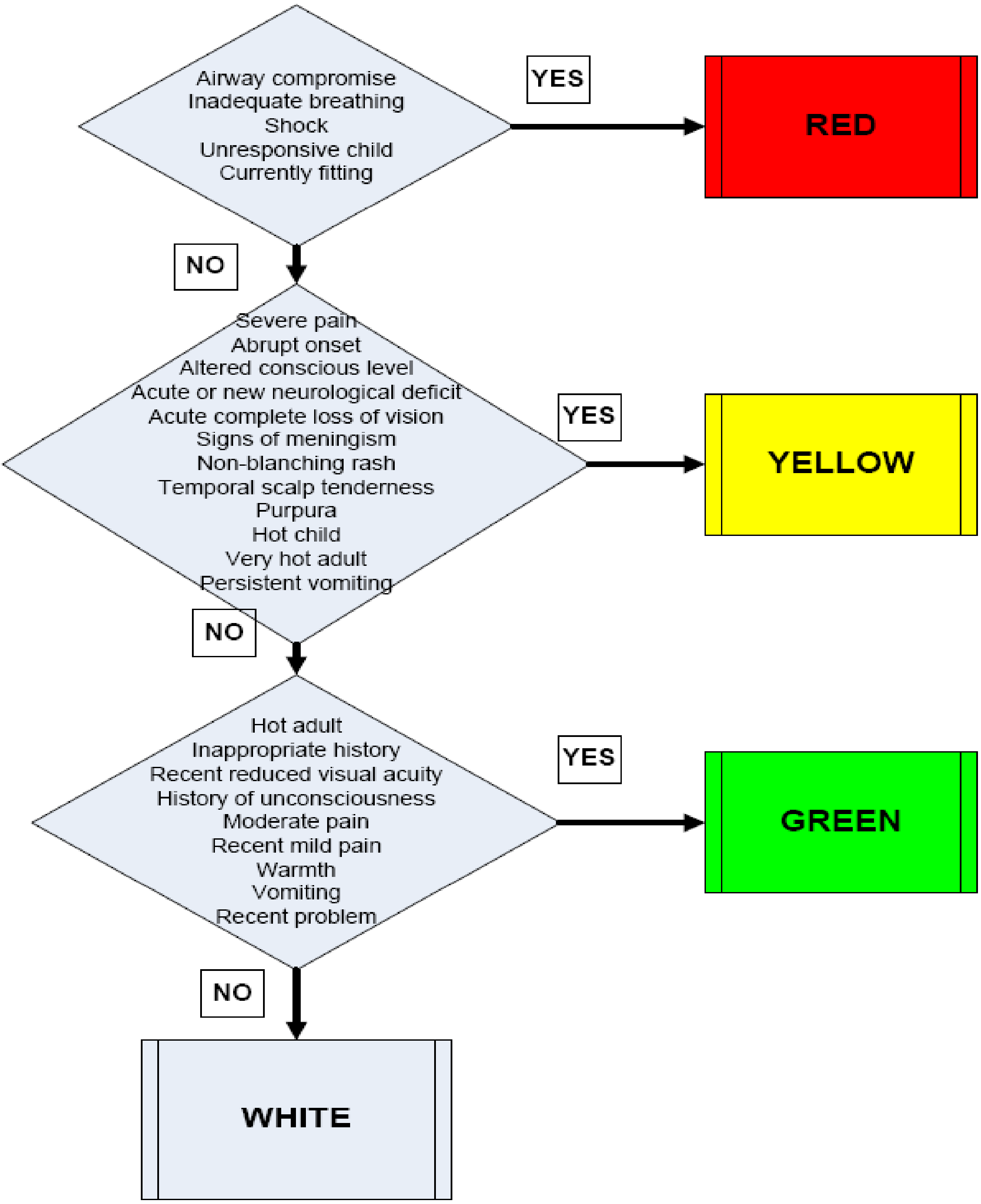

See also:	Chart notes:
Head Injury<br>Neck Pain	This is a presentation defined flow diagram. A large number of conditions can present with headache and a number of these require urgent intervention. A number of general discriminators are used including Life Threat, Conscious Level, Pain and Temperature. Specific discriminators have been used to identify severe causes such as subarachnoid haemorrhage and meningococcicemia. New neurological signs and tenderness of the scalp are used to indicate the need for urgent clinical review.

Limb Problems

Orotta TS - 01 June 2007 - V. 1.1

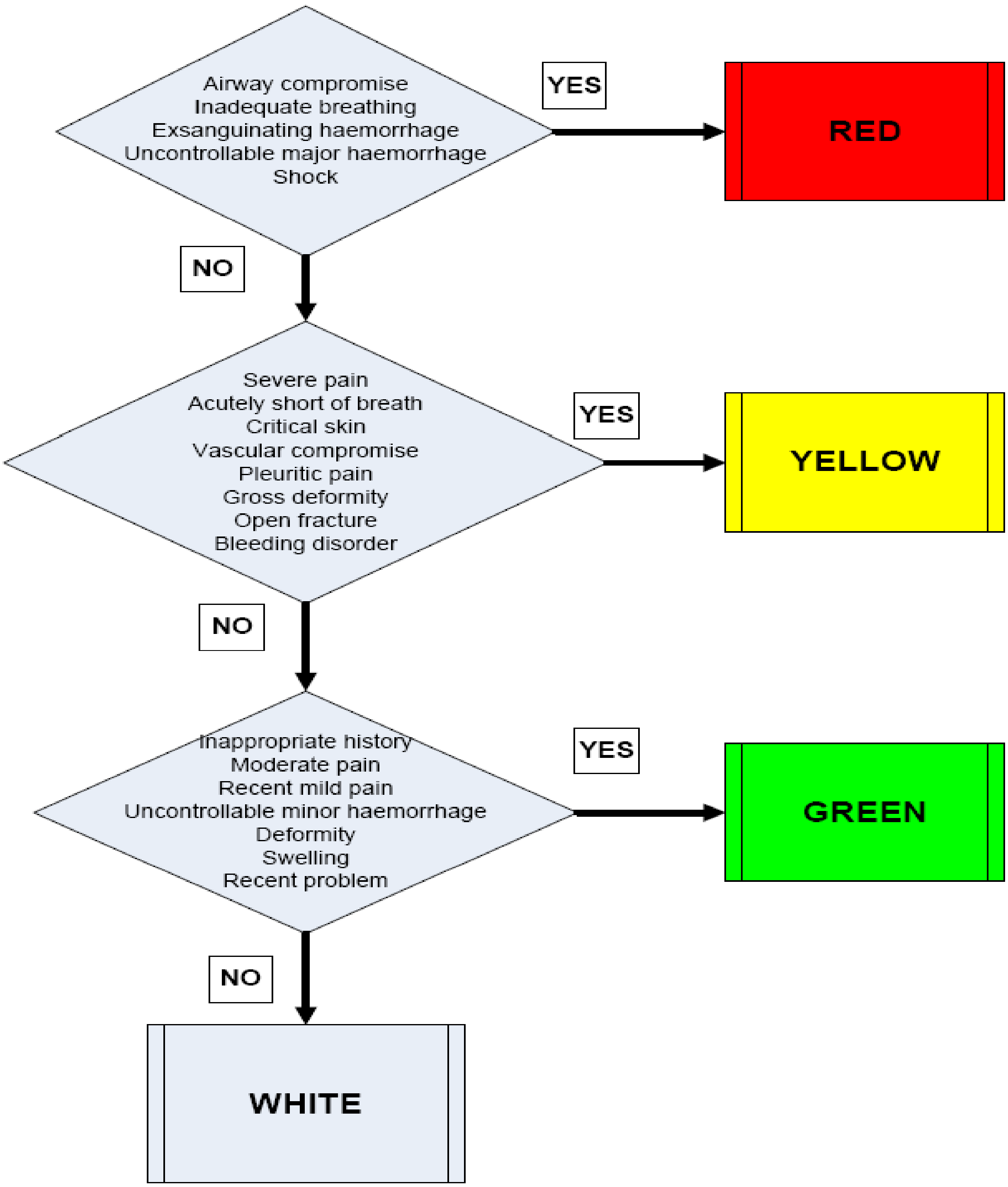

See also:	Chart notes:
Limping child	This is a presentation defined flow diagram. Injuries to the limbs are the commonest presentation to Emergency Departments and, while rarely life threatening, may cause considerable morbidity. A number of general discriminators are used including Life Threat, Haemorrhage and Pain. Specific discriminators are included to ensure that limb threatening injuries are seen and treated urgently. Discriminators are also included to remind the triage practitioner to consider the signs and symptoms of thromboembolic disease and its complications.

Major Trauma

Orotta TS - 01 June 2007 - V. 1.1

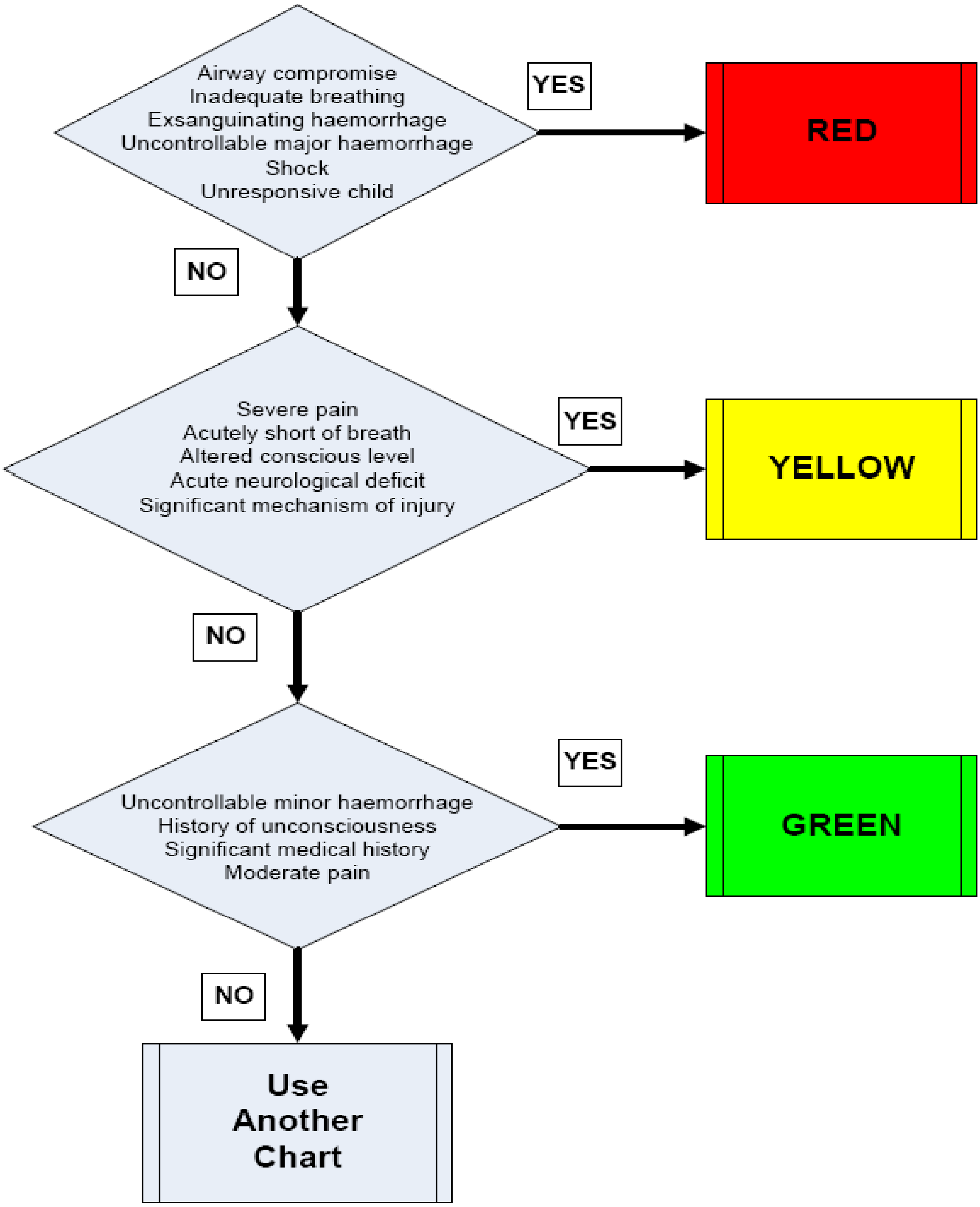

See also:	Chart notes:
	Most health care providers know what is implied by major trauma but it is a strange presentation in that it is defined not by the patient or their injury, but on some judgement of that injury by the carers. For this reason it is impossible to categorise a patient with this presentation as less than urgent. If it is necessary to do this then a deliberate decision needs to be made that the original description of the patient as having suffered major trauma was incorrect, and the patient should be categorised using a different presentational flow diagram. A number of general discriminators have been used including Life Threat, Haemorrhage, Conscious Level (both adult and child) and Pain. Specific discriminators are designed to ensure that patients with a significant mechanism of injury are given a high enough urgency, and that those with pre-existing medical conditions and or the development of new neurological signs are seen in good time.

Mental Illness

Orotta TS - 01 June 2007 - V. 1.1

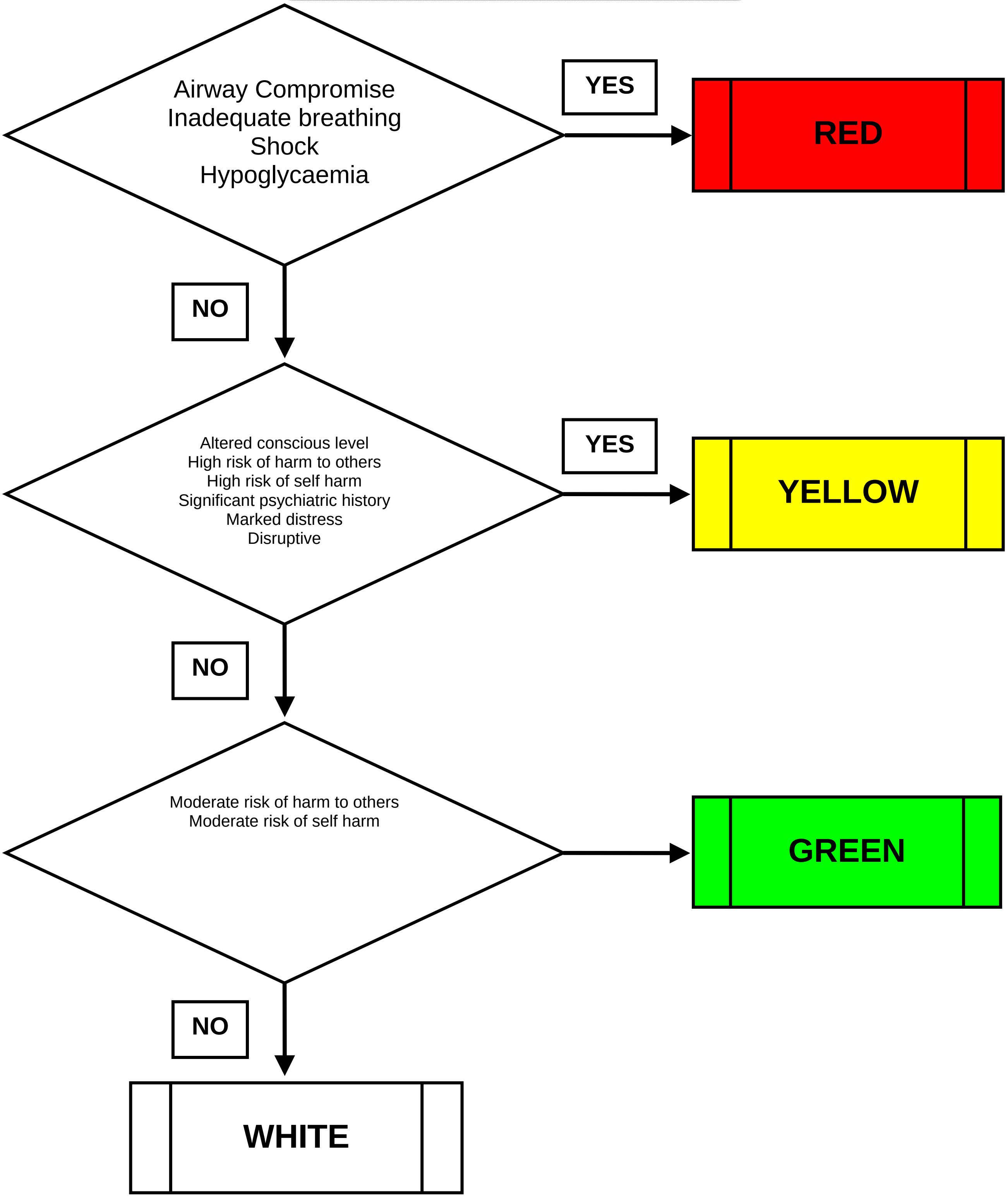

See also:	Chart notes:
Behaving strangely<br>Apparently drunk	This is a presentation defined flow diagram which has been designed to allow clinical prioritisation of patients who present with known or newly declared mental illness. This would include patients who attended with a chief complaint which would indicate mental illness. A number of general discriminators have been used including *Life Threat* and *Conscious Level.* This chart is designed to allow assessment of both physical and psychiatric aspects of the presentation. Specific discriminators are included to allow accurate prioritisation of patients with a known significant psychiatric history and those who have varying degrees of risk of causing harm to others or to themselves. Patients who are disruptive or who are suffering severe distress are placed in the urgent category.

Neck Pain

Orotta TS - 01 June 2007 - V. 1.1

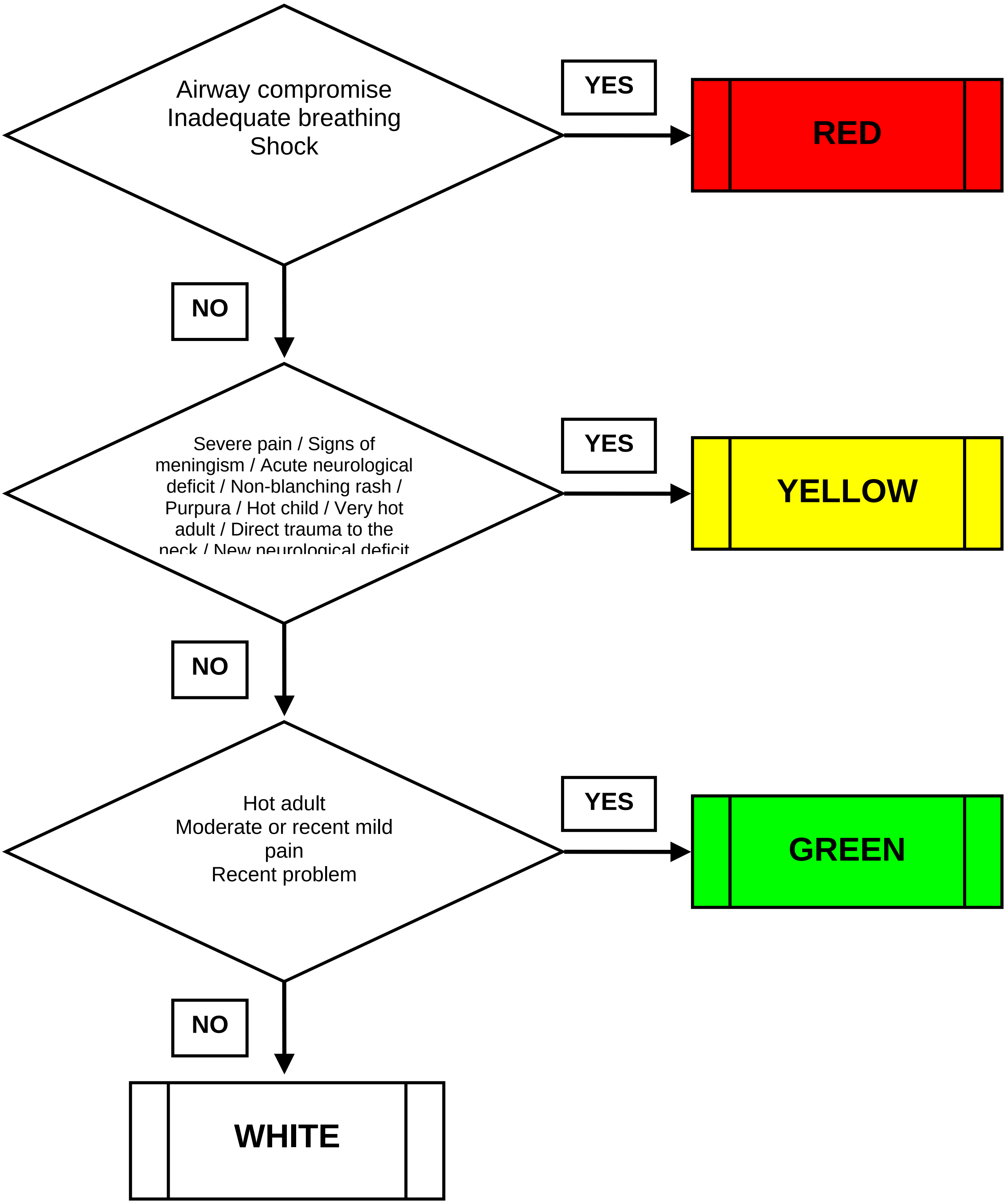

**See also:**	**Chart notes:**
Back pain<br>Headache	This is a presentation defined flow diagram. Pain in the neck may arise because of local pathology or because meningeal irritation. This chart is designed to allow rapid identification of patients presented with symptoms or signs which indicate more urgent pathologies. A number of general discriminators are used including *Life Threat, Pain and Temperature*. The specific discriminators which indicate meningitis are included under the YELLOW category.

Overdose and Poisoning

Orotta TS - 01 June 2007 - V. 1.1

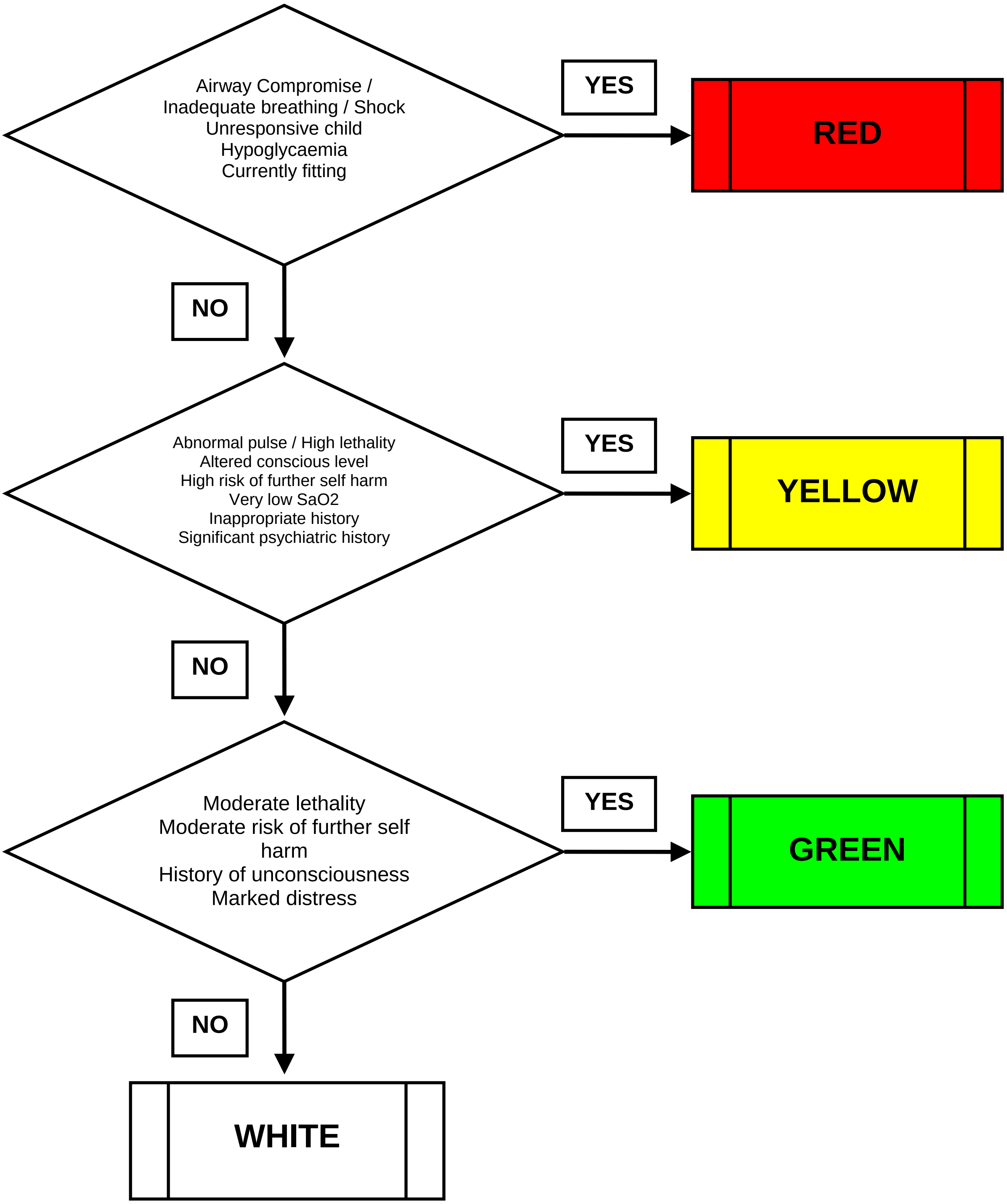

See also:	Chart notes:
	This is a presentation defined flow diagram. The flow chart has been designed to allow both the physical and psychiatric aspects of overdose to be considered, and to ensure accurate priorisation of patients from both perspectives. It also allows prioritisation of patients who have been accidentally (or deliberately) poisoned by others. A number of general discriminators have been used including *Life Threat and nconscious Level (in both children and adults)*. Specific discriminators include the assessed lethality of the overdose (which can be decided following discussion with a Poisons Centre) and an assessment of the risk of further attempts at self harm.

Palpitations

Orotta TS - 01 June 2007 - V. 1.1

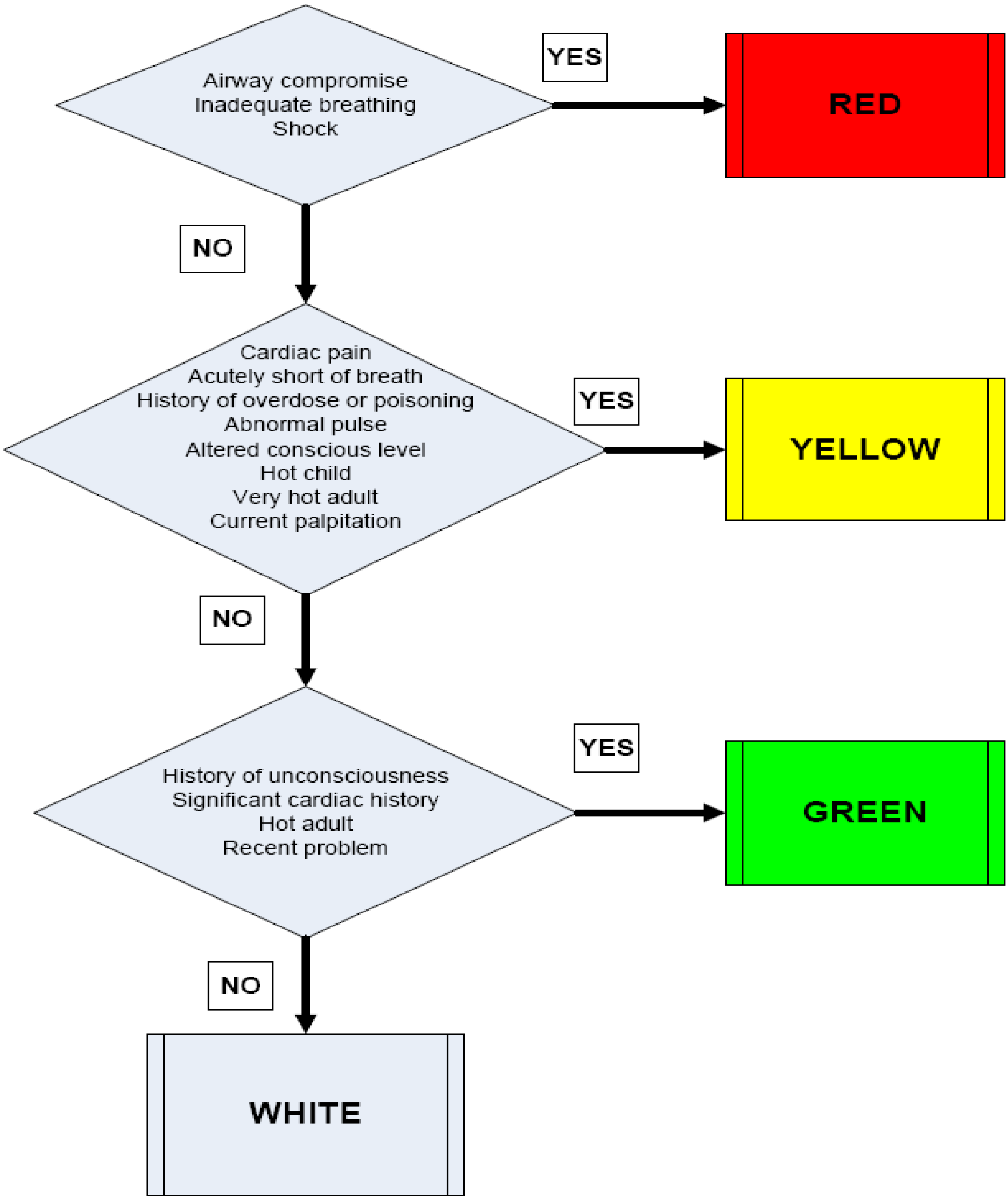

See also:	Chart notes:
Chest Pain<br>Unwell adult<br>Collapsed Adult	This is a presentation defined flow diagram designed to allow the accurate prioritisation of those patients that present with a chief complaint of palpitations. Palpitations can have many causes ranging from the effects of ischaemic heart disease and other cardiac abnormalities to anxiety. Whatever the cause it is their effect on circulation and their propensity to develop into life-threatening dysrhythmias that determines the clinical priority of the patient. Thus this chart is written to ensure that the signs and symptoms of cardiac insufficiency are included in the RED and YELLOW categories, together with historical pointers to potential early problems.

Pregnancy

Orotta TS - 01 June 2007 - V. 1.1

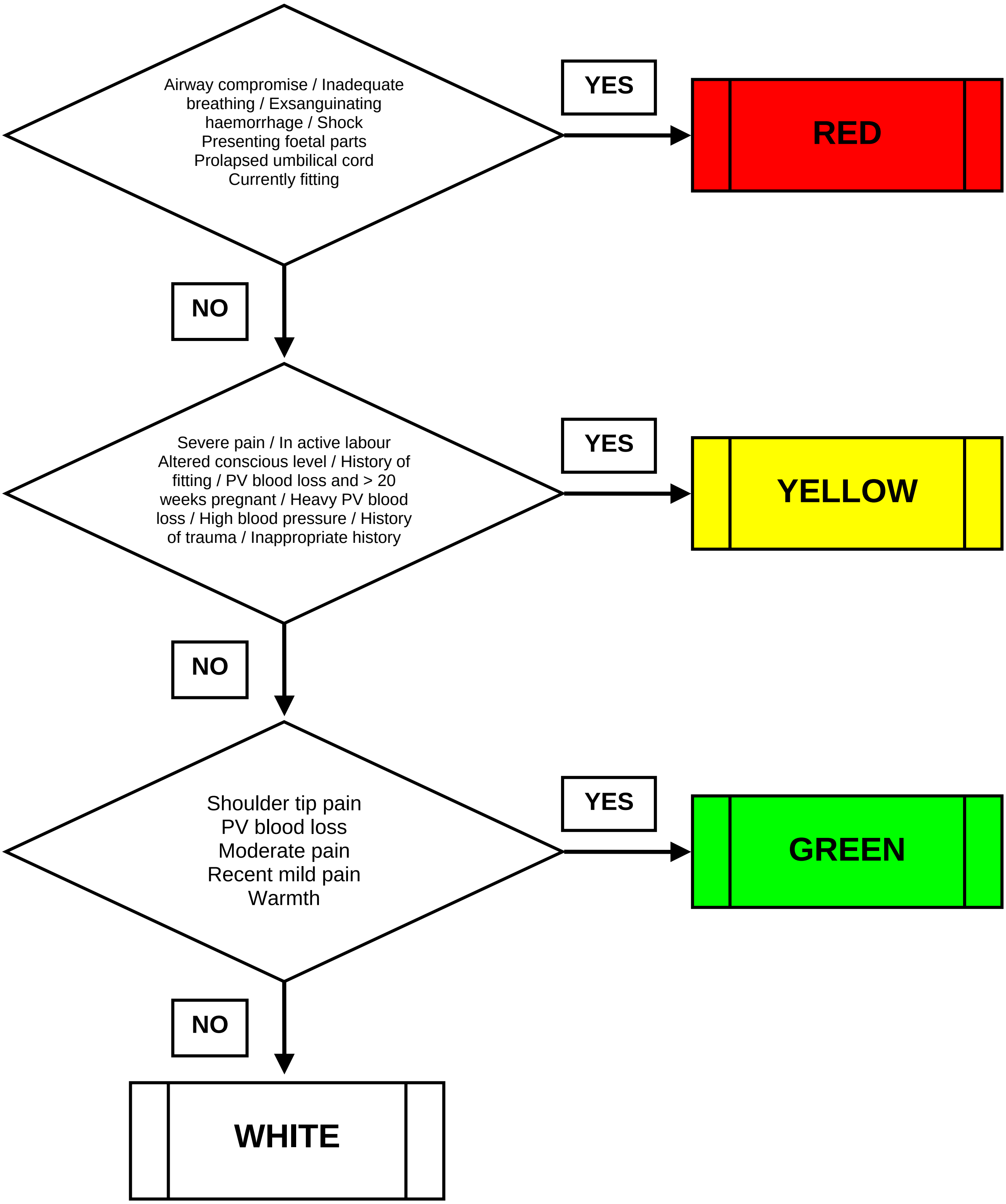

See also:	Chart notes:
PV bleeding	This is a presentation defined flow diagram. Pregnant women may attend the Emergency Department at all stages of pregnancy and with a variety of complaints. Some may be unaware of their pregnancy. A number of general discriminators have been used including *Pain* and *Conscious level*. Specific discriminators are designed to allow early recognition of complications of pregnancy at all stages.

PV (Vaginal) Bleeding

Orotta TS - 01 June 2007 - V. 1.1

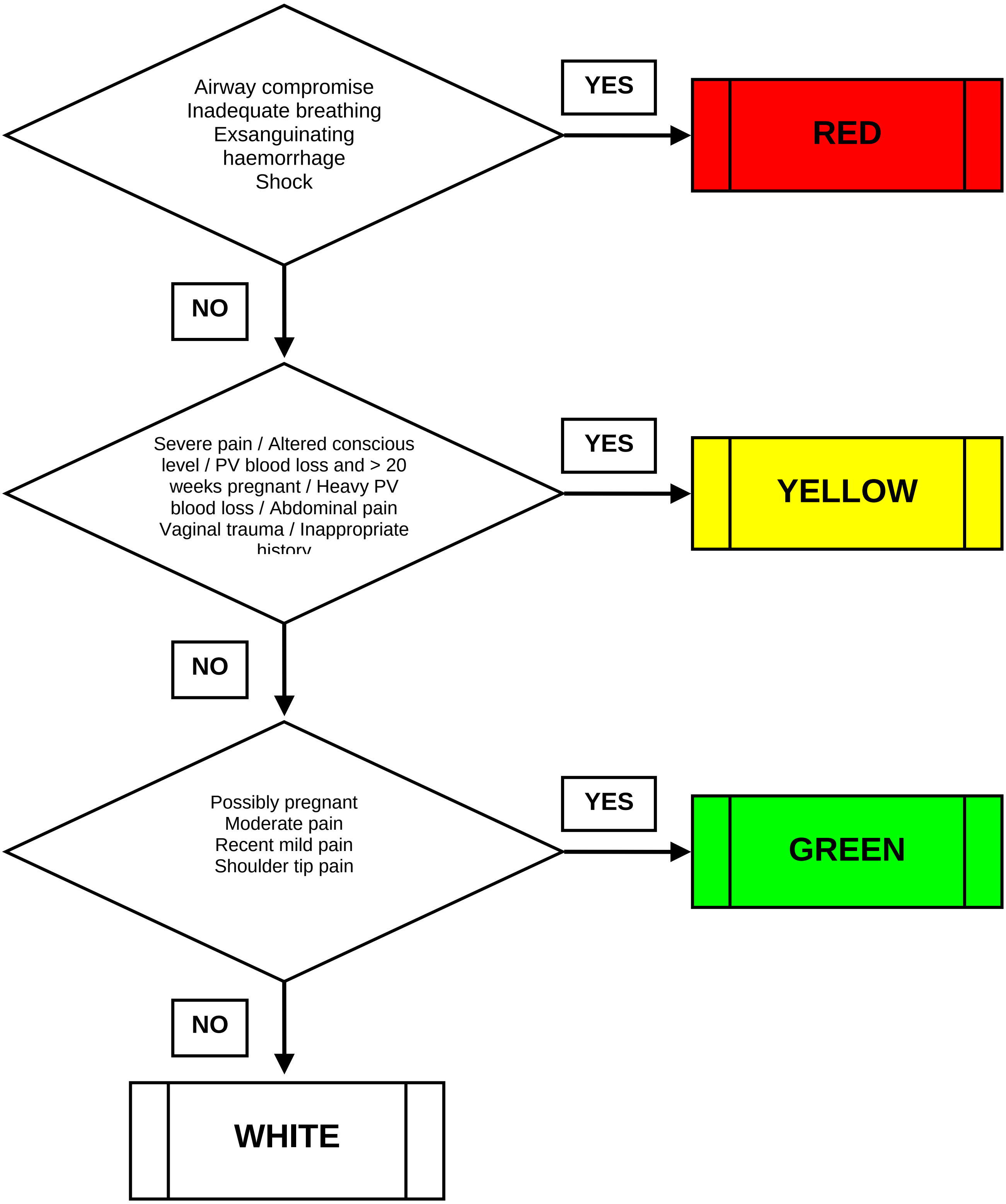

**See also:**	**Chart notes:**
Pregnancy<br>Abdominal pain	This is a presentation defined flow diagram. PV Bleeding may occur in pregnant and non-pregnant women and may have a large number of undefined causes. A number of general discriminators are used including *Life Threat, Haemorrhage, and Pain*.

Rashes

Orotta TS - 01 June 2007 - V. 1.1

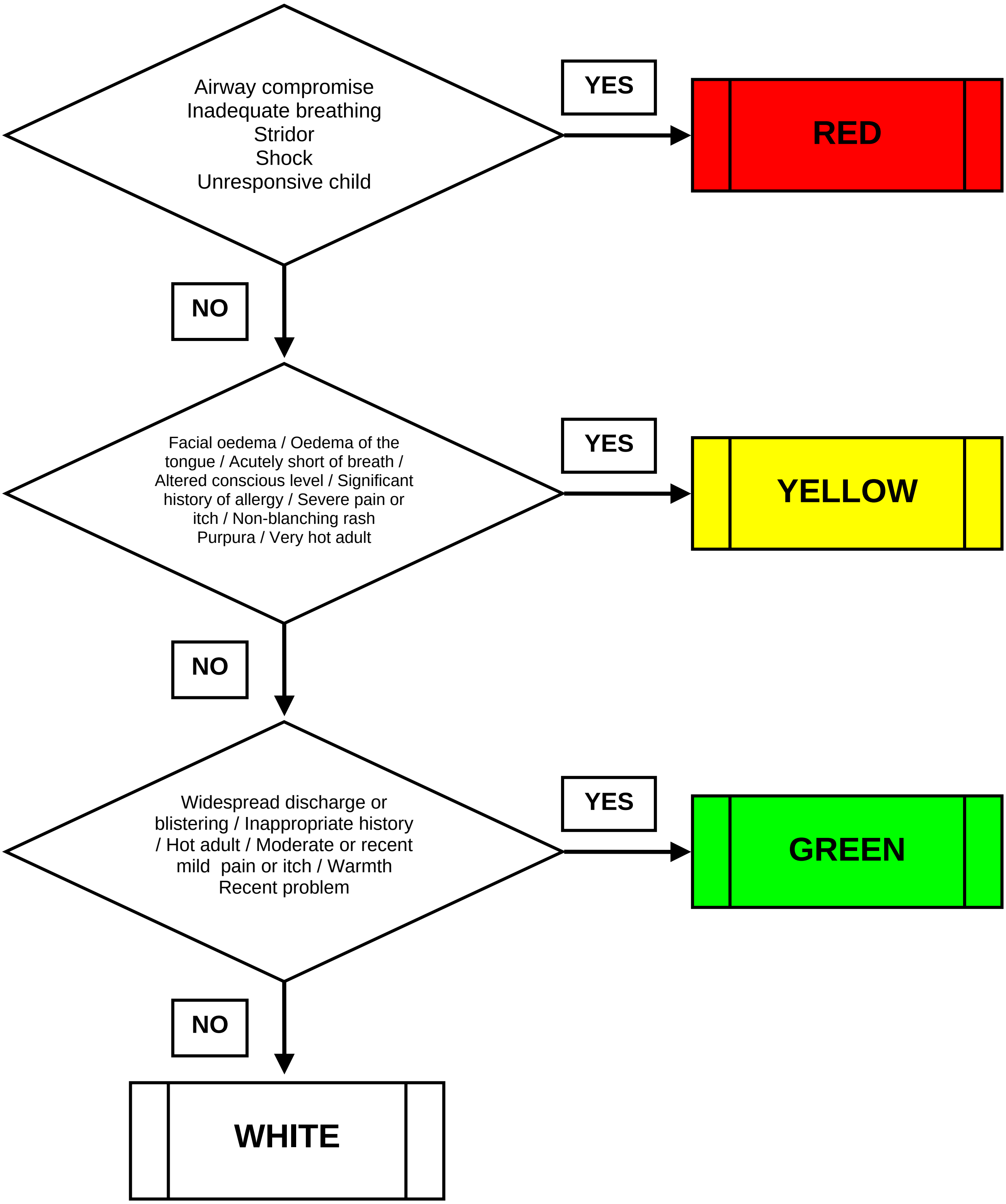

**See also:**	**Chart notes:**
Unwell Child<br>Unwell Adult<br>Allergy<br>Bites and Stings	This is a presentation defined flow diagram. A rash may signify serious disease such as meningococcal septicaemia, or may be a sign of a chronic non acute problem such as psoriasis. Two general discriminators - *Life Threat and Temperature* - are used in this chart. A larger number of specific discriminators are included in the YELLOW category to ensure that more serious conditions are suitably triaged. In particular purpura and associations of acute anaphylaxis appear at the YELLOW level.

Sexually Acquired Infections

Orotta TS - 01 June 2007 - V. 1.1

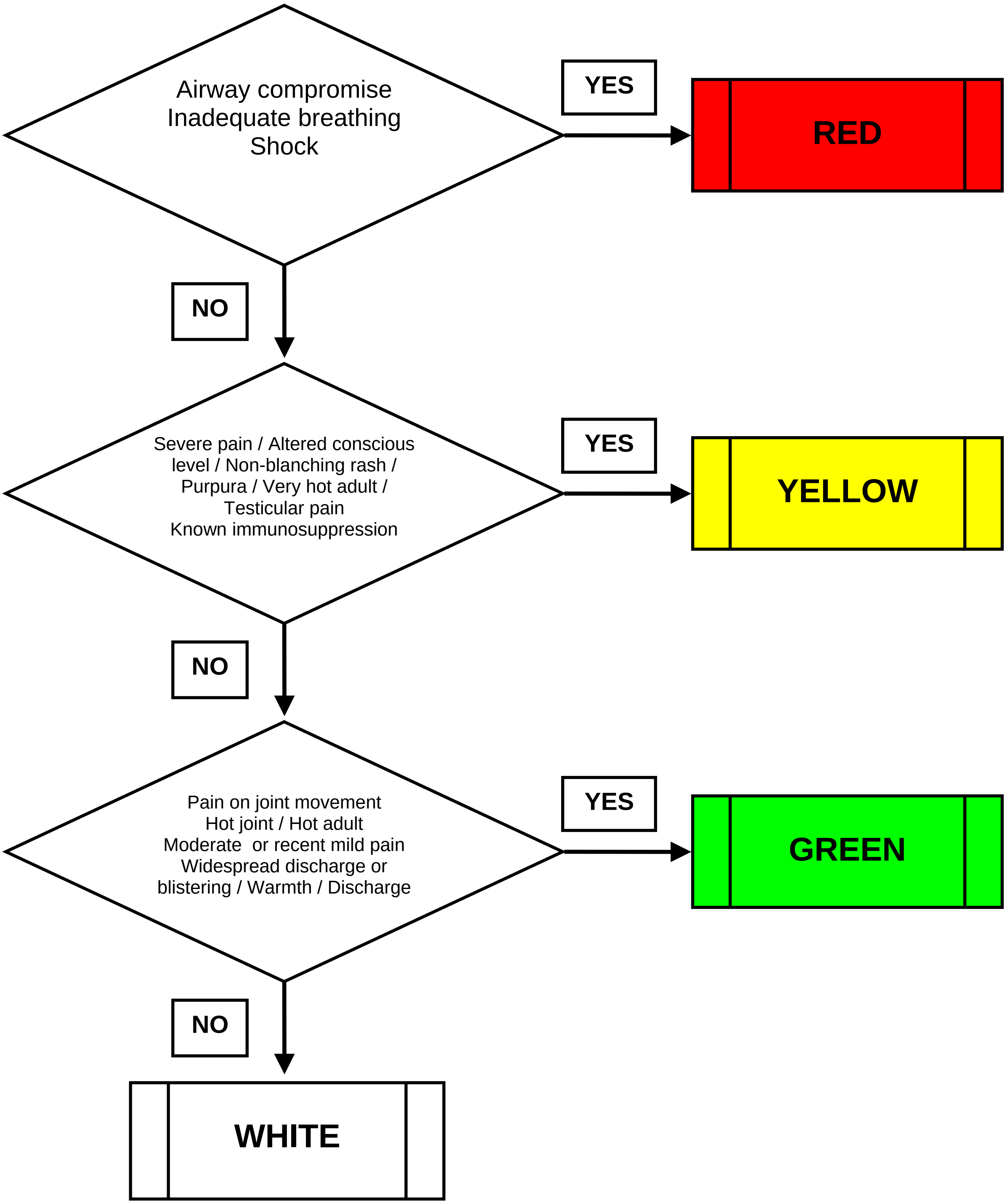

See also:	Chart notes:
	This is a presentation defined flow diagram which has been included to allow priorisation of patients who attend with known or obvious sexual acquired infection. A number of general discriminators are used including *Life Threat, Pain and Temperature*. Specific discriminators have been added to allow identification of more urgent conditions such as gonococcicaemia. It is important to ensure that preconceptions about disposal of these patients do not prevent appropriate triage.

Shortness of Breath

Orotta TS - 01 June 2007 - V. 1.1

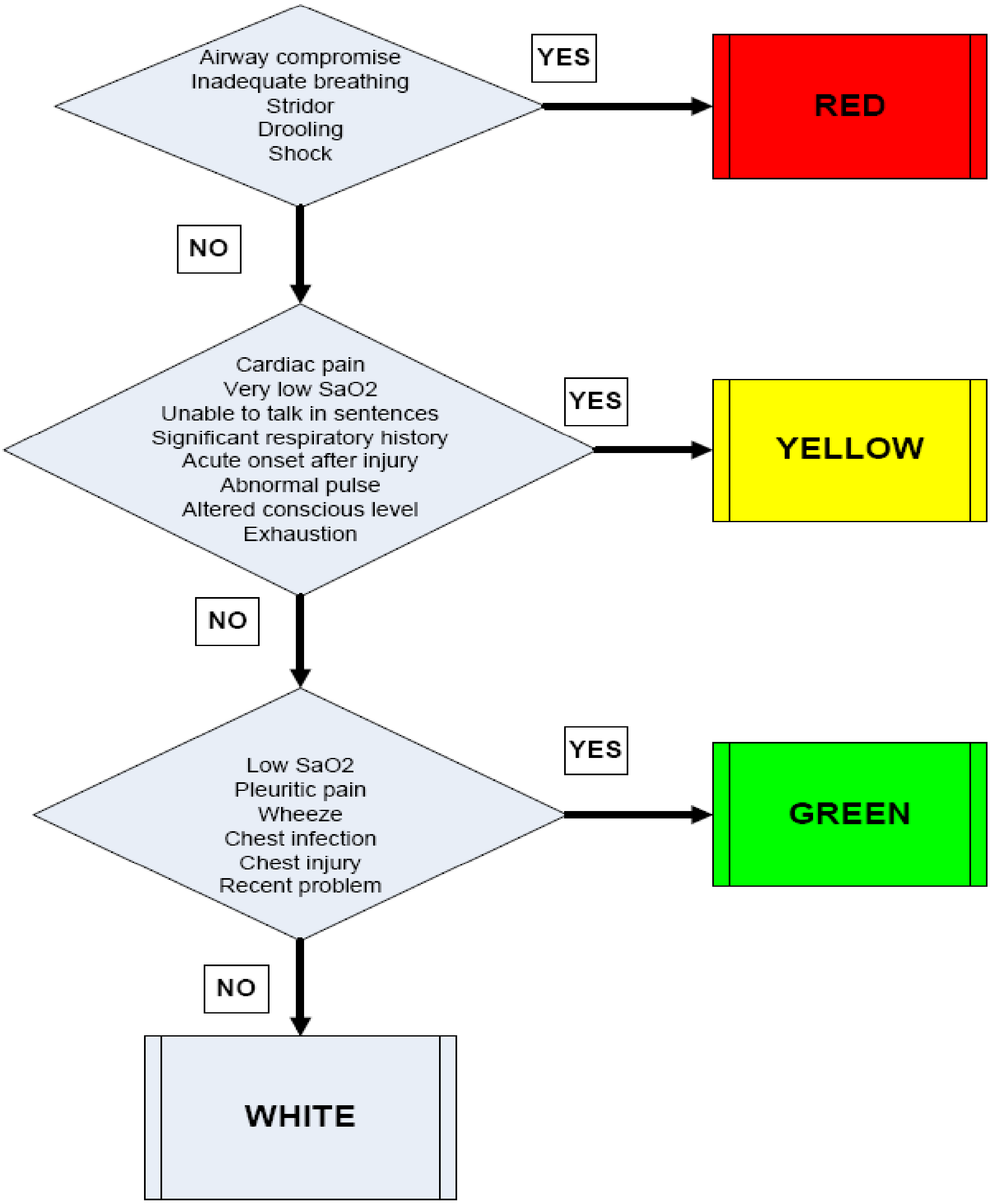

See also:	Chart notes:
Asthma<br>Shortness of breath in children<br>Unwell adult	This is a presentation defined flow diagram. Shortness of breath may be the presenting symptom for a number of cardiovascular and respiratory problems. A number of general discriminators are used including Life Threat and Oxygen Saturation. Specific discriminators include those which are present in severe asthma, chronic obstructive pulmonary disease and ischaemic heart disease.

Testicular Pain

Orotta TS - 01 June 2007 - V. 1.1

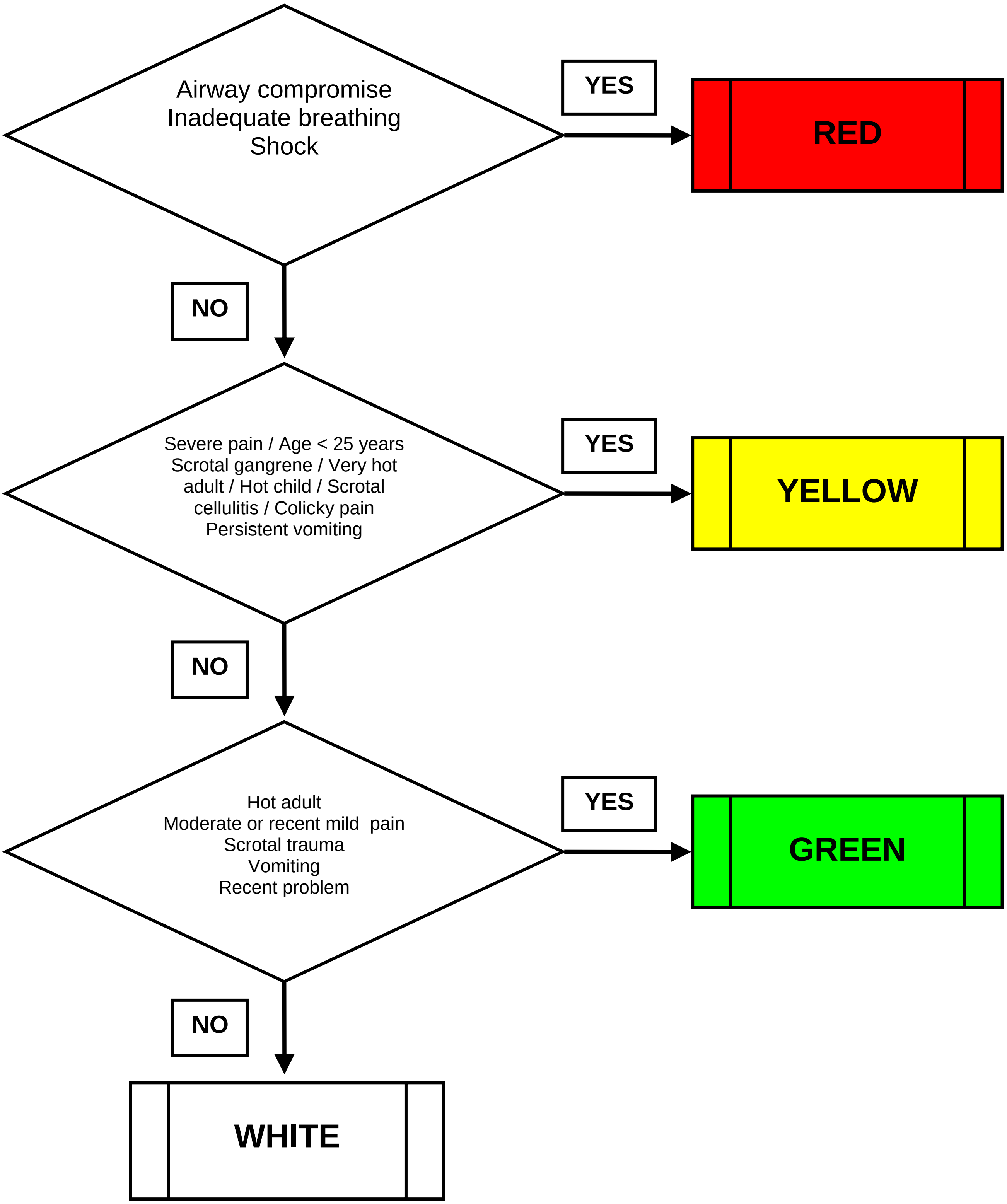

**See also:**	**Chart notes:**
Abdominal pain	This is a presentation defined flow diagram. Testicular pain may have a number of pathologies the most urgent of which is testicular torsion. A number of general discriminators are used including *Life Threat, Pain and Temperature*. Specific discriminators included in the YELLOW category are designed to indicate those patients who have a high chance or torsion of the testes and the most severe infections.

Torso Injury

Orotta TS - 01 June 2007 - V. 1.1

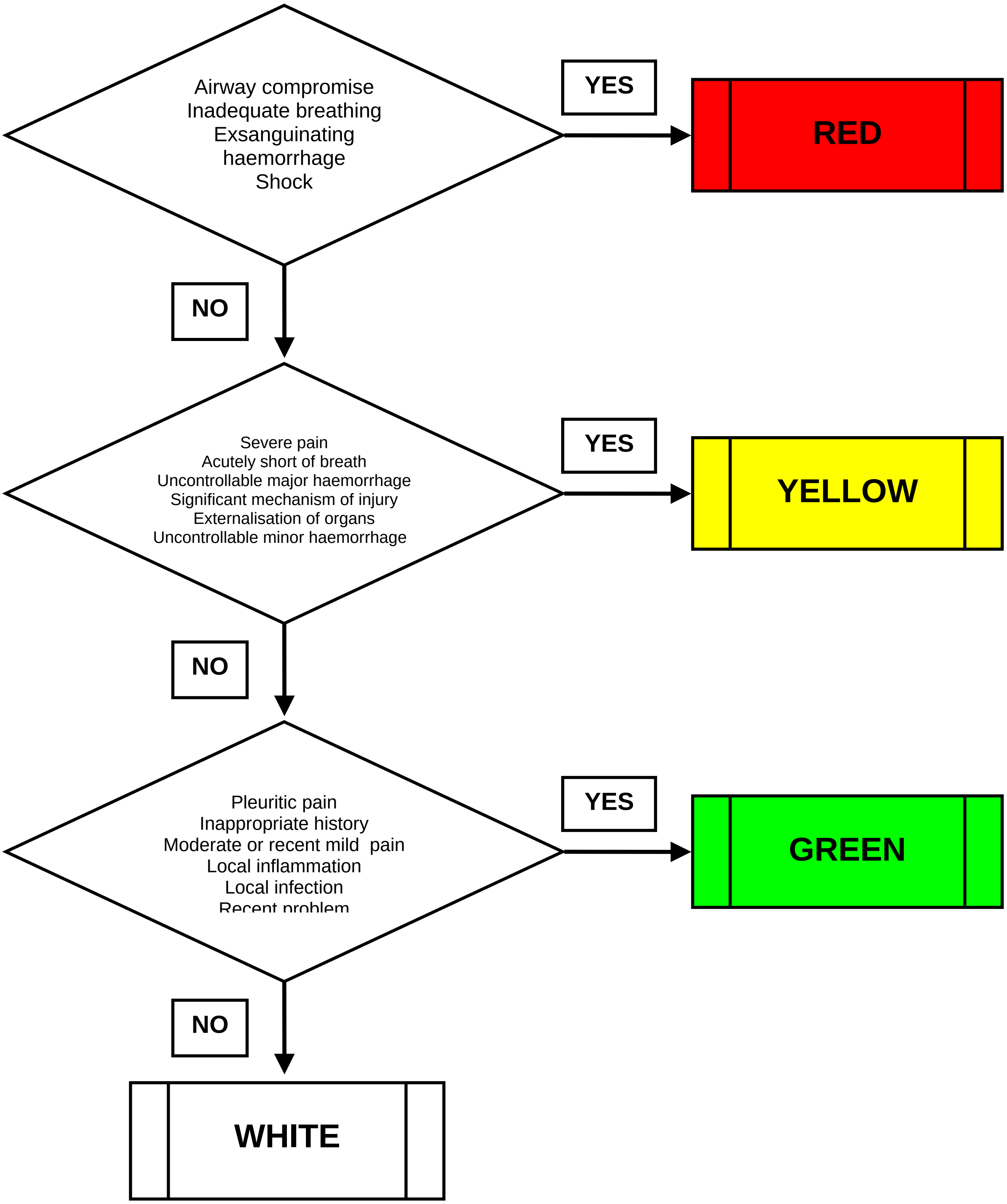

**See also:**	**Chart notes:**
Major Trauma<br>Assault<br>Wounds	This is a presentation defined flow diagram designed to allow accurate prioritisation of patients who have suffered injuries to the front or back of the chest and abdomen. A number of general discriminators are used including *Life Threat, Haemorrhage and Pain*. Specific discriminators have been used to allow identification of patients who are suffering from less obvious but severe internal injury. These would include patients who are acutely short of breath and those with a history suggestive of significant trauma.

Unwell Adult

Orotta TS - 01 June 2007 - V. 1.1

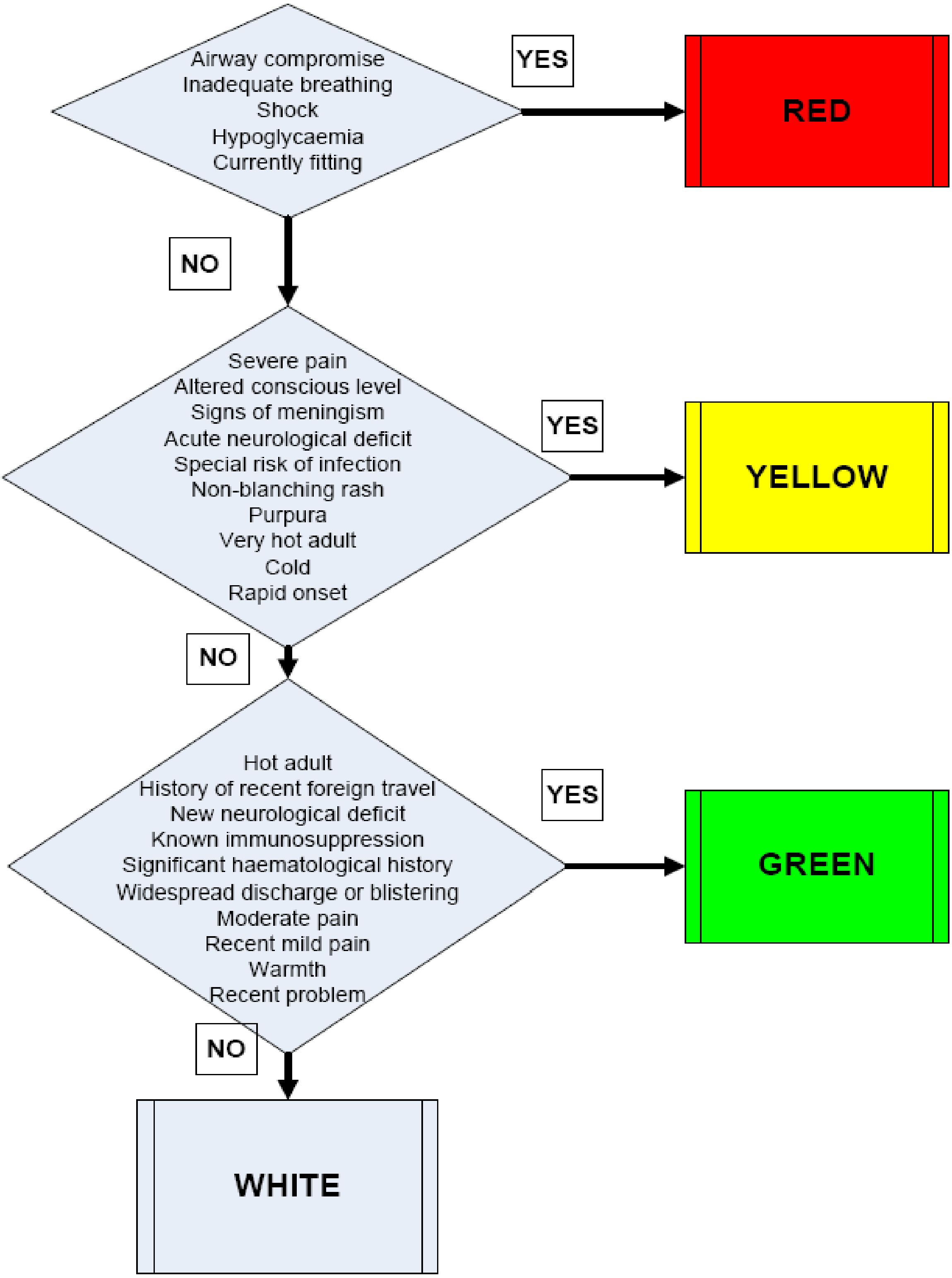

See also:	Chart notes:
Collapsed adult	This is a non specific presentation defined flow diagram. A number of general discriminators are used including Life Threat, Conscious Level, Pain and Temperature. Specific discriminators have been included to ensure that patients with, for example, meningococcicaemia are placed in the appropriate category.

Urinary Problems

Orotta TS - 01 June 2007 - V. 1.1

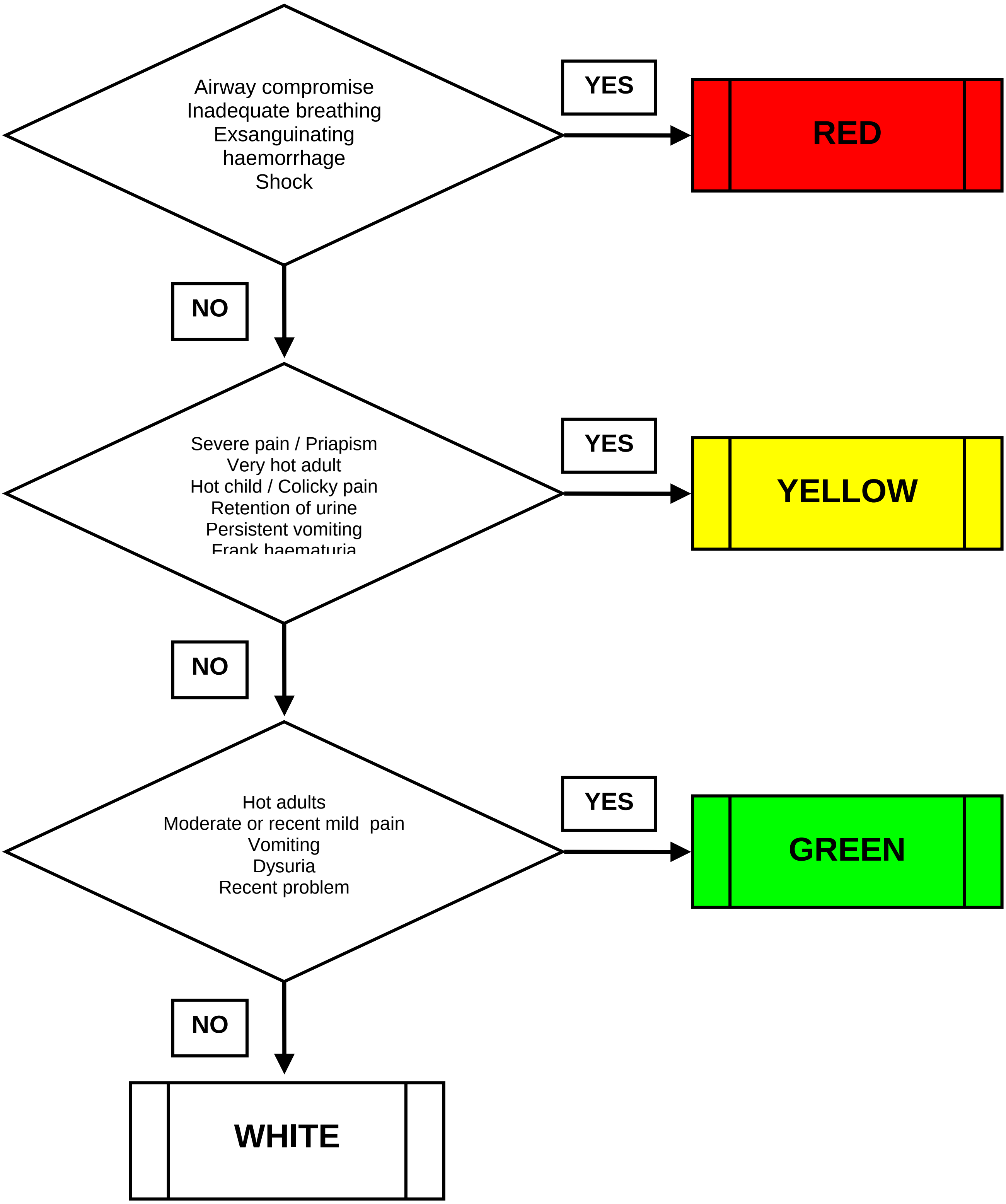

**See also:**	**Chart notes:**
Sexually acquired infection<br>Testicular pain	This is a presentation defined flow diagram. A lot of patients who present with urinary problems are in pain and some may have serious underlying pathology. A number of general discriminators are used including *Life Threat*, *Pain and Temperature*. Specific discriminators have been included to ensure that patients suffering from urinary retention and those with infections are included in the appropriate categories.

Wounds

Orotta TS - 01 June 2007 - V. 1.1

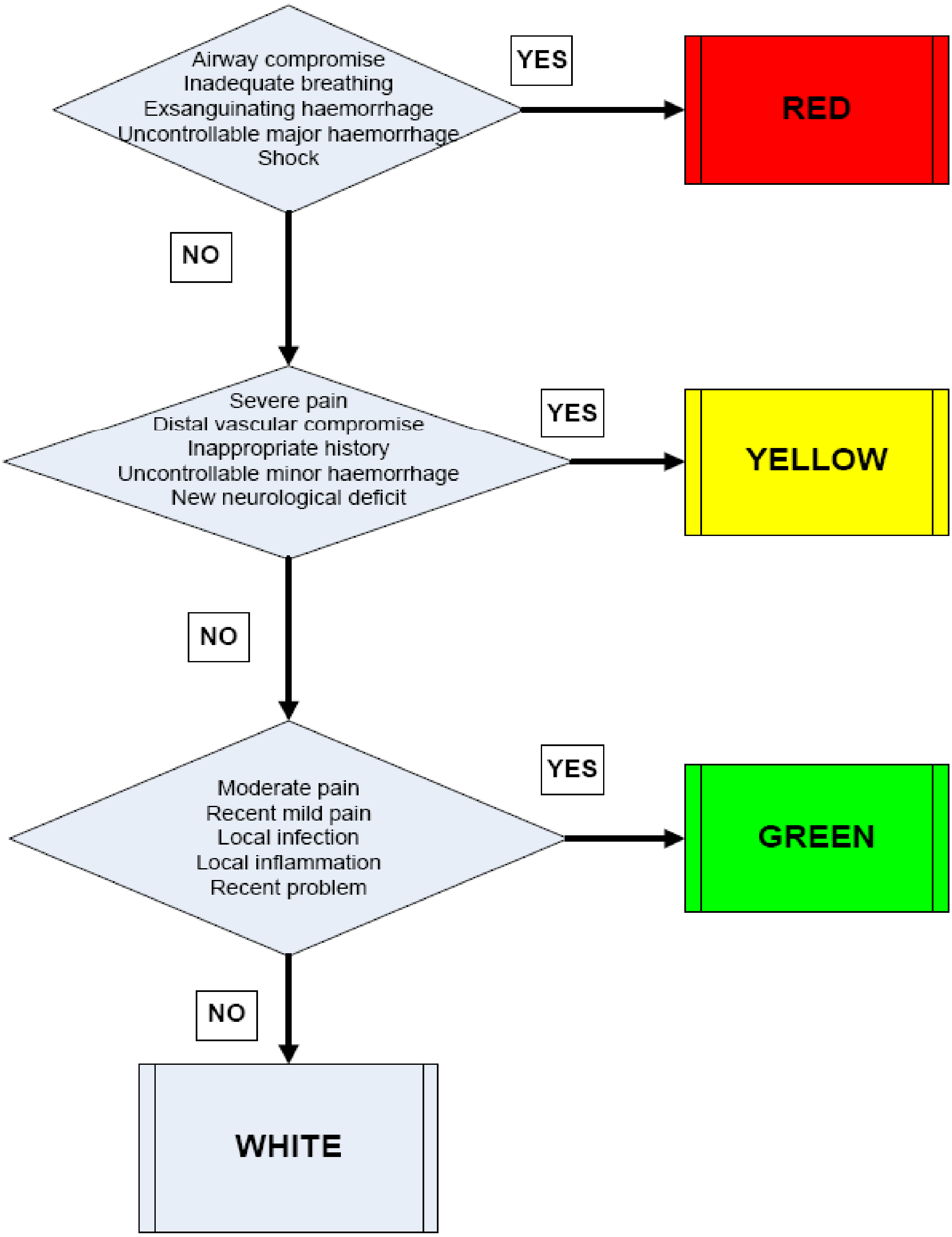

See also:	Chart notes:
Assault	This is a presentation defined flow diagram. Many patients attend all forms of emergency care suffering from wounds of various natures. These vary from severe life threatening lacerations to minor abrasions. This chart is designed to allow an accurate prioritisation of these patients. A number of general discriminators have been used including Life Threat, Haemorrhage and Pain. Specific discriminators have been included to allow identification of patients with signs and symptoms suggested of injuries which pose a threat to function.
